# A robust polyfunctional Pd(II)-based magnetic amphiphilic nanocatalyst for the Suzuki–Miyaura coupling reaction

**DOI:** 10.1038/s41598-021-89424-9

**Published:** 2021-05-13

**Authors:** Hamideh Aghahosseini, Mohammad Reza Saadati, Seyed Jamal Tabatabaei Rezaei, Ali Ramazani, Narges Asadi, Hidenori Yahiro, Masami Mori, Nahid Shajari, Ali Reza Kazemizadeh

**Affiliations:** 1 Department of Chemistry, Faculty of Science, University of Zanjan, Zanjan, 45371-38791 Iran; 2 Department of Biotechnology, Research Institute of Modern Biological Techniques (RIMBT, University of Zanjan, Zanjan, 45371-38791 Iran; 3 Department of Materials Science and Biotechnology, Graduate School of Science and Engineering, Ehime University, Matsuyama, 790-8577 Japan; 4 Department of Chemistry, Zanjan Branch, Islamic Azad University, P. O. Box 49195-467, Zanjan, Iran

**Keywords:** Catalysis, Green chemistry, Chemical synthesis

## Abstract

Herein, a robust Pd(II)-based polyfunctional magnetic amphiphilic artificial metalloenzyme was prepared by anchoring a Pd(2,2′-dipyridylamine)Cl_2_ bearing hydrophilic monomethyl ether poly(ethylene glycol) (mPEG) chains on the surface of amino-functionalized silica-coated magnetic nanoparticles. The 2,2′-dipyridylamine (dpa) has shown excellent complexation properties for Pd(II) and it could be easily anchored onto functionalized magnetic support by the bridging nitrogen atom. Moreover, the bridging nitrogen atom at the proximity of Pd(II) catalytic center could play an important role in dynamic suppramolecular interactions with substrates. The leaching, air and moisture resistant [Pd(dpa)Cl_2_] complex endow the dynamic and robust structure to the designed artificial enzyme. Moreover, the water dispersibility of designed artificial metalloenzyme raised from mPEG chains and the magnetic nanoparticles core which could function as protein mimics endow it other necessary characters of artificial enzymes. The prepared artificial metalloenzyme displayed remarkable activity in Suzuki–Miyaura cross-coupling reaction employing low-palladium loading under mild conditions, with the exceptionally high turnover frequency, clean reaction profile, easy work-up procedure, good to excellent products yields and short reaction times. The designed air- and moisture-stable artificial metalloenzyme could recycle more than fifteen times with easy separation procedure in aqueous solution under aerobic conditions without any noticeable loss in activity.

## Introduction

Biomimetic chemistry as a rapidly emerging research field in chemical sciences focuses on the simulation of biological function and processes particularly catalysis by purely chemical means^1, 2^. Recently, increasing attention has been given to design and synthesis of artificial enzymes as a very important and exciting branch of biomimetic chemistry^3^. Given the tremendous progress of nanotechnology, it has been found that, some nanomaterials exhibit unexpected enzyme-like activity^4–9^. Some individual characteristics of inorganic nanoparticles in terms of size, charge, shape and surface coating with organic functional groups could allow them to function like globular proteins and provide important similarities between them and natural enzymes^10^. The magnetic nanoparticles were introduced as supported materials with outstanding properties such as their ease of preparation and separation, diverse functionalization, chemical and hydrothermal stability and low cost^11^. Simple magnetic separation of superparamagnetic nanoparticles is a feature directly relevant to green chemistry goals, which solves the separation and recycling challenges in nanocatalysts and natural enzymes. Such interesting features have attracted the researchers’ attention in the field of artificial enzymes.

The design of robust catalysts is important for catalytic systems in the view of green chemistry^12^. Typically Pd(0) complexes are known as the active catalytic species during cross-coupling reactions, while their inherent instability to air and moisture compared with Pd(II) complexes has limited their use in such reactions^13^. On the other hand, few instances have employed Pd(II) species as catalytic centers in the Pd(II)/Pd(IV)-based mechanism^14–17^. It is because the formation of organopalladium(IV) complexes needs to the presence of the strong donor ligands to stabilize them^18^. Despite the fact that the proposal of oxidation mechanism of Pd(II) to Pd(IV) by MeI has been approved with X-ray crystallography as a strong evidence^19–21^, but few studies have been done in this field. It seems that the question of which of Pd(0)/Pd(II) or Pd(II)/Pd(IV) forms the basis for the oxidative addition/reductive elimination cycle seems to be very complex. In fact, things like the presence of strongly stabilizing ligands or spectator ligands can affect the mechanism. The use of strongly stabilizing ligands may yield catalysts with very high turnover number and high reactivity, but generally only under vigorous conditions which are believed to be associated with slow reduction/dissociation of the precursor complex^22^. Moreover, there is also the issue of the coordination of spectator ligands at Pd, and as a consequence the charge on the catalytic intermediates may vary under different reaction conditions^23, 24^.

Whilst, the Pd(II)/Pd(IV) cycle is more favorable for the facile *reductive elimination* and more chemoselective for the *oxidative addition*^18, 25^. The strong σ-donation assistances these steps to develop air- and moisture-stable Pd(II)-based catalysts which are suitable for aerobic conditions in aqueous media^26, 27^. *N*-donor ligands could maintain proper electron density at metal center^28^. The *N*,*N*-type ligands like dipyridyls have shown excellent complexation properties for Pd and they could minimize the Pd leaching in reaction media^28^. The potentially *N*,*N*-donor ligand 2,2′-dipyridylamine (dpa) belongs to the polydentate nitrogen ligands family. The superior properties of dpa compared with the other *N*,*N*-type ligands mainly raised from the bridging nitrogen atom in its structure which could modulate the electronic and steric properties^28^. The dpa could be easily anchored onto a suitable support by the bridging nitrogen atom as an anchorage point to prepare supported catalysts. Moreover, the bridging nitrogen atom at the proximity of a catalytic center which was coordinated by dipyridyls could play an important role in substrate orientation and activation and product release through hydrogen-bonding in the dpa-based complexe^28^. Such dynamic suppramolecular interactions which are essential factors in rational design of artificial enzymes persuaded us to design and prepare highly efficient [Pd(dpa)Cl_2_]-based polyfunctional magnetic amphiphilic artificial metalloenzyme. Considering supramolecular interactions, some artificial enzymes have been designed and synthesized. Some of them designed based on the hydrophobic interactions in their supramolecular structures. In such systems Breslow et al*.*^29^ proposed a supramolecular substrate preorganization strategy with hydrophobic interactions. Moreover, a similar supramolecular substrate preorganization strategy by hydrogen bonding interactions has been developed^30–32^. The importance of such supramolecular interactions in regioselectivity of reactions, were examined by experiments and DFT calculations. These investigations demonstrated that the undesired product is blocked by host–guest interactions, whereas the desired product is lowered in energy because of the suitable length of the substrate.

The prepared artificial metalloenzyme was employed successfully in Suzuki–Miyaura cross-coupling reaction as one of the most important and versatile transformations in biaryl synthesis^33–35^. Many of the Suzuki–Miyaura reactions catalyzed by palladium salts or complexes are performed in harmful organic solvents^36–40^. On the other hand, the major drawback associated with supported catalysts in aqueous reaction media is the poor interaction between active sites of catalysts and organic substrates. Hence, there are many reports on Suzuki–Miyaura reaction promoted by various supported catalysts in harmful organic solvents^41–43^. Hereupon, some strategies were proposed to solve this problem, such as the development of ionic liquids- or amphiphilic polymers-based supported catalysts^44–50^. Although these types of catalysts have shown good features, many of them still suffer from hard reaction conditions, higher reaction time or lack of durability/stability of catalysts.

Palladium(0) complexes and palladium nanoparticles were employed widely as the catalysts of the Suzuki–Miyaura reaction^51–53^. As it was mentioned these catalysts typically have low stability and furthermore, the high activity of palladium nanoparticles reduces their selectivity significantly. In this regard, Cuenca et al*.* reported a palladium-based *artificial metelloenzyme*, which was prepared from *Candida antarctica B* lipase and palladium salt^54^. The prepared palladium nanoparticles enzyme aggregate used for the Suzuki–Miyaura cross-coupling reaction in a mixture of methanol/water (1:1). It was reported that the designed *artificial metelloenzyme* did not show high yields in 2 to 24 h and it could be recycled 4 times.

To the best of our knowledge, there is no report on the application of supported catalysts based on robust Pd(II) complexes with the evidences to propose more favorable and chemoselective Pd(II)/Pd(IV)-based mechanism in the Suzuki–Miyaura reaction.

Hence, it seems that there is still room for developing novel robust Pd(II)-based supported catalysts with special features which were designed directly relevant to green chemistry goals.

In our designed polyfunctional artificial metalloenzyme, amphiphilicity originates from the covalent attachment of the hydrophilic monomethyl ether poly(ethylene glycol) (mPEG) chains to the lipophilic [Pd(dpa)Cl_2_] complex via cyanuric chloride as a cross-linking agent. The unique properties of dpa ligand beside the leaching, air and moisture resistant [Pd(dpa)Cl_2_] complex endow the dynamic and robust structure to the designed artificial enzyme. Moreover, the water dispersibility of designed artificial metalloenzyme raised from mPEG chains and the magnetic nanoparticles core which could function as protein mimics^10^ endow it other necessary characters of artificial enzymes. The prepared artificial metalloenzyme represented good efficiency and selectivity to produce corresponding Suzuki–Miyaura cross-coupling products under mild conditions in aqueous media and no self-coupling of aryl boronic acids were detected.

The investigation of the reaction mechanism indicated that a more favorable and chemoselective Pd(II) to Pd(IV) route could be a plausible mechanism which was supported by X-ray photoelectron spectroscopy analysis.

The designed air- and moisture-stable artificial metalloenzyme could recycle more than fifteen times in aqueous solution under aerobic conditions without any noticeable loss in activity.

## Results and discussion

**Figure 1:**
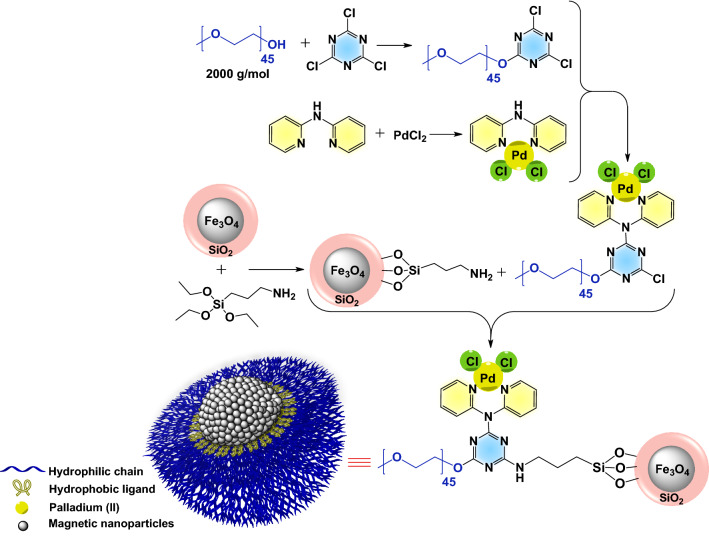
Schematic procedure for the preparation of MNPs@SiO_2_-NH_2_@Pd(dpa)Cl_2_ nanocatalyst.

The designed Pd(II)-based polyfunctional magnetic amphiphilic artificial metalloenzyme was synthesized via covalent attachment of the mPEG-anchoring [Pd(dpa)Cl_2_] complex to amino-functionalized silica-coated magnetic nanoparticles (Fig. 1). The preparation processes were confirmed with NMR and FTIR analyses (ESI, Figure S1-S11). The ^1^H NMR spectrum of 2-mPEG-4,6-dichloro-1,3,5-triazine confirms successful addition of 2,4,6-trichloro-1,3,5-triazine to mPEG (ESI, Figure S11, A). Disappearance of NH peak in 2-mPEG-4-[Pd(dpa)Cl_2_]-6-chloro-1,3,5-triazine spectrum in 7.99 ppm comparing with Pd(dpa)Cl_2_, confirms the covalent linkage of Pd(dpa)Cl_2_ to 2-mPEG-4,6-dichloro-1,3,5-triazine (ESI, Figure S11, B and C).

**Figure 2:**
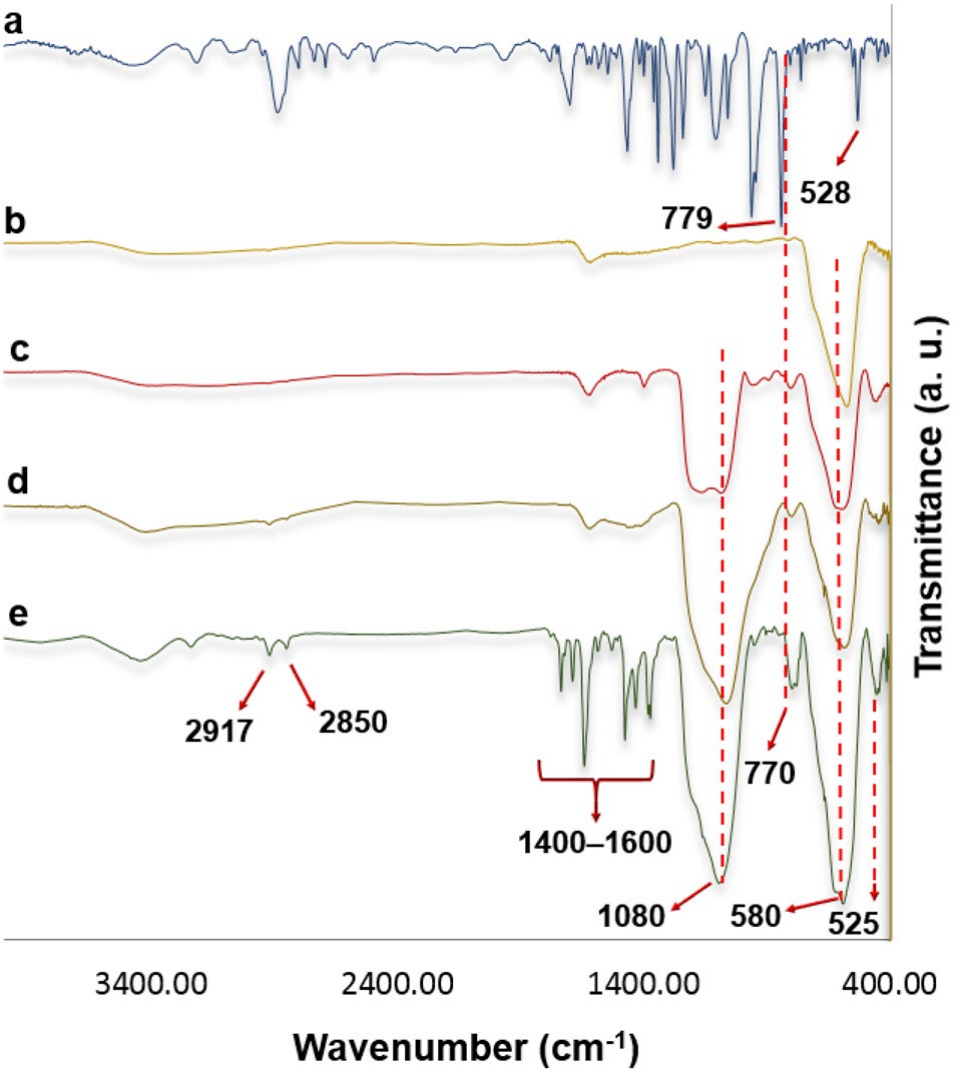
FTIR spectra of: (**a**) 2-mPEG-4-(Pd(dpa)Cl_2_)-6-chloro-1,3,5-triazine, (**b**) MNPs, (**c**), MNPs@SiO_2_ (**d**) MNPs@SiO_2_-NH_2_, and (**e**) MNPs@SiO_2_-NH_2_@Pd(dpa)Cl_2._

The infrared analysis of MNPs@SiO_2_-NH_2_@Pd(dpa)Cl_2_ and all of its precursors is shown in Fig. 2. The Fe–O stretching band as a characteristic peak of magnetite nanoparticles could be observed in Fe_3_O_4_ and functionalized Fe_3_O_4_ nanoparticles at 580 cm^−1^ (Fig. 2b–e). The modification of Fe_3_O_4_ by tetraethoxysilane (MNPs@SiO_2_) and its amino-functionalization with (3-aminopropyl)triethoxysilane (MNPs@SiO_2_-NH_2_) create strong and broad peaks at 1000–1100 cm^–1^ associated with Si-(OH) stretching vibration (Fig. 2c–e). The distinct changes of the MNPs@SiO_2_-NH_2_ before and after combination with 2-mPEG-4-(Pd(dpa)Cl_2_)-6-chloro-1,3,5-triazine in the FTIR spectra could be observed in some absorption bands around 770 cm^-1^ and in the region of 1400–1600 cm^−1^ which are attributed to the vibrations of pyridyl rings^55^ and the Pd–N stretching frequencies ranged from 528 to 436 cm^−l^ (Fig. 2a,e)^56^. Moreover, the intensity growth of –CH_2_– stretching peaks at 2850 and 2917 cm^−1^ (Fig. 2d, e), which are related to the mPEG is the other evidence that emphasis on the synthesis of MNPs@SiO_2_-NH_2_@Pd(dpa)Cl_2_.

The Thermogravimetric (TG) and derivative thermogravimetric (DTG) curves of MNPs@SiO_2_-NH_2_@Pd(dpa)Cl_2_ artificial metalloenzyme and MNPs@SiO_2_-NH_2_ before functionalization with 2-mPEG-4-(Pd(dpa)Cl_2_)-6-chloro-1,3,5-triazine are represented in Figure S12. The thermal decomposition profile of MNPs@SiO_2_-NH_2_@Pd(dpa)Cl_2_ represented three distinct steps of weight losses upon heating from room temperature to 800 °C under airflow (Figure S12 c and d). The first and second weight loss steps at 83 and 299 °C are due to the removal of solvents adsorbed on the surface as well as structural water molecules within amorphous SiO_2_ layers. The third weight loss at 605 °C indicated the cleavage of 2-mPEG-4-(Pd(dpa)Cl_2_)-6-chloro-1,3,5-triazine moiety of the MNPs@SiO_2_-NH_2_@Pd(dpa)Cl_2_ structure. This weight loss step revealed that the weight percentage of 2-mPEG-4-(Pd(dpa)Cl_2_)-6-chloro-1,3,5-triazine moiety was about 15% and the Pd(II) percentage was about 1% consequently which is corresponded well to the inductively coupled plasma mass spectroscopy (ICP-MS; 1.05% Pd).

**Figure 3:**
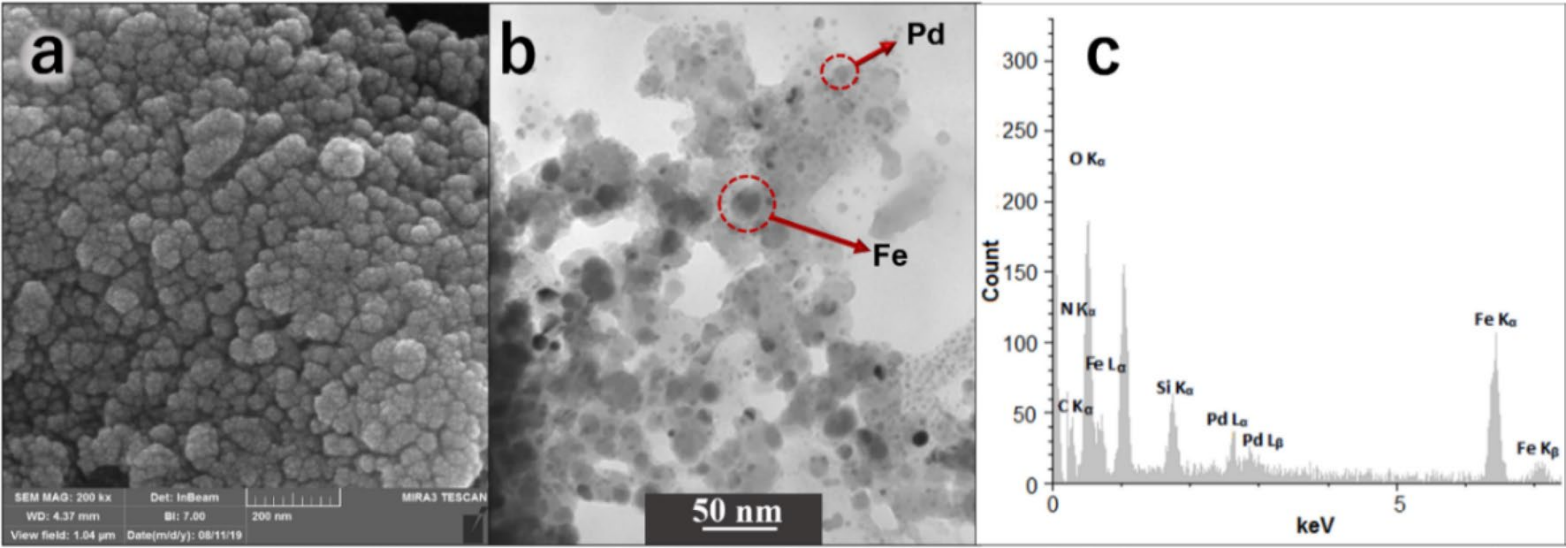
SEM (**a**), and FE-TEM (**b**), analyses and EDS spectrum (**c**) of MNPs@SiO_2_-NH_2_@Pd(dpa)Cl_2_ nanoparticles.

Microscopic properties of MNPs@SiO_2_-NH_2_@Pd(dpa)Cl_2_ nanoparticles were characterized by scanning electron microscope (SEM) and field emission transmission electron microscopy combined with an energy-dispersive X-ray spectroscopy (FE-TEM/EDS) analyses (Fig. 3a,b and Figure S17, S18 and Table S2). The SEM analysis revealed their spherical and uniform particle structures. The (FE-TEM/EDS) employed to provide the morphological characteristics of chemical composition of MNPs@SiO_2_-NH_2_@Pd(dpa)Cl_2_. Electron diffraction data which were obtained on single MNPs@SiO_2_-NH_2_@Pd(dpa)Cl_2_ nanoparticles indicated that the large black spots in Fig. 3b contained a large amounts of Fe which could represent magnetic nanoparticles, and small dark gray spots seen on a light gray halo around large black spots indicate the presence of palladium located on the polymer chains around magnetic nanoparticles. The average particle diameter of about 20 nm was obtained for MNPs@SiO_2_-NH_2_@Pd(dpa)Cl_2_. Moreover, the EDS represented the C, N, O, Si, Fe and Pd signals as the principal elements of MNPs@SiO_2_-NH_2_@Pd(dpa)Cl_2_ nanocatalyst (Fig. 3c).

The magnetic hysteresis loops for the MNPs@SiO_2_-NH_2_ and MNPs@SiO_2_-NH_2_@Pd(dpa)Cl_2_ nanoparticles were recorded at room temperature. Figure S13 shows that the MNPs@SiO_2_-NH_2_ and MNPs@SiO_2_-NH_2_@Pd(dpa)Cl_2_ nanoparticles exhibit superparamagnetic behavior at room temperature. The saturated mass magnetization of the MNPs@SiO_2_-NH_2_ and MNPs@SiO_2_-NH_2_@Pd(dpa)Cl_2_ are about 61.3 and 53 emu g^−1^ respectively. These values show their high permeability and consequently indicate simple magnetic recoverability of MNPs@SiO_2_-NH_2_@Pd(dpa)Cl_2_ nanocatalyst from the reaction media.

The X-ray diffraction (XRD) pattern of prepared MNPs@SiO_2_-NH_2_@Pd(dpa)Cl_2_ represented strong and sharp peaks that can be ascribed to the pure phase of magnetite in its structure (Figure S14). Additionally, the diffraction peaks of nanostructured palladium complex can be observed over the MNPs@SiO_2_-NH_2_@Pd(dpa)Cl_2_ sample. According to the Scherrer equation [*D* = K*λ*/(*β*cos*θ*)] the crystallite size of MNPs and nanostructured palladium complex at their highest diffraction lines (35.61° and 38.08°) were 15 and 30 nm respectively.

**Figure 4:**
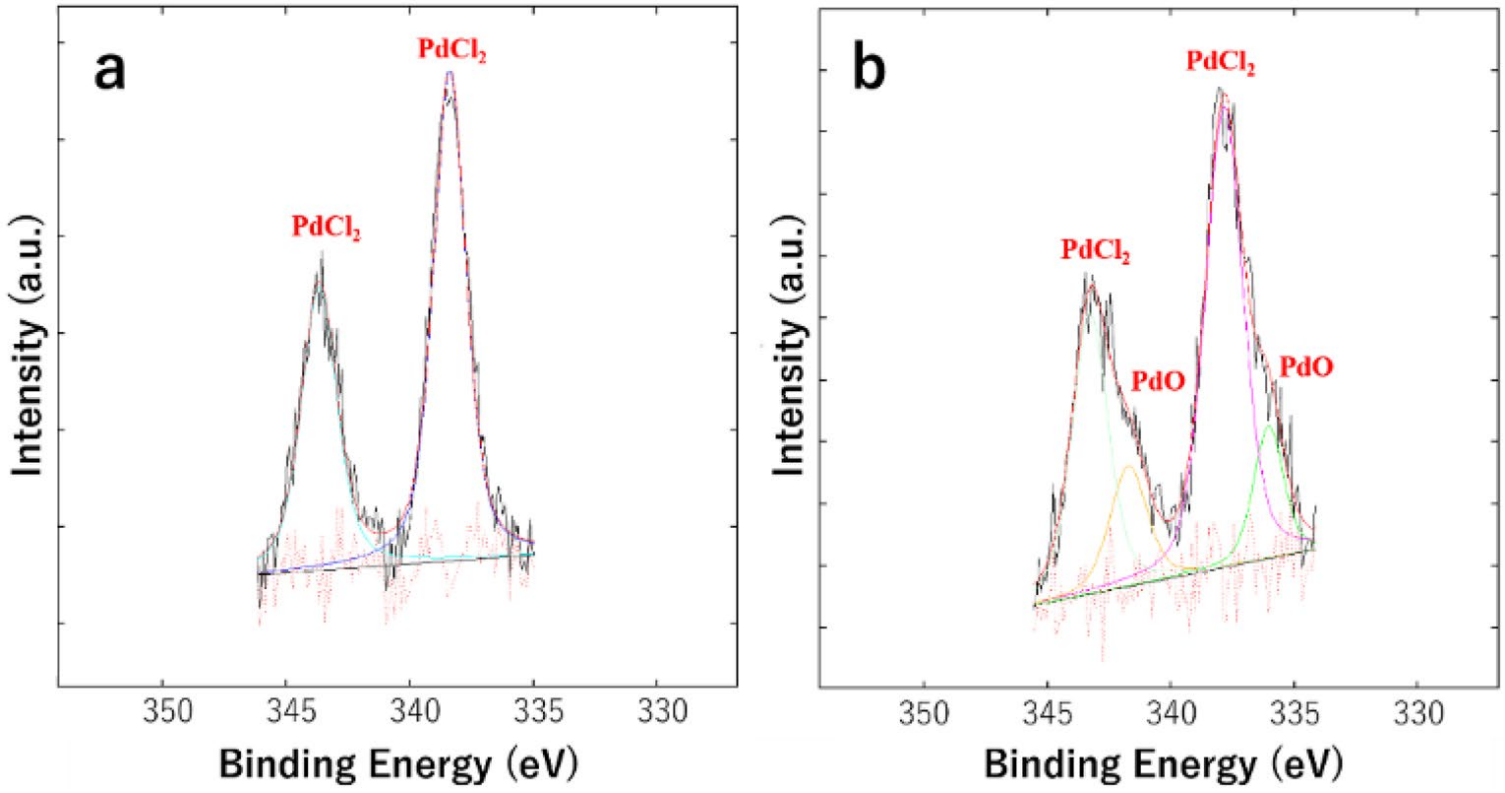
XPS analysis of MNPs@SiO_2_-NH_2_@Pd(dpa)Cl_2_ artificial metalloenzyme before (**a**), and after (**b**), reaction cycles.

The X-ray photoelectron spectroscopy (XPS) of the fresh and recycled MNPs@SiO_2_-NH_2_@Pd(dpa)Cl_2_ artificial metalloenzyme was investigated in order to verify its action mechanism in the Suzuki–Miyaura cross-coupling reaction (Figure S20). The XPS of the fresh MNPs@SiO_2_-NH_2_@Pd(dpa)Cl_2_ artificial metalloenzyme indicated PdCl_2_ species (Fig. 4a); however, after reaction cycles, small shoulder peak in addition to main peak was observed which is assigned to PdO (Fig. 4b). It could be interpreted that the nitrogen-based ligands take a hydrogen ion from a water molecule in reaction media to produce hydroxide ions. Then the chloride ions in PdCl_2_ species could slowly replace with hydroxide ions and produce PdO after the removal of a molecule of water. It should be notice that the XPS spectra of fresh and recycled catalyst did not show any Pd(0) signals, which have been observed simply in reactions based on Pd(0)/Pd(II) mechanisms after catalytic cycles^18, 57^. It could be a good evidence to confirm the Pd(II)/Pd(IV) mechanism^14, 18^ in our designed artificial metalloenzyme-based Suzuki–Miyaura cross-coupling reaction.

On the other hand, in SEM–EDS spectra of MNPs@SiO_2_-NH_2_@Pd(dpa)Cl_2_ nanoparticles (Figure S18), we can see Pd and Cl elements at the same position. The XPS peak was observed at 338.39 eV, being assigned to PdCl_2_ (Fig. 4a). This assignment is supported by SEM–EDS result.

**Figure 5:**
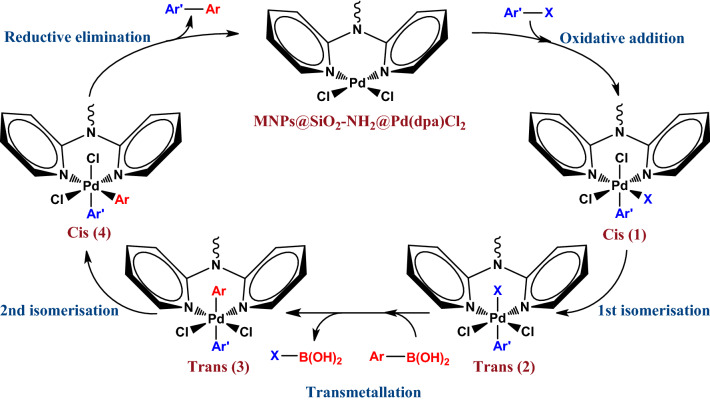
Proposed mechanism of the Suzuki–Miyaura cross-coupling reaction employing MNPs@SiO_2_-NH_2_@Pd(dpa)Cl_2_.

The Suzuki–Miyaura coupling reaction based on Pd(II)/Pd(IV) catalytic cycle involves some fundamental steps as demonstrated in Fig. 5. At the first step *oxidative addition* of aryl halides to Pd(II) complex gives *cis* intermediate (1), a Pd(IV) species. Since the more stable form of the *oxidative addition* product is the *trans* form then, the *cis* product can isomerise to a more stable *trans* intermediate (2). At the *transmetalation* step the organoborane compound reacts with *trans* intermediate (2) to afford *trans* intermediate (3). Then the second isomerization occurs and the *cis* intermediate (4) is produced which is subject to *reductive elimination* to give the desired coupling product and regenerate the original Pd(II) species.

The catalytic activity of the prepared MNPs@SiO_2_-NH_2_@Pd(dpa)Cl_2_ artificial metalloenzyme was evaluated in Suzuki–Miyaura coupling reaction. Amphiphilic structure of MNPs@SiO_2_-NH_2_@Pd(dpa)Cl_2_ could provide a suitable interaction between the water-soluble boronic acids and hydrophobic aryl halides and thereby could improve the efficiency of Suzuki reaction in aqueous media. The dispersibility of magnetic nanoparticles in solvents depends on the suitable choice of their size, precursors, pH, surfactants/coating agents, as well as functionalizing agents.

It has been indicated that the surface charge on colloids, their dispersibility and affinity are well correlated with dielectric constant of the solvents^58^. This is reflected in increase in our examined Suzuki–Miyaura reaction rate employing MNPs@SiO_2_-NH_2_@Pd(dpa)Cl_2_ nanocatalyst in H_2_O:EtOH mixture (1:1) compared with EtOH which is raised from better dispersion of nanocatalyst particles in the reaction media (ESI, Table S1). Water as a “greener” solvent, being more polar, involves in hydrogen bonding. In high polarity index of dispersed medium, a strong electrical double layer forms around the colloidal particles^59, 60^. Then, the zeta potential of nanoparticles increases, and the particles are prevented from aggregation by repelling each other and as a result a good dispersion of nanoparticles could be expected.

The reaction of bromobenzene and phenyl boronic acid was used for initial studies and after optimization of solvent, catalyst amount and temperature (ESI, Table S1) the best result was obtained after 12 min at 60 °C using MNPs@SiO_2_-NH_2_@Pd(dpa)Cl_2_ catalyst (Pd, 0.01 mol%) and K_2_CO_3_ as a base. The presence of organic co-solvent (EtOH) was necessary to ensure the full dissolution of reactants in aqueous medium of dispersed nanocatalyst.

Having optimized the reaction conditions, we examined the scope and limitation of the designed MNPs@SiO_2_-NH_2_@Pd(dpa)Cl_2_ artificial metalloenzyme in the Suzuki–Miyaura cross-coupling reaction of various types of iodo-, bromo-, and chloroaryl derivatives and arylboronic acids (Table 1). Generally, all of the products were obtained in good to excellent yields employing MNPs@SiO_2_-NH_2_@Pd(dpa)Cl_2_ (Pd, 0.01 mol%) and K_2_CO_3_ as a base during the 5–150 min in H_2_O:EtOH (1:1) under aerobic conditions. As expected, aryl iodides were rapidly converted to the respective products even without any base (Table 1, entries 1, 5, 10, 13 and 17). Moreover, bromoaryl derivatives were reacted with boronic acids in high yields, whilst K_2_CO_3_ is necessary for the reactions’ progress. It is worth mentioning that the electron-rich aryl halides, which are known as highly challenging coupling partners, selectively produced the corresponding products and no self-coupling of aryl boronic acids were detected.

**Table 1:** Suzuki–Miyaura coupling of aryl halides with aryl boronic acids employing MNPs@SiO_2_-NH_2_@Pd(dpa)Cl_2_ artificial metalloenzyme.


Entry	Ar	Arʹ	X	T (°C)	Time (min)	Yield^a^ (%)
1^c^	Ph	Ph	I	60	16	100
2	Ph	Ph	Br	60	10	100
3	Ph	Ph	Cl	80	150	87
4	Ph	–C_6_H_4_-4-NH_2_	I	80	120	91
5^c^	3-NO_2_-C_6_H_4_	Ph	I	60	24	92
6	3-NO_2_-C_6_H_4_	Ph	Br	60	90	68
7	Ph	–C_6_H_4_-4-NO_2_	Br	60	5	100
8	Ph	–C_6_H_4_-4-CH_2_OH	Br	80	120	60
9	Ph	–C_6_H_4_-4-CH_2_OH	Cl	80	120	42
10^c^	1-naphthyl	Ph	I	60	22	97
11	1-naphthyl	Ph	Br	60	30	96
12	1-naphthyl	4-Tol	Br	60	20	87
13^c^	2-naphthyl	Ph	I	60	12	99
14	2-naphthyl	Ph	Br	60	15	99
15	2-naphthyl	4-Tol	Br	60	12	98
16	9-anthracenyl	4-Tol	Br	60	20	86
17^b^	9-phenanthryl	Ph	I	60	20	97
18	9-phenanthryl	Ph	Br	60	20	94

Reaction conditions: aryl halide (1.0 mmol), arylboronic acid (1.2 mmol), K_2_CO_3_ (1.5 mmol), MNPs@SiO_2_-NH_2_@Pd(dpa)Cl_2_ (Pd, 0.01 mol% with respect to aryl halide), H_2_O/EtOH (1:1) (2.0 mL).

^a^Isolated yield.

^b^The reaction was performed without any base.

Encouraged by these results, we focused our investigation on the use of more challenging chloroarenes for this reaction employing MNPs@SiO_2_-NH_2_@Pd(dpa)Cl_2_, which could afford the corresponding products at longer reaction times (Table 1, entries 3 and 9).

As a case study, the turnover frequency for the MNPs@SiO_2_-NH_2_@Pd(dpa)Cl_2_ was calculated according to the time-dependent ^1^H NMR spectrum of the reaction mixture of α-naphthaleneboronic acid and 4-bromotoluene under optimized reaction conditions. Accordingly, a high turnover frequency of 1.8 × 10^5^ h^−1^ was observed.

The stability of MNPs@SiO_2_-NH_2_@Pd(dpa)Cl_2_ suspension in water was evaluated by dynamic light scattering (DLS) analysis. The presence of hydrophilic mPEG chains in the surface of MNPs@SiO_2_-NH_2_@Pd(dpa)Cl_2_ provides a stable suspension after its dispersion in water (Figure S15 a).

The DLS analysis indicated that the size and size distribution of MNPs@SiO_2_-NH_2_@Pd(dpa)Cl_2_ nanoparticles suspension remain almost constant even for several days without any precipitation (ESI, Figure S16).

The recyclability of the MNPs@SiO_2_-NH_2_@Pd(dpa)Cl_2_ artificial metalloenzyme was examined upon the Suzuki–Miyaura cross-coupling reaction of iodobenzene and phenylboronic acid under the optimized conditions. The results indicated that the MNPs@SiO_2_-NH_2_@Pd(dpa)Cl_2_ was reused up to 15 cycles without noticeable loss in its catalytic activity (Figure S19 and S21). Moreover, the ICP results before and after catalytic cycles revealed that no appreciable metal leaching was observed in designed MNPs@SiO_2_-NH_2_@Pd(dpa)Cl_2_ artificial metalloenzyme (ICP-MS: 1.05% Pd before and 1.01% Pd after catalytic cycles). This result confirms the robust structure of MNPs@SiO_2_-NH_2_@Pd(dpa)Cl_2_ artificial metalloenzyme after repeated cycles under aerobic conditions in aqueous media.

## Methods

### Synthesis of the [Pd(dpa)Cl_2_] complex^61^

A solution of 1.00 g (5.64 mmol) PdCl_2_ in 57 ml distilled water was added to a solution of 0.968 g (5.65 mmol) 2,2′-dipyridylamine (dpa) in 58 ml of distilled water. The mixture was stirred at 60 °C for 24 h. The resulting brown precipitate was filtered off, and washed three times with 20 ml cold water and dried under vacuum at room temperature. The yield was 70%.

*Pd(dpa)Cl*_*2*_^61^

C_10_H_9_Cl_2_N_3_Pd (MW:348.52): IR (KBr) (cm^−1^): 3416, 2925, 1660, 1604, 1525, 1437, 1032, 770; ^1^H NMR (250.13 MHz, CDCl_3_, 25 °C, TMS): 8.27 (br, 2H), 7.99 (s, 1H), 7.53–7.59 (m, 4H), 6.84 (t, *J* = 5.25, 2H).

### Functionalization of 2,4,6-trichloro-1,3,5-triazine (TCT) with monomethyl ether poly(ethylene glycol) (mPEG)

TCT (550 mg, 3 mmol) and mPEG (2.00 g, 1 mmol) were dissolved in DCM (40 mL). Triethylamine (0.303 g, 3 mmol) was subsequently added to the reaction mixture and it was stirred at room temperature for 16 h. The formed salts were filtrated and the resulting 2-mPEG-4,6-dichloro-1,3,5-triazine was precipitated into cold diethyl ether and vacuum dried^62^.

*2-mPEG-4,6-dichloro-1,3,5-triazine*^62^

IR (KBr) (cm^−1^): 2888, 1617, 1604, 1525, 1467, 1343, 1280, 1119, 963, 842; ^1^H NMR (250.13 MHz, CDCl_3_, 25 °C, TMS): 4.59 (br, 2H), 3.80 (br, 2H), 3.59 (br, 176H), 3.33 (s, 3H).

### [Pd(dpa)Cl_2_] complex conjugation to 2-mPEG-4,6-dichloro-1,3,5-triazine (2-mPEG-4-(Pd(dpa)Cl_2_)-6-chloro-1,3,5-triazine)

[Pd(dpa)Cl_2_] complex (300 mg, 0.86 mmol) was dissolved in dioxan (125 mL) and sodium carbonate (0.99 g, 9.3 mmol) was subsequently added and it was stirred at 30 °C under N_2_ atmosphere. After 5 min a solution of 2-mPEG-4,6-dichloro-1,3,5-triazine (2 g) in dioxan (125 mL) was added dropwise to the reaction mixture under N_2_ atmosphere, then it was stirred for 24 h under the same condition. The resulting 2-mPEG-4-(Pd(dpa)Cl_2_)-6-chloro-1,3,5-triazine was precipitated into cold petroleum ether, and vacuum dried.

### 2-mPEG-4-(Pd(dpa)Cl_2_)-6-chloro-1,3,5-triazine

IR (KBr) (cm^−1^): 3476, 3216, 2888, 2802, 2694, 1701, 1467, 1342, 1280, 1107, 963, 842; ^1^H NMR (250.13 MHz, CDCl_3_, 25 °C, TMS): 8.30 (br, 2H), 7.58–7.63 (m, 4H), 7.01 (t, *J* = 5.25, 2H), 4.41 (br, 2H), 3.82 (br, 2H), 3.56 (br, 176H), 3.30 (s, 3H).

### Preparation of Fe_3_O_4_ magnetite nanoparticles (MNPs)

The co-precipitation approach was employed for the preparation of Fe_3_O_4_ magnetite nanoparticles (MNPs)^63^. Accordingly, FeSO_4_·7H_2_O (0.9 g) and FeCl_3_ (0.98 g) ([Fe^2+^]/[Fe^3+^] = 1:2 molar ratio) were dissolved in 120 mL deionized water at 80 °C under N_2_ atmosphere, then 120 mL of ammonia solution (1.5 M) was added dropwise under vigorous stirring over a period of 20 min. The resulting black MNPs were stirred for another 30 min, then separated under an external magnetic field and repeatedly washed with deionized water and ethanol and dried at 50 °C under vacuum oven (yield: 93%).

### Functionalization of MNPs with tetraethoxysilane (MNPs@SiO_2_)

The MNPs were functionalized with tetraethoxysilane through a modified Stober method^64^. In a typical procedure, prepared Fe_3_O_4_ particles (1.5 g) were dispersed in a mixture of ethanol (105 ml), deionized water (30 ml), and tetraethoxysilane (TEOS) (0.75 ml) in an ultrasonic bath. Afterward, 6 ml of ammonia solution (25%) was added dropwise. After being stirring for 8 h at room temperature, the MNPs@SiO_2_ were collected by magnetic separation and washed with ethanol and deionized water five times and then dried at 60 °C under vacuum oven (yield: 90%).

### Functionalization of MNPs@SiO_2_ with (3-aminopropyl)triethoxysilane (MNPs@SiO_2_-NH_2_)

In order to functionalization of MNPs@SiO_2_ with (3-aminopropyl)triethoxysilane, the synthesized MNPs@SiO_2_ (1.00 g) was dispersed in ethanol (300 mL) via sonication for 15 min. Then (3-aminopropyl)triethoxysilane (4 ml, 8.58 mmol) was added dropwise under mechanical stirring and nitrogen atmosphere at room temperature. Then deionized water (4 ml) was added to increase the hydrolysis rate of (3-aminopropyl)triethoxysilane. The reaction mixture was stirred for 8 h and the obtained amine-functionalized magnetite nanoparticles (MNPs@SiO_2_-NH_2_) was separated by external magnetic field and was washed five times with deionized water and ethanol and dried under vacuum for 24 h at room temperature (yield: 91%).

### Functionalization of MNPs@SiO_2_-NH_2_ with 2-mPEG-4-(Pd(dpa)Cl_2_)-6-chloro-1,3,5-triazine (MNPs@SiO_2_-NH_2_@Pd(dpa)Cl_2_)

In order to functionalization of MNPs@SiO_2_-NH_2_ with 2-mPEG-4-(Pd(dpa)Cl_2_)-6-chloro-1,3,5-triazine, the synthesized MNPs@SiO_2_-NH_2_ (0.85 g) was dispersed in acetonitrile (25 mL) via sonication for 10 min. Then 2-mPEG-4-(Pd(dpa)Cl_2_)-6-chloro-1,3,5-triazine (1.27 g) was added under mechanical stirring and nitrogen atmosphere at 80 °C. The reaction mixture was stirred for 48 h and the obtained MNPs@SiO_2_-NH_2_@Pd(dpa)Cl_2_ was separated by external magnetic field and was washed five times with deionized water and ethanol and dried under vacuum for 24 h at room temperature (yield: 80%).

### General procedure for the Suzuki–Miyaura cross-coupling reaction using MNPs@SiO_2_-NH_2_@Pd(dpa)Cl_2_ artificial enzyme

The Suzuki–Miyaura reaction was performed in a 5 mL round bottomed flask. Accordingly, aryl halide (1.0 mmol), arylboronic acid (1.2 mmol) and K_2_CO_3_ (1.5 mmol) were mixed under optimized reaction conditions [in a dispersed suspension of MNPs@SiO_2_-NH_2_@Pd(dpa)Cl_2_ (Pd, 0.01 mol% with respect to aryl halide) in H_2_O: EtOH (1:1) (2.0 mL) at 60 °C] (ESI, Table S1). The dispersibility of magnetic nanoparticles in solvents depends on the suitable choice of their size, precursors, pH, surfactants/coating agents, as well as functionalizing agents.

It has been indicated that the surface charge on colloids, their dispersibility and affinity are well correlated with dielectric constant of the solvents. This is reflected in increase in our examined Suzuki–Miyaura reaction rate employing MNPs@SiO_2_-NH_2_@Pd(dpa)Cl_2_ nanocatalyst in H_2_O:EtOH mixture (1:1) compared with EtOH which is raised from better dispersion of nanocatalyst particles in the reaction media. Water as a “greener” solvent, being more polar, involves in hydrogen bonding. In high polarity index of dispersed medium, a strong electrical double layer forms around the colloidal particles^17^. Then, the zeta potential of nanoparticles increases, and the particles are prevented from aggregation by repelling each other and as a result a good dispersion of nanoparticles could be expected.

The reaction progress was monitored by TLC. After completion of the reaction, the reaction mixture was allowed to cool to room temperature and was concentrated under reduced pressure. Then the MNPs@SiO_2_-NH_2_@Pd(dpa)Cl_2_ magnetic nanocatalyst was completely separated from the aqueous media using an external magnetic field (Figure S15c). The resulting biaryl was extracted with addition of 2 mL *n*-hexane. The n-hexane phase was dried with MgSO_4_ and the solvent was then removed under reduced pressure to get the product without need to further purification. All the products were characterized by FT-IR and NMR techniques.

The recycling experiments were performed according to the water dispersibility of synthesized MNPs@SiO_2_-NH_2_@Pd(dpa)Cl_2_. The presence of hydrophilic mPEG chains in the surface of MNPs@SiO_2_-NH_2_@Pd(dpa)Cl_2_ provides a means of its complete dispersion into the aqueous phase, and it had no affinity to the *n*-hexane phase. Accordingly, after the first use of the catalyst and vacuo concentration of reaction the product was simply extracted with *n*-hexane while the magnetic nanocatalyst remained in the aqueous phase (Figure S15b). In the next catalytic cycle, ethanol is added to the aqueous phase containing catalyst and it was recharged with reactants and K_2_CO_3_ for the next cycle without any catalyst washing.

## Conclusions

In conclusion, a novel designed robust Pd(II)-based polyfunctional magnetic amphiphilic artificial metalloenzyme was developed. In the designed polyfunctional artificial nanozyme, amphiphilicity originates from the attachment of the mPEG to the lipophilic [Pd(dpa)Cl_2_] complex via cyanuric chloride cross-linker. Moreover, its water dispersibility raised from mPEG chains and the nanomagnetic core as protein mimics endow it other necessary characters of artificial enzymes.

The designed metalloenzyme was efficiently employed for the Suzuki–Miyaura coupling reaction according to an operationally convenient green protocol to afford the desired products in high yields under aerobic conditions in aqueous media. Furthermore, the prepared air and moisture stable artificial metalloenzyme shows excellent reusabilities over at least 15 reaction cycles. Moreover, the artificial metalloenzyme can be easily separated using an external magnetic field without employing inefficient filtration methods. Interestingly, recovery studies indicated that the metal content of recovered MNPs@SiO_2_-NH_2_@Pd(dpa)Cl_2_ did not alter significantly. According to the XPS analysis the Pd(II)/Pd(IV)-based mechanism is the more likely pathway because of the presence of dpa as a strong donor ligand in the designed metalloenzyme that could stabilize organopalladium(IV) complexes in Pd(II)/Pd(IV) mechanism.

## Supplementary Information

Supplementary Information.

## References

[1] Tabushi I (1982). Frontiers of Chemistry.

[2] Breslow R (2009). Biomimetic chemistry: biology as an inspiration. J. Biol. Chem..

[3] Wei H, Wang E (2013). Nanomaterials with enzyme-like characteristics (nanozymes): next-generation artificial enzymes. Chem. Soc. Rev..

[4] Wang Z (2012). Nanoparticle-based artificial RNA silencing machinery for antiviral therapy. Proc. Natl. Acad. Sci..

[5] Fan K (2012). Magnetoferritin nanoparticles for targeting and visualizing tumour tissues. Nat. Nanotechnol..

[6] Rezaei A, Hadian-Dehkordi L, Samadian H (2021). Pseudohomogeneous metallic catalyst based on tungstate-decorated amphiphilic carbon quantum dots for selective oxidative scission of alkenes to aldehyde. Sci. Rep..

[7] Aghahosseini H, Ramazani A, Ślepokura K, Lis T (2018). The first protection-free synthesis of magnetic bifunctional l-proline as a highly active and versatile artificial enzyme: synthesis of imidazole derivatives. J. Colloid Interface Sci..

[8] Aghahosseini H (2018). Highly efficient aqueous synthesis of propargylamines through C–H Activation catalyzed by magnetic organosilica-supported gold nanoparticles as an artificial metalloenzyme. Eur. J. Inorg. Chem..

[9] Aghahosseini H (2019). Pt (II)-based artificial nitroreductase: an efficient and highly stable nanozyme. ChemistrySelect.

[10] Kotov NA (2010). Inorganic nanoparticles as protein mimics. Science.

[11] Rossi LM, Costa NJ, Silva FP, Wojcieszak R (2014). Magnetic nanomaterials in catalysis: advanced catalysts for magnetic separation and beyond. Green Chem..

[12] Sheldon RA, Arends I, Hanefeld U (2007). Green Chemistry and Catalysis.

[13] Christmann U, Vilar R (2005). Monoligated palladium species as catalysts in cross-coupling reactions. Angew. Chem. Int. Ed..

[14] Fiebor A, Tia R, Makhubela BC, Kinfe HH (2018). Water-soluble SNS cationic palladium (II) complexes and their Suzuki-Miyaura cross-coupling reactions in aqueous medium. Beilstein J. Org. Chem..

[15] Morales-Morales D, Redón R, Yung C, Jensen CM (2000). High yield olefination of a wide scope of aryl chlorides catalyzed by the phosphinito palladium PCP pincer complex: [PdCl {C6H3 (OPPri2) 2–2, 6}]. Chem. Commun..

[16] Ohff M, Ohff A, van der Boom ME, Milstein D (1997). Highly active Pd (II) PCP-type catalysts for the Heck reaction. J. Am. Chem. Soc..

[17] Yao Q, Kinney EP, Yang Z (2003). Ligand-free Heck reaction: Pd (OAc) 2 as an active catalyst revisited. J. Org. Chem..

[18] Xu L-M, Li B-J, Yang Z, Shi Z-J (2010). Organopalladium (IV) chemistry. Chem. Soc. Rev..

[19] Byers PK, Canty AJ (1988). Organopalladium (IV) chemistry: oxidative addition of organohalides to dimethylpalladium (II) complexes to form ethyl, σ-benzyl, and σ-allylpalladium (IV) complexes. J. Chem. Soc Chem. Commun..

[20] Byers PK, Canty AJ, Skelton BW, White AH (1986). The oxidative addition of lodomethane to [PdMe 2 (bpy)] and the X-ray structure of the organopalladium (IV) product fac-[PdMe 3 (bpy) l](bpy= 2, 2′-bipyridyl). J. Chem. Soc Chem. Commun..

[21] Canty AJ, Denney MC, van Koten G, Skelton BW, White AH (2004). Carbon−oxygen bond formation at metal (IV) centers: reactivity of palladium (II) and platinum (II) complexes of the [2, 6-(Dimethylaminomethyl) phenyl-N, C, N]-(Pincer) Ligand toward iodomethane and dibenzoyl peroxide; structural studies of M (II) and M (IV) complexes. Organometallics.

[22] Phan N, Van Der Sluys M, Jones C (2006). On the nature of the catalytic species in palladium catalyzed Heck and Suzuki couplings: homogeneous or heterogeneous catalysis, a critical review. Adv. Synth. Cat.

[23] Heck RF (2004). Palladium-catalyzed vinylation of organic halides. Org. React..

[24] Amatore C, Jutand A, Suarez A (1993). Intimate mechanism of oxidative addition to zerovalent palladium complexes in the presence of halide ions and its relevance to the mechanism of palladium-catalyzed nucleophilic substitutions. J. Am. Chem. Soc..

[25] Gonzalez-Sebastian L, Morales-Morales D (2019). Cross-coupling reactions catalysed by palladium pincer complexes. A review of recent advances. J. Organomet. Chem..

[26] Sehnal P, Taylor RJ, Fairlamb IJ (2010). Emergence of palladium (IV) chemistry in synthesis and catalysis. Chem. Rev..

[27] Furuya T, Ritter T (2008). Carbon−fluorine reductive elimination from a high-valent palladium fluoride. J. Am. Chem. Soc..

[28] Wang S, Bruneau C, Renaud J-L, Fischmeister C (2019). 2, 2'-Dipyridylamines: more than just sister members of the Bipyridine family. Applications and achievements in homogeneous catalysis and photoluminescent materials. Dalton Trans..

[29] Breslow R, Zhang X, Huang Y (1997). Selective catalytic hydroxylation of a steroid by an artificial cytochrome P-450 enzyme. J. Am. Chem. Soc..

[30] Dzik WI, Xu X, Zhang XP, Reek JN, de Bruin B (2010). ‘Carbene radicals’ in CoII (por)-catalyzed olefin cyclopropanation. J. Am. Chem. Soc..

[31] Šmejkal T, Breit B (2008). A supramolecular catalyst for regioselective hydroformylation of unsaturated carboxylic acids. Angew. Chem. Int. Ed..

[32] Dydio P, Dzik WI, Lutz M, de Bruin B, Reek JN (2011). Remote supramolecular control of catalyst selectivity in the hydroformylation of alkenes. Angew. Chem. Int. Ed..

[33] Yin L, Liebscher J (2007). Carbon−carbon coupling reactions catalyzed by heterogeneous palladium catalysts. Chem. Rev..

[34] Meijere AD, Diederich F (2004). Metal-catalyzed cross-coupling reactions.

[35] Corbet J-P, Mignani G (2006). Selected patented cross-coupling reaction technologies. Chem. Rev..

[36] Yang Q (2018). Evaluation of potential safety hazards associated with the Suzuki-Miyaura cross-coupling of aryl bromides with vinylboron species. Org. Process. Res. Dev..

[37] Grasa GA (2002). Suzuki−Miyaura cross-coupling reactions mediated by palladium/imidazolium salt systems. Organometallics.

[38] Li J-H, Zhu Q-M, Xie Y-X (2006). Pd (OAc) 2/DABCO-catalyzed Suzuki-Miyaura cross-coupling reaction in DMF. Tetrahedron.

[39] Mutoh Y, Yamamoto K, Saito S (2019). Suzuki-Miyaura cross-coupling of 1, 8-diaminonaphthalene (dan)-protected arylboronic acids. ACS Catal..

[40] Revell JD, Ganesan A (2002). Ionic liquid acceleration of solid-phase Suzuki−Miyaura cross-coupling reactions. Org. Lett..

[41] Woo H, Lee K, Park JC, Park KH (2014). Facile synthesis of Pd/Fe 3 O 4/charcoal bifunctional catalysts with high metal loading for high product yields in Suzuki-Miyaura coupling reactions. New J. Chem..

[42] Key RJ, Tengco JMM, Smith MD, Vannucci AK (2019). A Molecular/Heterogeneous Nickel Catalyst for Suzuki-Miyaura Coupling. Organometallics.

[43] Tamami B, Farjadian F, Ghasemi S, Allahyari H, Mirzadeh M (2015). Palladium nanoparticles supported on poly (N-vinylpyrrolidone)-grafted silica as an efficient catalyst for copper-free sonogashira and suzuki cross-coupling reactions. J. Braz. Chem. Soc..

[44] Yamada YM, Takeda K, Takahashi H, Ikegami S (2003). Highly Active catalyst for the heterogeneous Suzuki− Miyaura reaction: assembled complex of palladium and non-cross-linked amphiphilic polymer. J. Org. Chem..

[45] Yamada YM, Takeda K, Takahashi H, Ikegami S (2002). An assembled complex of palladium and non-cross-linked amphiphilic polymer: a highly active and recyclable catalyst for the Suzuki−Miyaura reaction. Org. Lett..

[46] Lei Y, Zhu W, Wan Y, Wang R, Liu H (2020). Pd nanoparticles supported on amphiphilic porous organic polymer as an efficient catalyst for aqueous hydrodechlorination and Suzuki-Miyaura coupling reactions. Appl. Organomet. Chem..

[47] Schönfelder D, Nuyken O, Weberskirch R (2005). Heck and Suzuki coupling reactions in water using poly (2-oxazoline) s functionalized with palladium carbene complexes as soluble, amphiphilic polymer supports. J. Organomet. Chem..

[48] Wang M, Xue H, Ju F, Yang H (2017). Acceleration of batch-type heterogeneous ligand-free Suzuki-Miyaura reactions with polymer composite supported Pd catalyst. Sci. Rep..

[49] Karimi B, Mansouri F, Vali H (2014). A highly water-dispersible/magnetically separable palladium catalyst based on a Fe3O4@SiO2 anchored TEG-imidazolium ionic liquid for the Suzuki-Miyaura coupling reaction in water. Green Chem..

[50] Karimi B, Zamani A (2012). SBA-15-functionalized palladium complex partially confined with ionic liquid: an efficient and reusable catalyst system for aqueous-phase Suzuki reaction. Org. Biomol. Chem..

[51] Pérez-Lorenzo M (2012). Palladium nanoparticles as efficient catalysts for Suzuki cross-coupling reactions. J. Phys. Chem. Lett..

[52] Chatterjee S, Bhattacharya SK (2018). Size-dependent catalytic activity and fate of palladium nanoparticles in Suzuki-Miyaura coupling reactions. ACS Omega.

[53] Fairlamb IJ, Kapdi AR, Lee AF (2004). η2-dba complexes of Pd (0): the substituent effect in Suzuki−Miyaura coupling. Org. Lett..

[54] Cuenca T, Filice M, Palomo JM (2016). Palladium nanoparticles enzyme aggregate (PANEA) as efficient catalyst for Suzuki-Miyaura reaction in aqueous media. Enzyme Microb. Technol..

[55] Esmaeilpour M, Sardarian AR (2014). Fe_3_O_4_@SiO_2_/Schiff base complex of metal ions as an efficient and recyclable nanocatalyst for the green synthesis of quinoxaline derivatives. Green. Chem. Lett. Rev..

[56] Chattopadhyay S (1997). Raman excitation profiles and molecular structures in the excited electronic states of 2, 2′-dipyridylamine. J. Raman Spectrosc..

[57] Durig J, Layton R, Sink D, Mitchell B (1965). Far infrared spectra of palladium compounds—I. The influence of ligands upon the palladium chloride stretching frequency. Spectrochim. Acta.

[58] Esumi K, Suzuki M, Tano T, Torigoe K, Meguro K (1991). Dispersion of uniformly sized palladium particles in organic solvents. Colloids Surf..

[59] Liu J, Liang C, Zhu X (2016). Understanding the solvent molecules induced spontaneous growth of uncapped tellurium nanoparticles. Sci. Rep..

[60] Tilaki R, Mahdavi S (2007). The effect of liquid environment on size and aggregation of gold nanoparticles prepared by pulsed laser ablation. J. Nanoparticle Res..

[61] Yurdakul Ş, Bilkana M (2015). Spectroscopic and structural properties of 2, 2′-dipyridylamine and its palladium and platinum complexes. Opt. Spectrosc..

[62] Figg CA, Kubo T, Sumerlin BS (2015). Efficient and chemoselective synthesis of ω, ω-heterodifunctional polymers. ACS Macro Lett..

[63] Yuan D, Zhang Q, Dou J (2010). Supported nanosized palladium on superparamagnetic composite microspheres as an efficient catalyst for Heck reaction. Catal. Commun..

[64] Yamada T (2019). Development of titanium dioxide-supported Pd catalysts for ligand-free Suzuki-Miyaura coupling of aryl chlorides. Catalysts.

