# Supplementary material for: A robust polyfunctional Pd(II)-based magnetic amphiphilic nanocatalyst for the Suzuki–Miyaura coupling reaction
Source: Sci Rep. 2021 May 13;11:10239. doi: 10.1038/s41598-021-89424-9 (PMC8119465; doi:10.1038/s41598-021-89424-9)
Supplement: Supplementary file 1 — Supplementary Information. [file 41598_2021_89424_MOESM1_ESM.pdf]

Supporting Information

# A Robust Polyfunctional Pd(II)-Based Magnetic Amphiphilic Nanocatalyst for the Suzuki–Miyaura Coupling Reaction

*Hamideh Aghahosseini,[a,b] Mohammad Reza Saadati,[a] Seyed Jamal Tabatabaei Rezaei,*[a] Ali Ramazani,*[a,b] Narges Asadi,[a] Hidenori Yahiro,[c] Masami Mori,[c] Nahid Shajari[d] and Ali Reza Kazemizadeh[d]*

[a]Department of Chemistry, Faculty of Science, University of Zanjan, Zanjan 45371-38791, Iran, Zanjan, Iran. E-mail: aliramazani@gmail.com; aliramazani@znu.ac.ir; sjt.rezaei@znu.ac.ir

[b]Department of Biotechnology, Research Institute of Modern Biological Techniques (RIMBT), University of Zanjan, Zanjan 45371-38791, Iran.

[c]Department of Materials Science and Biotechnology, Graduate School of Science and Engineering, Ehime University, Matsuyama 790-8577, Japan.

[d]Department of Chemistry, Zanjan Branch, Islamic Azad University, P. O. Box 49195-467, Zanjan, Iran.

## Experimental

### General Information:

All chemicals were sourced from Sigma Aldrich. All solvents were of reagent grade quality and were used without further purification. NMR was obtained on a Bruker 250 MHz spectrometer using $CDCl_3$ as a solvent and TMS as an internal standard. FT-IR spectra were recorded using a Bruker Vector 22 instrument after mixing the samples with KBr. XPS measurements were performed using Perkin-Elmer XPS 1600E with Mg $K_\alpha$ radiation from 400W, 15 kV X-ray source ($hv$ = 1253.6 eV). The absolute binding energies of the photoelectron spectra were determined by referencing to the C 1s transition at 284.50 eV that results most probably during the measurements as adsorbed species. Thermal properties of MNPs@$SiO_2$-$NH_2$ and MNPs@$SiO_2$-$NH_2$@Pd(dpa)$Cl_2$ nanoparticles were analyzed by TGA (SDT Q600 V20.9 Build 20) at the heating rate of 20˚C $min^{-1}$ over the temperature range of 25 to 800˚C. The particle size and morphology of the MNPs@$SiO_2$-$NH_2$@Pd(dpa)$Cl_2$ was investigated by TEM-EDS (Jeol 200 keV field emission transmission electron microscopy) and FE-SEM (MIRA3 TE-SCAN, Brno Czech Republic). For TEM-EDS analysis the sample particles were sonicated in ethanol and 5μl suspension immediately transferred to Cu grids completely covered with carbon, and dried under room conditions. Electron diffraction data were supported by EDS obtained on single nanoparticles. The magnetic properties of the MNPs@$SiO_2$-$NH_2$ and MNPs@$SiO_2$-$NH_2$@Pd(dpa)$Cl_2$ were detected at room temperature using VSM (VSM, Meghnatis Kavir Kashan Co., Kashan, Iran) from −15000 to +15000 Oe. The structural properties of $Fe_3O_4$ and MNPs@$SiO_2$-$NH_2$@Pd(dpa)$Cl_2$ were determined by XRD with a X'Pert-PRO advanced diffractometer using Cu (Ka) radiation (wavelength: 1.5406 Å), operated at 40 kV and 40 MA from 10 to 80˚ at room temperature. The content of palladium in the catalyst was determined using the inductively coupled plasma mass spectroscopy (ICP-MS).

### Synthesis of the [Pd(dpa)$Cl_2$] complex[1]

A solution of 1.00 g (5.64 mmol) $PdCl_2$ in 57 ml distilled water was added to a solution of 0.968 g (5.65 mmol) 2,2′-dipyridylamine (dpa) in 58 ml of distilled water. The mixture was stirred at 60˚C for 24h. The resulting brown precipitate was filtered off, and washed three times with 20 ml cold water and dried under vacuum at room temperature. The yield was 70%.

*Pd(dpa)$Cl_2$*[1]

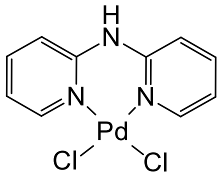

$C_{10}H_9Cl_2N_3Pd$ (MW:348.52): **IR (KBr) ($cm^{-1}$):** 3416, 2925, 1660, 1604, 1525, 1437, 1032, 770; **$^1$H NMR (250.13 MHz, $CDCl_3$, 25°C, TMS):** 8.27 (br, 2H), 7.99 (s, 1H), 7.53-7.59 (m, 4H), 6.84 (t, $J$=5.25, 2H).

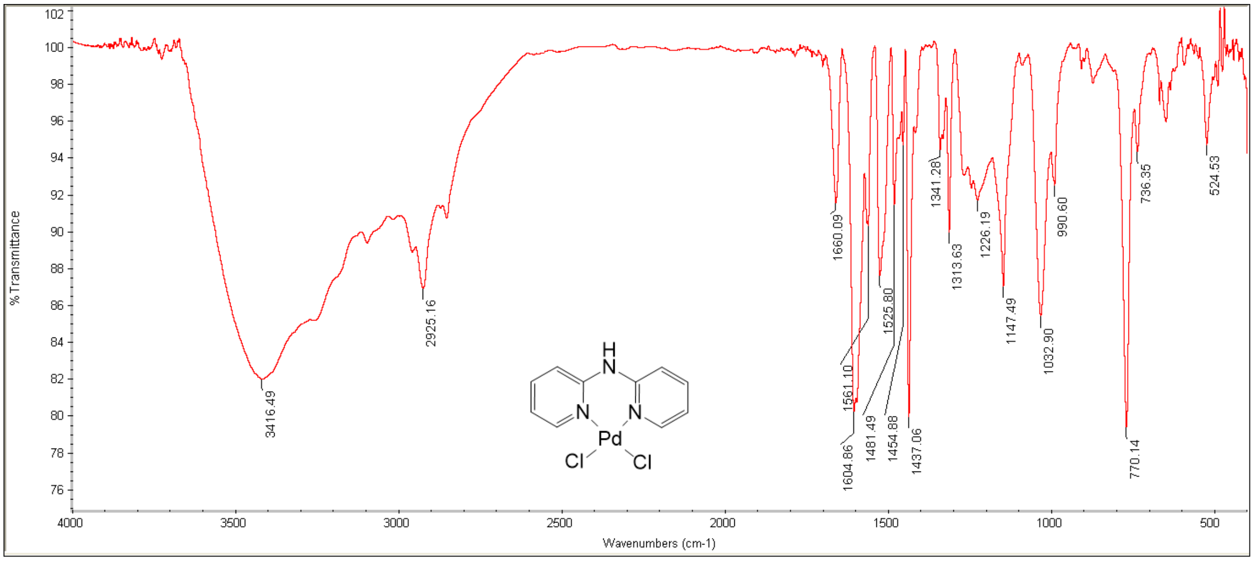


**Figure S1.** FTIR spectrum of [Pt(dpa)$Cl_2$] complex

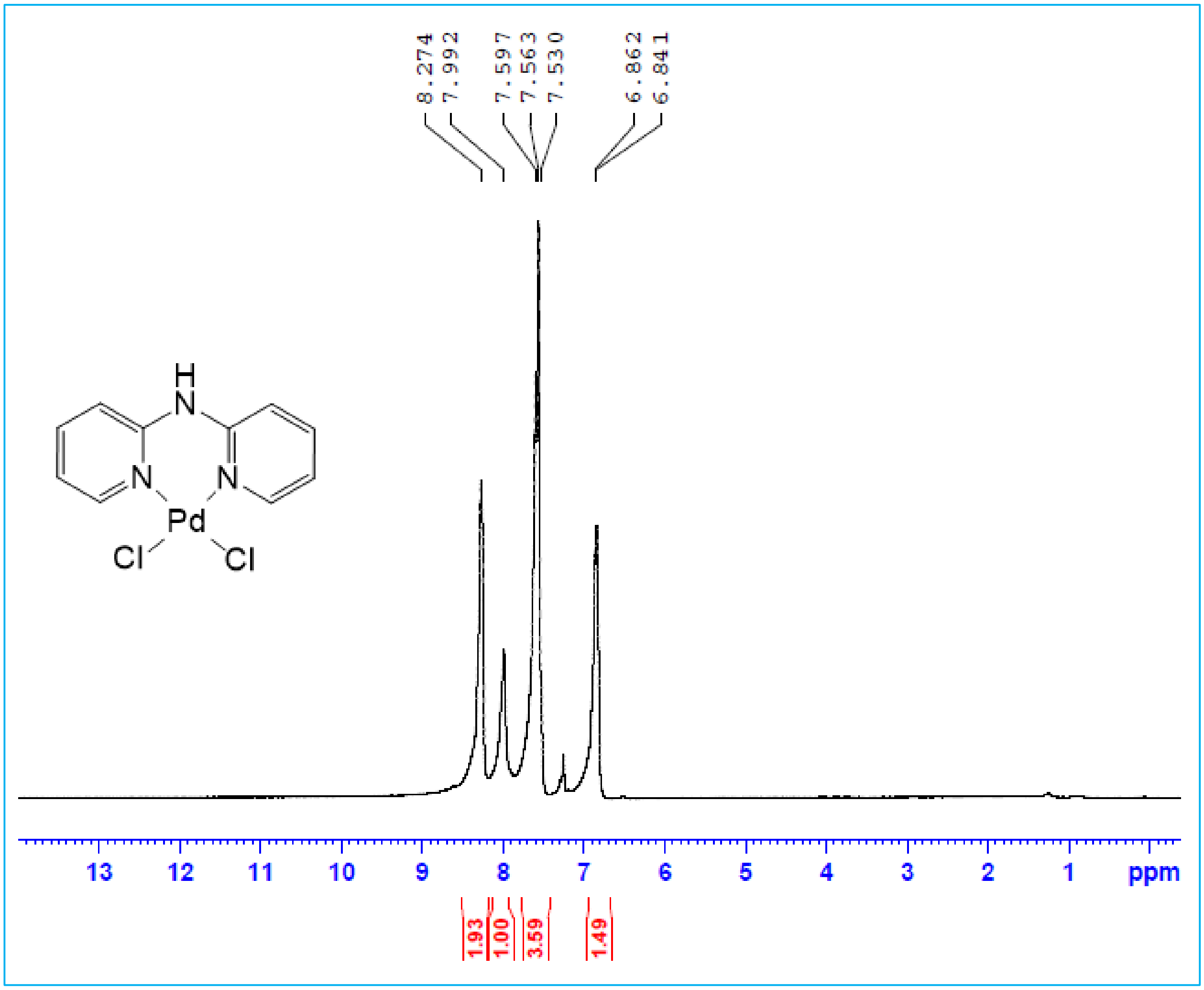


**Figure S2.** $^{1}$H NMR spectrum of [Pt(dpa)$Cl_2$] complex

**Functionalization of 2,4,6-trichloro-1,3,5-triazine (TCT) with monomethyl ether poly(ethylene glycol) (mPEG)**

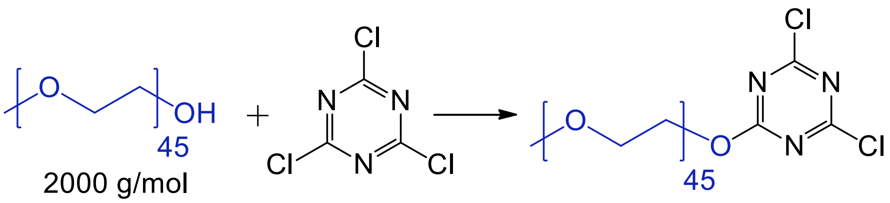


TCT (550 mg, 3 mmol) and mPEG (2.00 g, 1 mmol) were dissolved in DCM (40 mL). Triethylamine (0.303 g, 3 mmol) was subsequently added to the reaction mixture and it was stirred at room temperature for 16 h. The formed salts were filtrated and the resulting 2-mPEG-4,6-dichloro-1,3,5-triazine was precipitated into cold diethyl ether and vacuum dried.[2]

*2-mPEG-4,6-dichloro-1,3,5-triazine*[2]

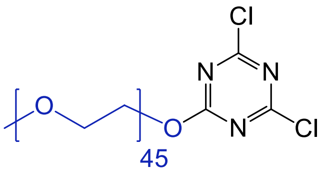


**IR (KBr) ($cm^{-1}$):** 2888, 1617, 1604, 1525, 1467, 1343, 1280, 1119, 963, 842; **$^1$H NMR (250.13 MHz, $CDCl_3$, 25°C, TMS):** 4.59 (br, 2H), 3.80 (br, 2H), 3.59 (br, 176H), 3.33 (s, 3H).

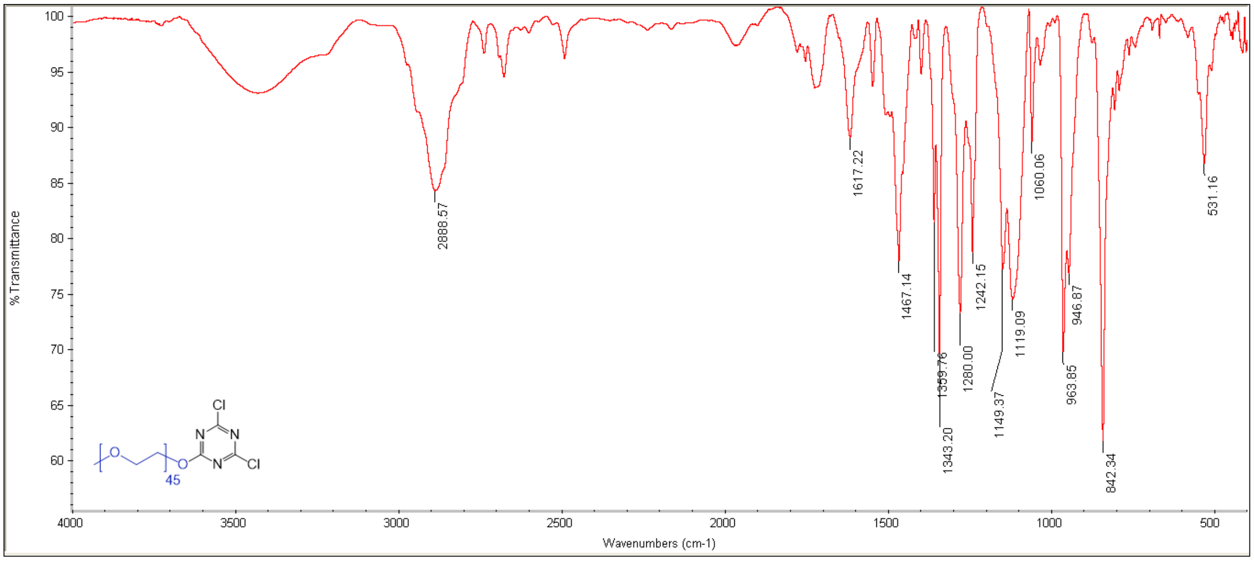


**Figure S3.** FTIR spectrum of 2-mPEG-4,6-dichloro-1,3,5-triazine

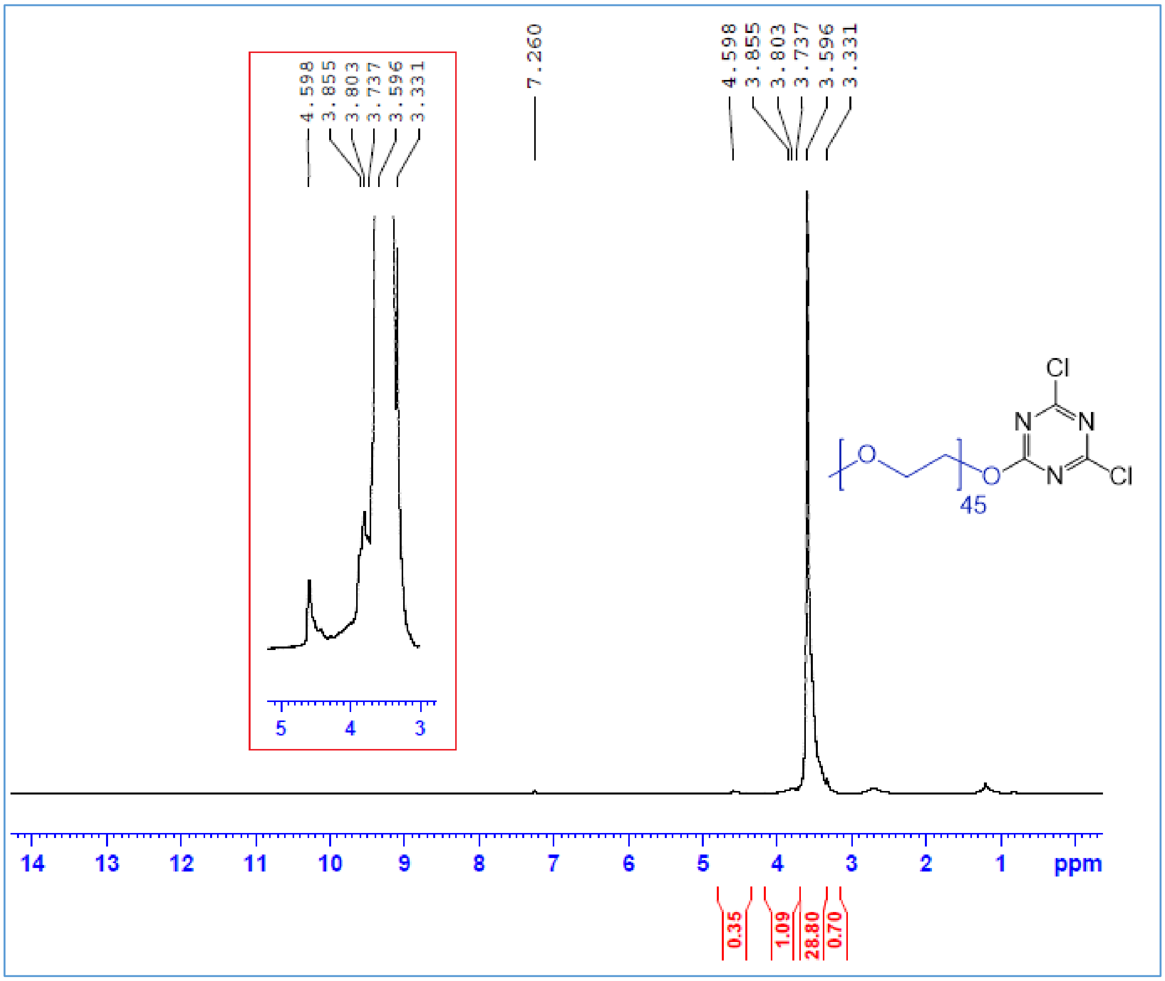


**Figure S4.** $^1$H NMR spectrum of 2-mPEG-4,6-dichloro-1,3,5-triazine

**[Pd(dpa)Cl$_2$] complex conjugation to 2-mPEG-4,6-dichloro-1,3,5-triazine (2-mPEG-4-(Pd(dpa)Cl$_2$)-6-chloro-1,3,5-triazine)**

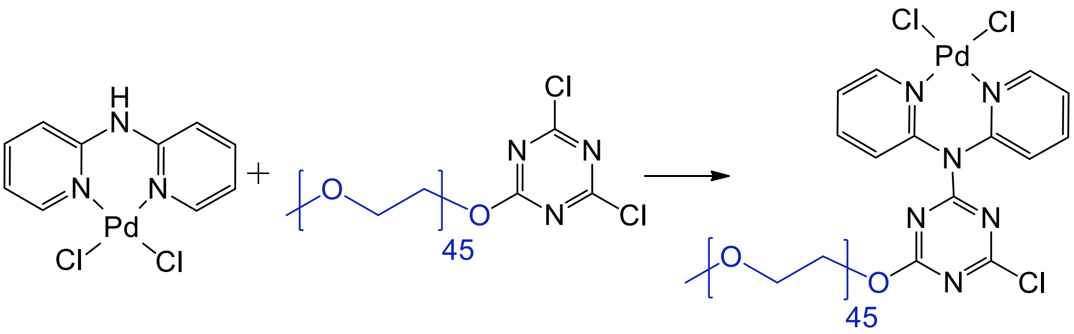


[Pd(dpa)Cl$_2$] complex (300 mg, 0.86 mmol) was dissolved in dioxan (125 mL) and sodium carbonate (0.99 g, 9.3 mmol) was subsequently added and it was stirred at 30°C under $N_2$ atmosphere. After 5 minutes a solution of 2-mPEG-4,6-dichloro-1,3,5-triazine (2 g) in dioxan (125 mL) was added dropwise to the reaction mixture under $N_2$ atmosphere, then it was stirred for 24 h under the same condition. The resulting 2-mPEG-4-(Pd(dpa)Cl$_2$)-6-chloro-1,3,5-triazine was precipitated into cold petroleum ether, and vacuum dried.

*2-mPEG-4-(Pd(dpa)Cl₂)-6-chloro-1,3,5-triazine*

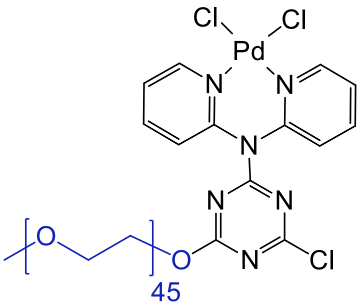

**IR (KBr) ($cm^{-1}$):** 3476, 3216, 2888, 2802, 2694, 1701, 1467, 1342, 1280, 1107, 963, 842; **$^1$H NMR (250.13 MHz, $CDCl_3$, 25°C, TMS):** 8.30 (br, 2H), 7.58-7.63 (m, 4H), 7.01 (t, *J*=5.25, 2H), 4.41 (br, 2H), 3.82 (br, 2H), 3.56 (br, 176H), 3.30 (s, 3H).

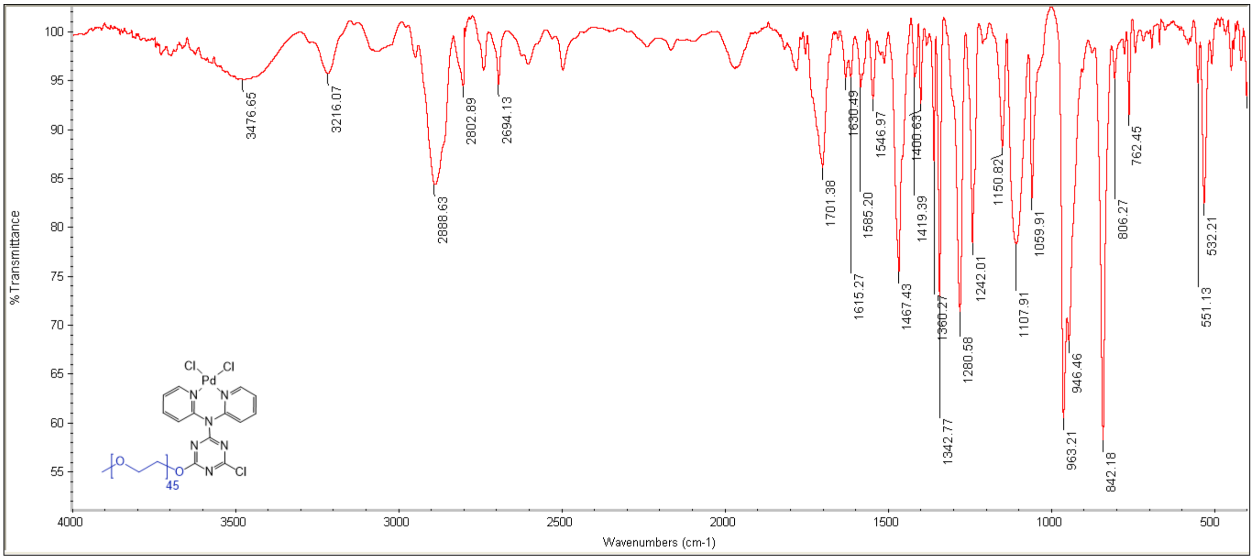


**Figure S5.** FTIR spectrum of 2-mPEG-4-(Pd(dpa)$Cl_2$)-6-chloro-1,3,5-triazine

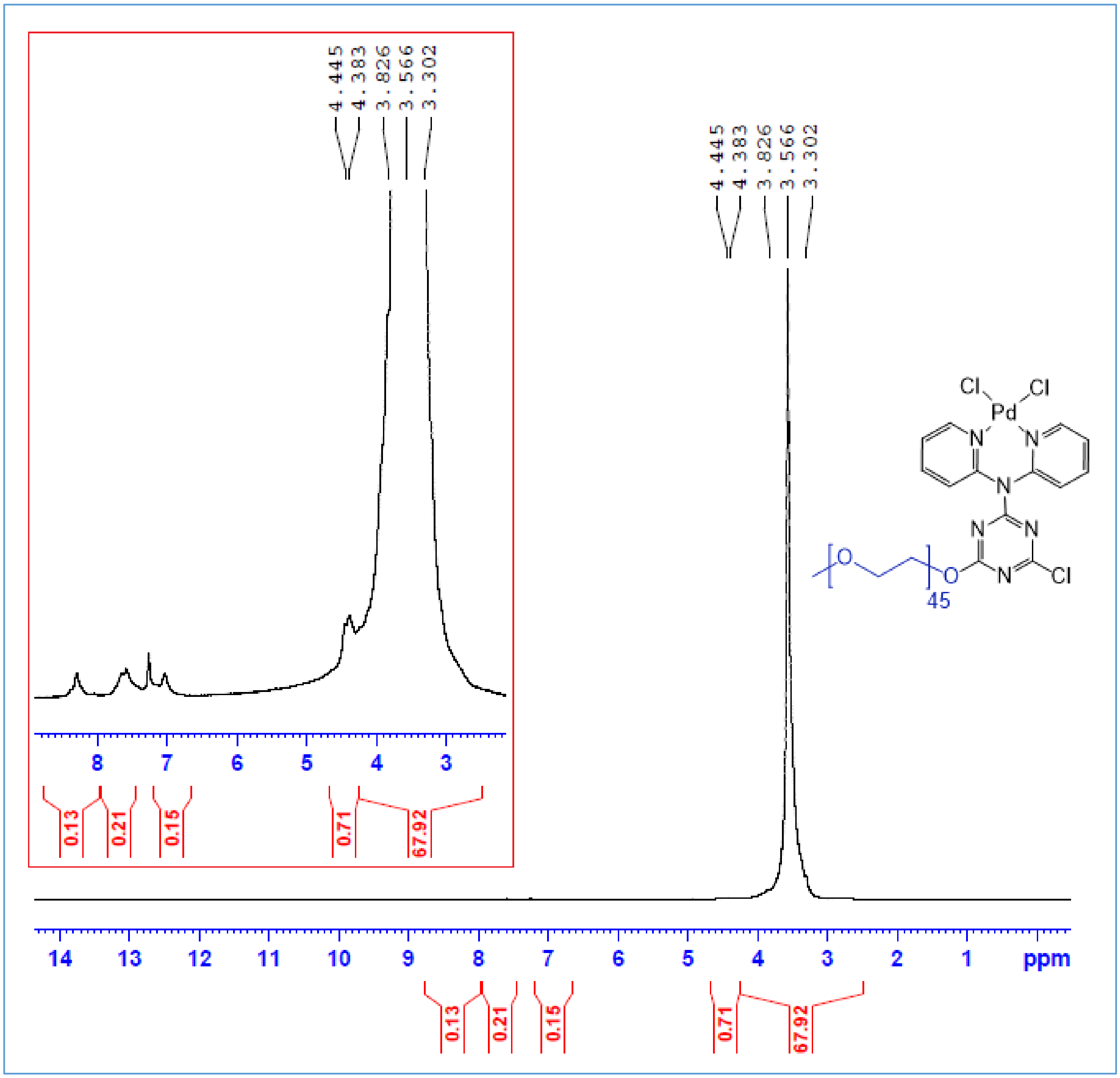


**Figure S6.** $^{1}H$ NMR spectrum of 2-mPEG-4-(Pd(dpa)$Cl_2$)-6-chloro-1,3,5-triazine

**Preparation of $Fe_3O_4$ magnetite nanoparticles (MNPs)**

The co-precipitation approach was employed for the preparation of $Fe_3O_4$ magnetite nanoparticles (MNPs).[3] Accordingly, $FeSO_4.7H_2O$ (0.9 g) and $FeCl_3$ (0.98 g) ([$Fe^{2+}$]/[$Fe^{3+}$]=1 : 2 molar ratio) were dissolved in 120 mL deionized water at 80°C under $N_2$ atmosphere, then 120 mL of ammonia solution (1.5 M) was added dropwise under vigorous stirring over a period of 20 min. The resulting black MNPs were stirred for another 30 min, then separated under an external magnetic field and repeatedly washed with deionized water and ethanol and dried at 50°C under vacuum oven (yield: 93%).

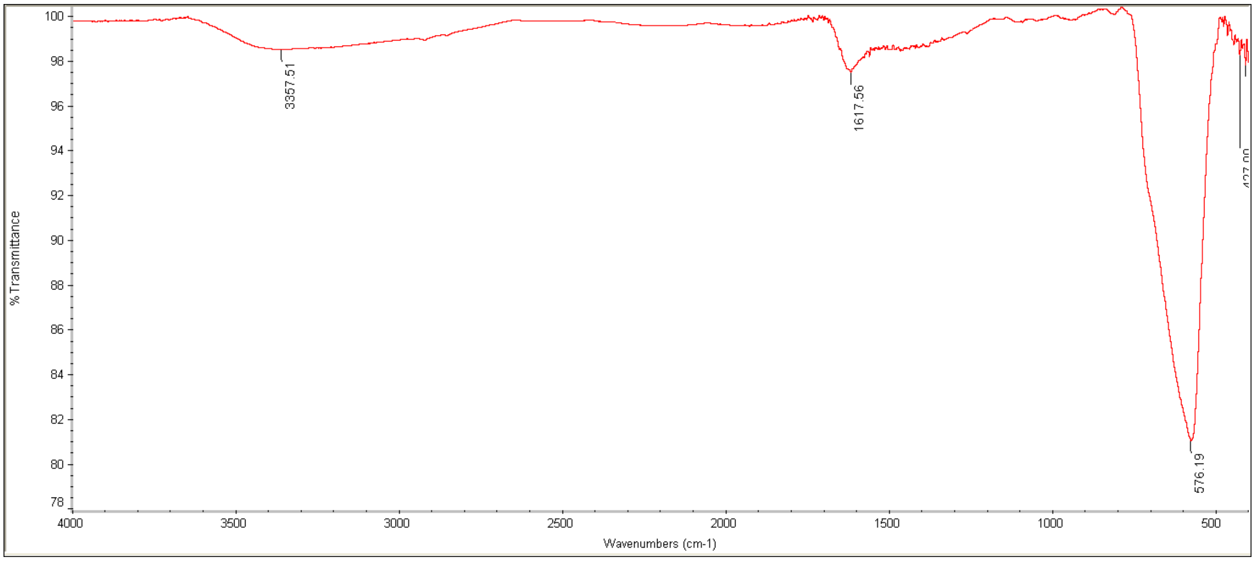


**Figure S7.** FTIR spectrum of $Fe_3O_4$ magnetite nanoparticles (MNPs)

## Functionalization of MNPs with tetraethoxysilane (MNPs@$SiO_2$)

The MNPs were functionalized with tetraethoxysilane through a modified Stober method.[4] In a typical procedure, prepared $Fe_3O_4$ particles (1.5 g) were dispersed in a mixture of ethanol (105ml), deionized water (30 ml), and tetraethoxysilane (TEOS) (0.75 ml) in an ultrasonic bath. Afterward, 6 ml of ammonia solution (25%) was added dropwise. After being stirring for 8 h at room temperature, the MNPs@$SiO_2$ were collected by magnetic separation and washed with ethanol and deionized water five times and then dried at 60 °C under vacuum oven (yield: 90%).

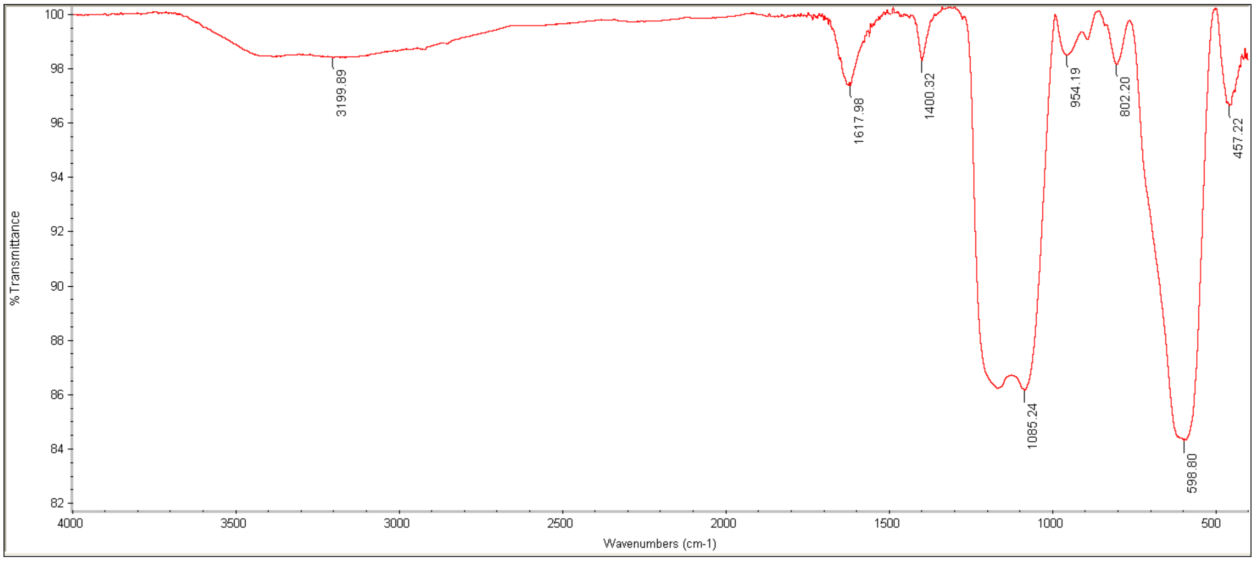


**Figure S8.** FTIR spectrum of MNPs@$SiO_2$

**Functionalization of $MNPs@SiO_2$ with (3-aminopropyl)triethoxysilane ($MNPs@SiO_2-NH_2$)**

In order to functionalization of $MNPs@SiO_2$ with (3-aminopropyl)triethoxysilane, the synthesized $MNPs@SiO_2$ (1.00 g) was dispersed in ethanol (300 mL) *via* sonication for 15 min. Then (3-aminopropyl)triethoxysilane (4 ml, 8.58 mmol) was added dropwise under mechanical stirring and nitrogen atmosphere at room temperature. Then deionized water (4 ml) was added to increase the hydrolysis rate of (3-aminopropyl)triethoxysilane.The reaction mixture was stirred for 8 h and the obtained amine-functionalized magnetite nanoparticles ($MNPs@SiO_2-NH_2$) was separated by external magnetic field and was washed five times with deionized water and ethanol and dried under vacuum for 24 h at room temperature (yield: 91%).

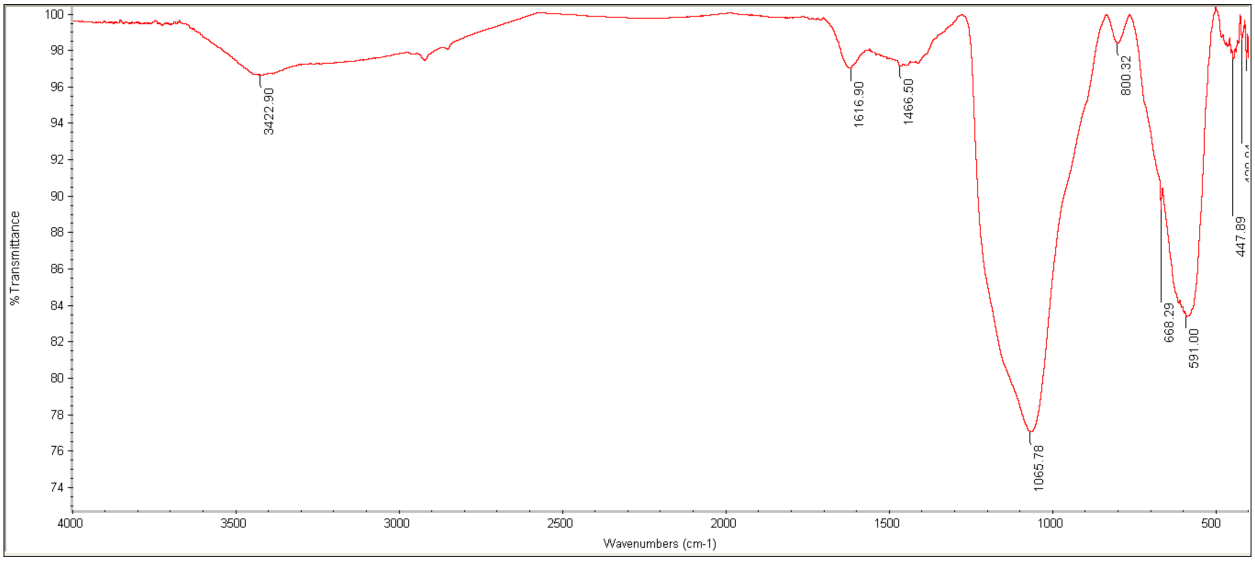


**Figure S9.** FTIR spectrum of $MNPs@SiO_2-NH_2$

**Functionalization of $MNPs@SiO_2-NH_2$ with 2-mPEG-4-(Pd(dpa)$Cl_2$)-6-chloro-1,3,5-triazine ($MNPs@SiO_2-NH_2@Pd(dpa)Cl_2$)**

In order to functionalization of $MNPs@SiO_2-NH_2$ with 2-mPEG-4-(Pd(dpa)$Cl_2$)-6-chloro-1,3,5-triazine, the synthesized $MNPs@SiO_2-NH_2$ (0.85 g) was dispersed in acetonitrile (25 mL) *via* sonication for 10 min. Then 2-mPEG-4-(Pd(dpa)$Cl_2$)-6-chloro-1,3,5-triazine (1.27 g) was added under mechanical stirring and nitrogen atmosphere at 80 ˚C. The reaction mixture was stirred for 48 h and the obtained $MNPs@SiO_2-NH_2@Pd(dpa)Cl_2$ was separated by external magnetic field and was washed five times with deionized water and ethanol and dried under vacuum for 24 h at room temperature (yield: 80%).

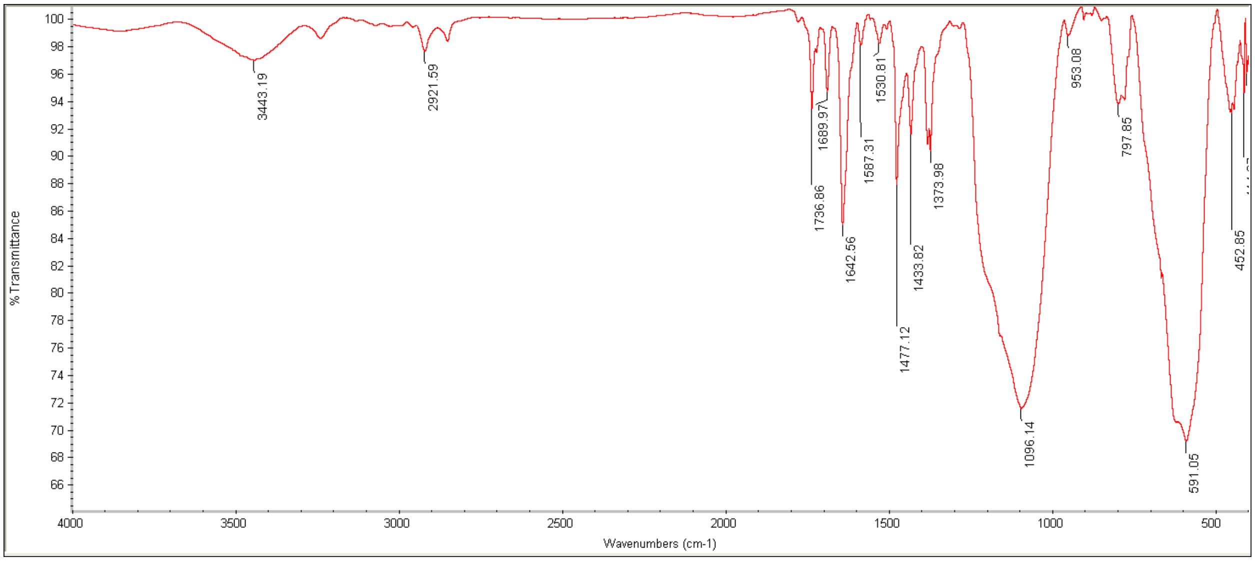


**Figure S10.** FTIR spectrum of MNPs@$SiO_2$-$NH_2$@Pd(dpa)$Cl_2$

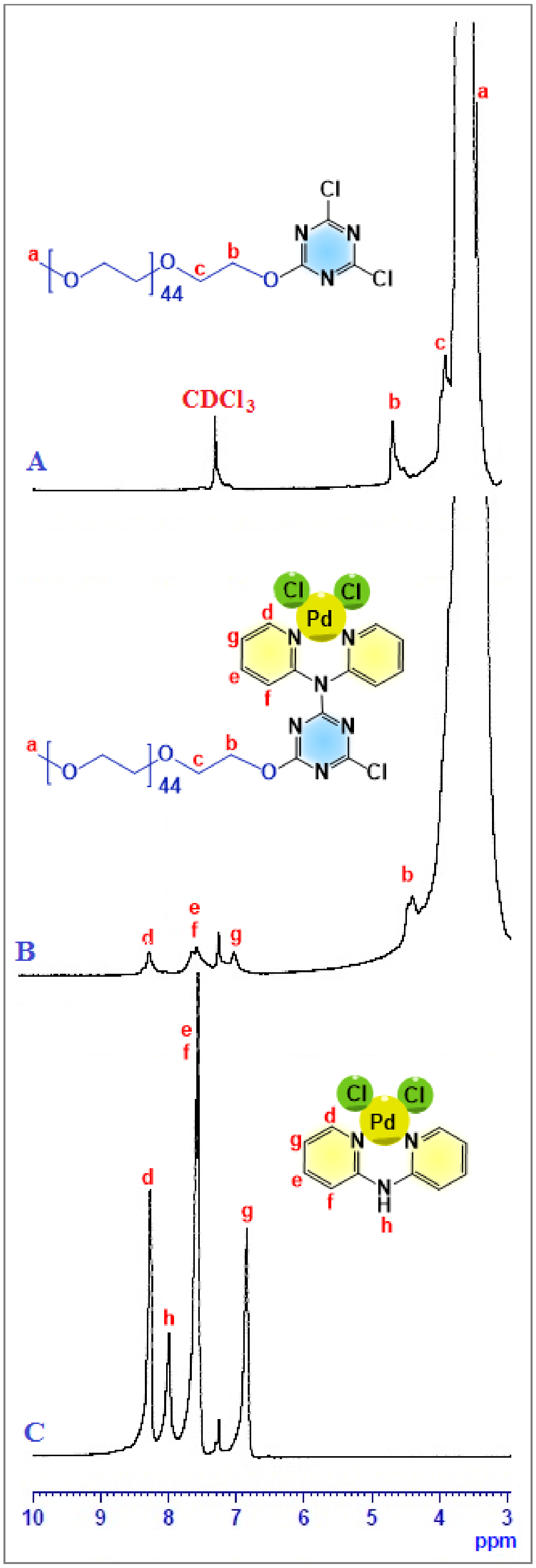


**Figure S11.** $^{1}$H NMR spectrum of (**A**) 2-mPEG-4,6-dichloro-1,3,5-triazine, which confirms successful addition of 2,4,6-trichloro-1,3,5-triazine to mPEG; (**B**) 2-mPEG-4-[Pd(dpa)$Cl_2$]-6-chloro-1,3,5-triazine; (**C**) Pd(dpa)$Cl_2$. Disappearance of NH peak in δ: 7.99 ppm (h) in **B** comparing with **C**, confirm successful addition of Pd(dpa)$Cl_2$ to 2-mPEG-4,6-dichloro-1,3,5-triazine.

**General procedure for the Suzuki–Miyaura cross-coupling reaction using MNPs@SiO$_2$-NH$_2$@Pd(dpa)Cl$_2$ artificial enzyme**

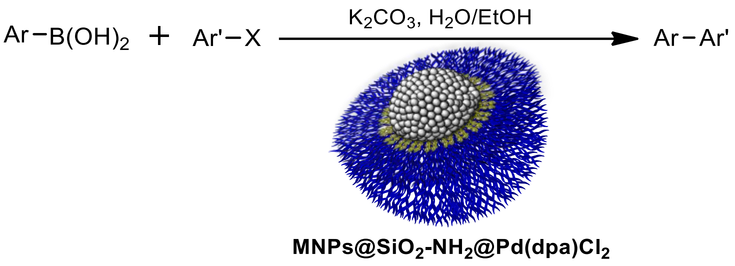


The Suzuki–Miyaura reaction was performed in a 5 mL round bottomed flask. Accordingly, aryl halide (1.0 mmol), arylboronic acid (1.2 mmol) and $K_2CO_3$ (1.5 mmol) were mixed under optimized reaction conditions (in a dispersed suspension of MNPs@SiO$_2$-NH$_2$@Pd(dpa)Cl$_2$ (0.01 mol% with respect to aryl halide) in $H_2O$: EtOH (1:1) (2.0 mL) at 60 °C) (Table S1). The reaction progress was monitored by TLC. After completion of the reaction, the reaction mixture was allowed to cool to room temperature and was concentrated under reduced pressure. Then the MNPs@SiO$_2$-NH$_2$@Pd(dpa)Cl$_2$ magnetic nanocatalyst was completely separated from the aqueous media using an external magnetic field. The resulting biaryl was extracted with addition of 2 mL *n*-hexane. The n-hexane phase was dried with $MgSO_4$ and the solvent was then removed under reduced pressure to get the product without need to further purification. All the products were characterized by FT-IR and NMR techniques.

**Table S1.** Optimization of the Suzuki coupling reaction of bromobenzene and phenyl boronic acid in the presence of MNPs@SiO$_2$-NH$_2$@Pd(dpa)Cl$_2$ and $K_2CO_3$ as a base.

| Entry | Catalyst (mol%) | Solvent | Temp (˚C) | Time (min) | Conv (%) |
|---|---|---|---|---|---|
| 1 | 0.1 | $H_2O$ | 60 | 120 | 0 |
| 2 | 0.1 | EtOH | 60 | 12 | 100 |
| 3 | 0.01 | EtOH | 60 | 40 | 100 |
| 4 | 0.1 | $H_2O$: EtOH (1:1) | 25 | 30 | 10 |
| 5 | 0.1 | $H_2O$: EtOH (1:1) | 60 | 12 | 100 |
| 6 | 0.1 | $H_2O$: EtOH (1:1) | 80 | 12 | 100 |
| **7** | **0.01** | **$H_2O$: EtOH (1:1)** | **60** | **12** | **100** |
| 8 | 0.05 | $H_2O$: EtOH (1:1) | 60 | 12 | 100 |
| 9 | 0.15 | $H_2O$: EtOH (1:1) | 60 | 12 | 100 |
| 10 | 0.2 | $H_2O$: EtOH (1:1) | 60 | 12 | 100 |

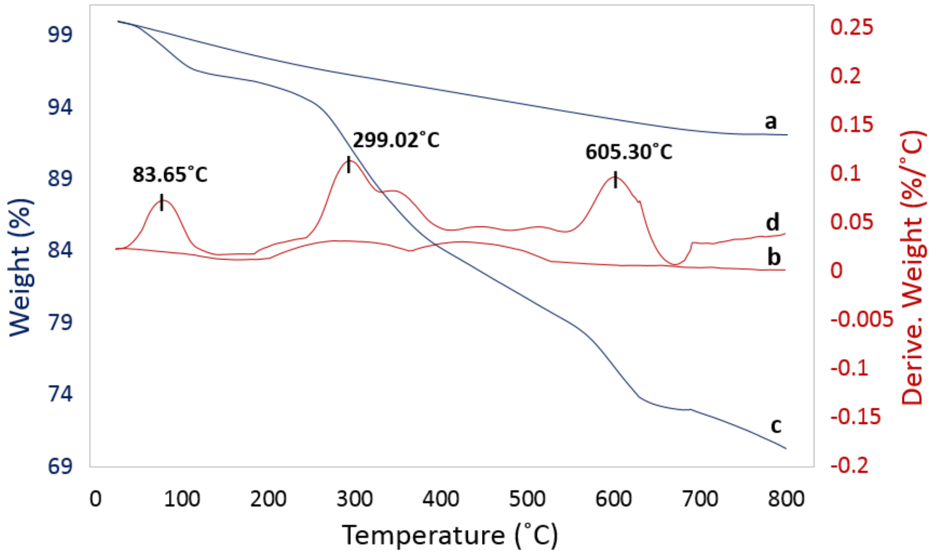


**Figure S12.** TG and DTG curves of $MNPs@SiO_2-NH_2$ (a and b), and $MNPs@SiO_2-NH_2@Pd(dpa)Cl_2$ nanoparticles (c and d).

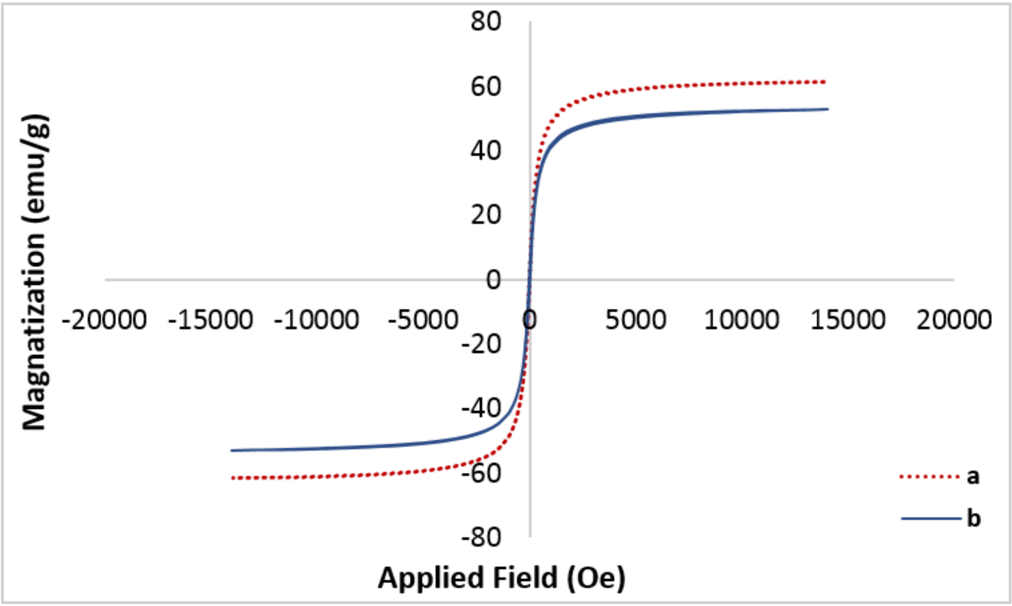


**Figure S13.** Room temperature magnetization curves of $MNPs@SiO_2-NH_2$ (a), and $MNPs@SiO_2-NH_2@Pd(dpa)Cl_2$ nanoparticles (b).

The XRD patterns of $Fe_3O_4$ and MNPs@$SiO_2$-$NH_2$@Pd(dpa)$Cl_2$ nanoparticles are represented in figure S14. The X-ray diffraction (XRD) pattern of prepared MNPs@$SiO_2$-$NH_2$@Pd(dpa)$Cl_2$ represented strong and sharp peaks that can be ascribed to the pure phase of magnetite in its structure (Figure S14). Additionally, the diffraction peaks of nanostructured palladium complex can be observed over the MNPs@$SiO_2$-$NH_2$@Pd(dpa)$Cl_2$ sample. The crystal structure of [Pd(dpa)$Cl_2$] complex or the overall structure of the supporting materials in MNPs@$SiO_2$-$NH_2$@Pd(dpa)$Cl_2$ structure could generate some unmarked peaks in XRD pattern. As it was observed in some reports the crystal structure of supporting materials negligibly influenced by the modification process with Pd(0) or Pd(II)[5]. Therefore, interpretation of a series of anonymous short peaks is complex and requires extensive investigations.

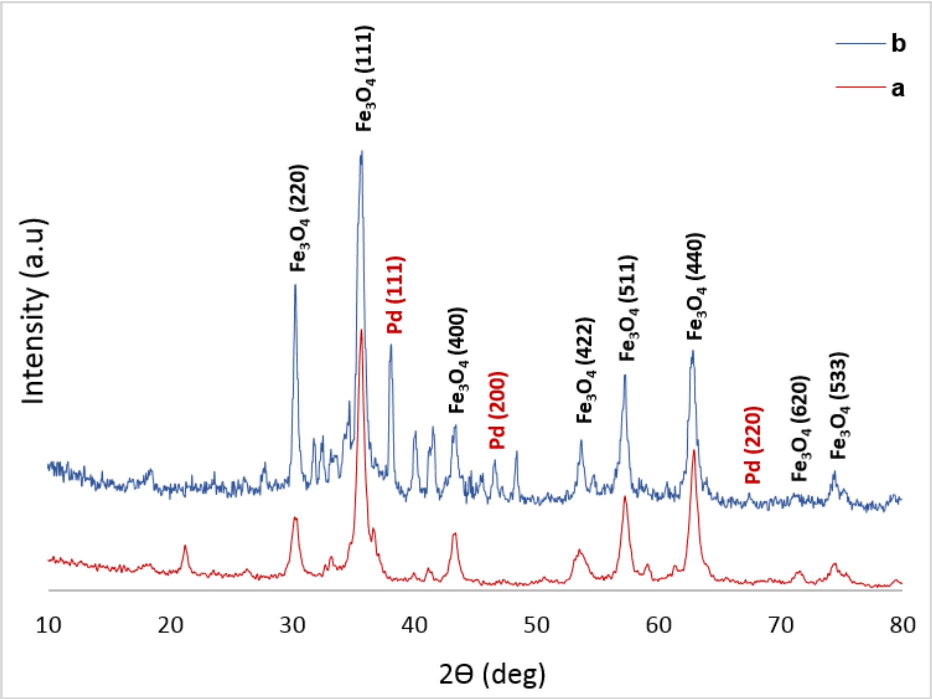


**Figure S14.** XRD patterns of $Fe_3O_4$ (a), and MNPs@$SiO_2$-$NH_2$@Pd(dpa)$Cl_2$ nanoparticles (b).

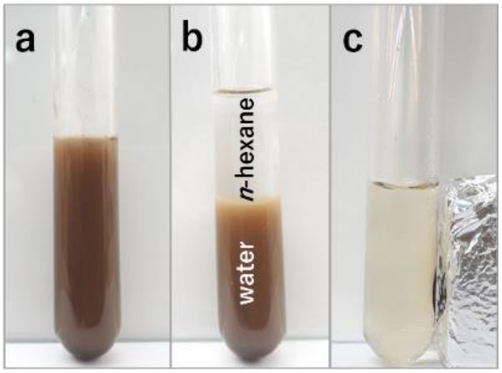


**Figure S15.** Good dispersion of MNPs@$SiO_2$-$NH_2$@Pd(dpa)$Cl_2$ nanocatalyst in water (a), distribution of MNPs@$SiO_2$-$NH_2$@Pd(dpa)$Cl_2$ nanocatalyst in a biphasic water/n-hexane solvents (b), and simple separation of MNPs@$SiO_2$-$NH_2$@Pd(dpa)$Cl_2$ nanocatalyst by an external magnet (c).

The hydrodynamic diameter of MNPs@$SiO_2$-$NH_2$@Pd(dpa)$Cl_2$ in water was measured by DLS measurements (Fig. S16).
We have evaluated the initial hydrodynamic diameter of MNPs@$SiO_2$-$NH_2$@Pd(dpa)$Cl_2$ colloidal solution after its mixing for 5 min before analysis and then after several days that showed well dispersed nanoparticles with a diameter of about 84 nm (Fig. S16).

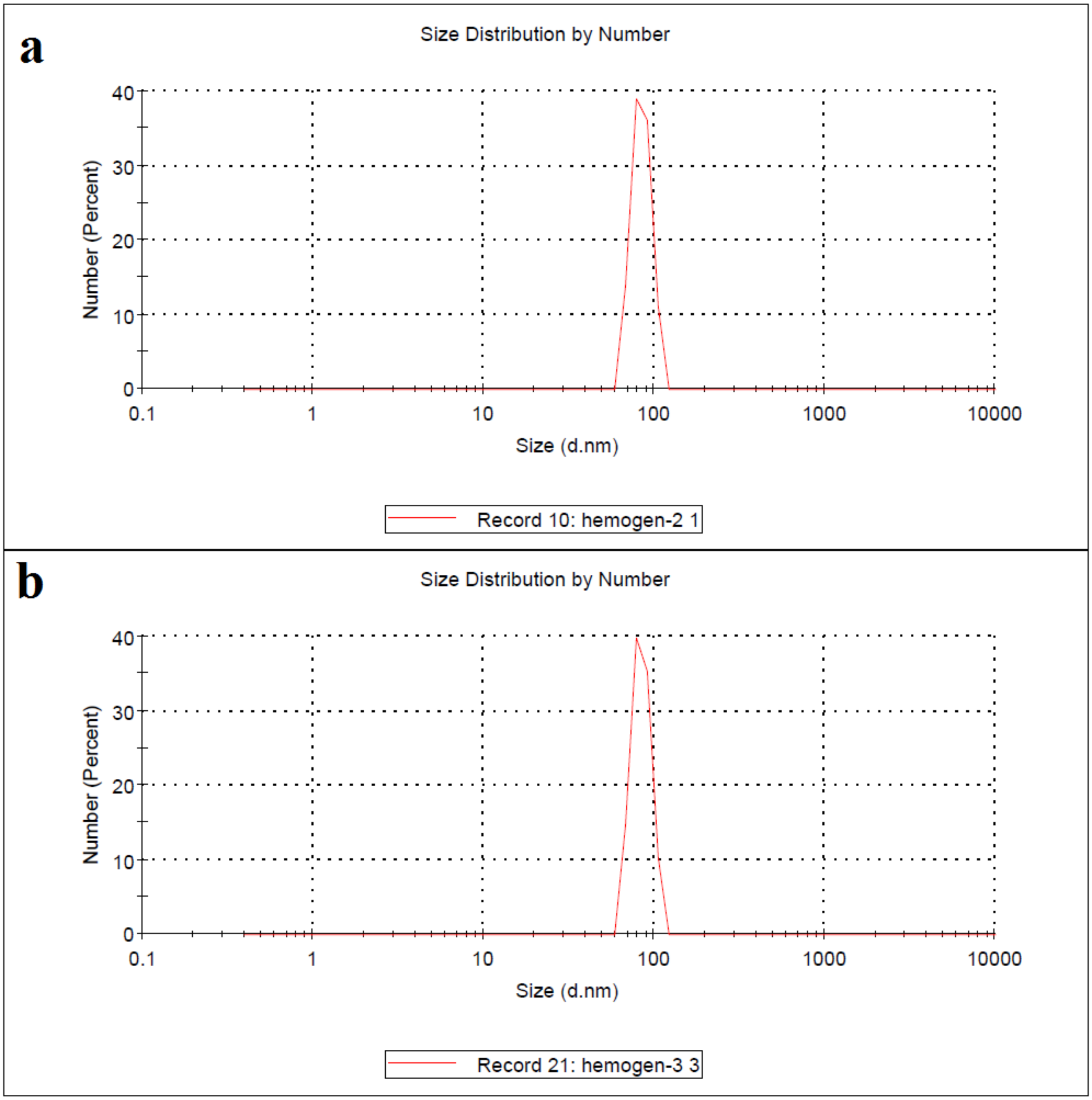


**Figure S16.** DLS size distribution histogram of MNPs@$SiO_2$-$NH_2$@Pd(dpa)$Cl_2$ in pure water after 5 min mixing before analysis (a) and after several days (b).

TEM images of MNPs@$SiO_2$-$NH_2$@Pd(dpa)$Cl_2$ nanoparticles and their particle size histogram are shown in Figure S17. The TEM-EDS images measurement conditions are as follows:

Nanoparticles was sonicated in ethanol and 5μl suspension immediately transferred to Cu grids completely covered with carbon, and dried under room conditions. They were analyzed in a Jeol 200 keV field emission transmission electron microscopy and electron diffraction data were supported by EDS obtained on single nanoparticles.

Moreover, the Table S2 represented the EDX measurement of spots 1 to 5 on TEM photographs (Figure S17), respectively. The black patch of spot-1 consists of large amount of Fe. In addition, we can see Si peak. A small black spot (spots 3 and 4) contains Pd mainly. The Cu peak could be ignored because of the usage of copper grid for TEM measurement.

The oxidation state of Pd couldn't determine by EDX. In principle, EDX can also measure the oxidation state of a compound, but the difference between XPS and EDX comes down to the energy resolution of the detectors in each case. EDX detects X-rays from higher level shell electrons falling into core electron shells after an electron beam has knocked out the core electron. XPS measure the kinetic energy of a core electron that has been knocked out by and X-ray The energy of a core electron is affected by the oxidation state and can vary by a couple of eV, which goes back to my initial statement that EDX and XPS are measuring the same energy. The difference is that EDX detectors have resolutions well above 2 eV, which is a consequence of the technological limits of measuring X-ray energies, whereas measuring electron energies is as simple as passing them through a magnetic prism which (analogous to a light prism) separates electrons smoothly based on their energy, attaining sub eV resolution[6].

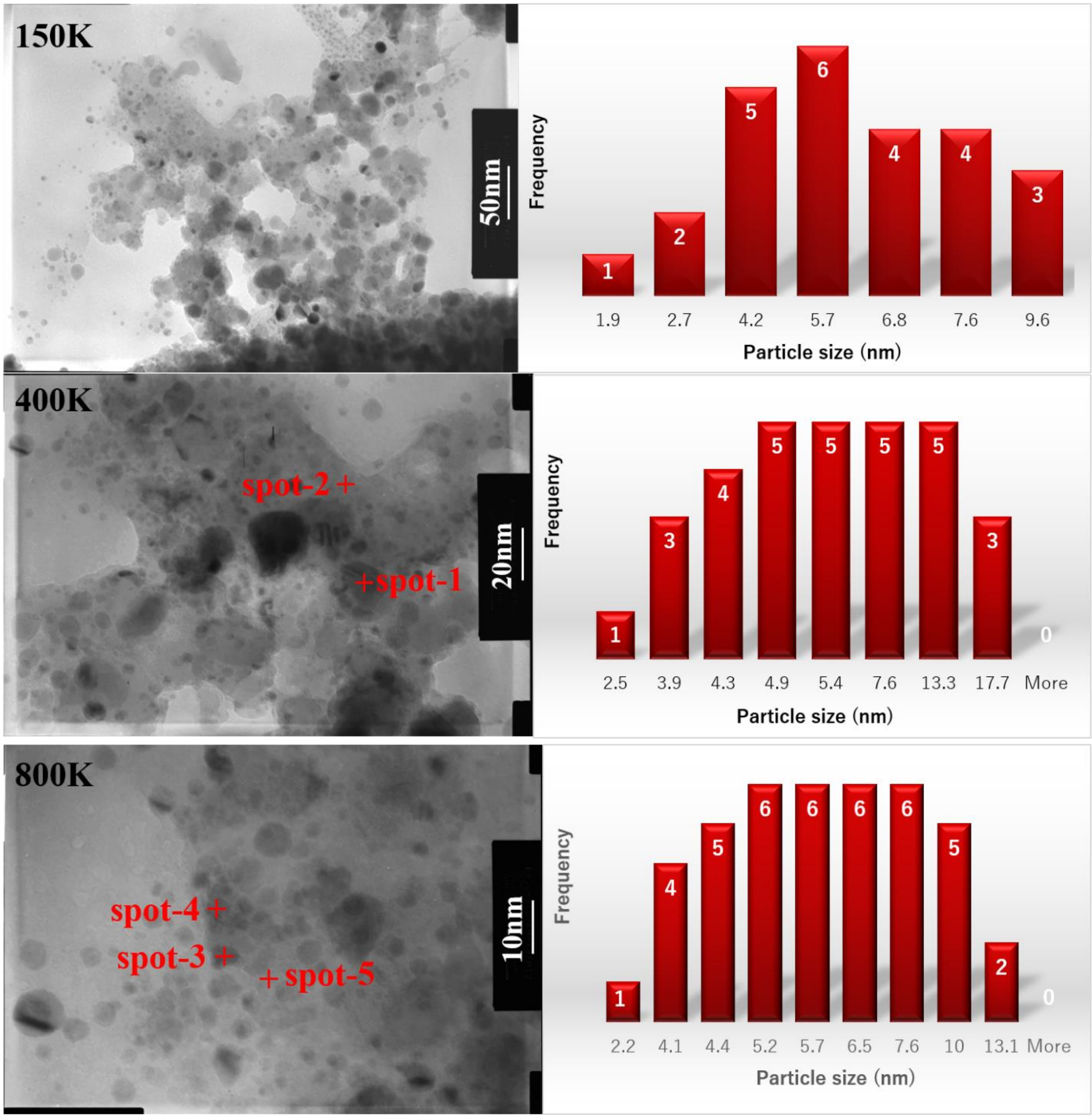


**Figure S17.** FE-TEM spectra with particle size histogram of MNPs@$SiO_2$-$NH_2$@Pd(dpa)$Cl_2$ nanoparticles.

**Table S2.** The EDX measurement (element analysis) of spots 1 to 5 on TEM photographs (Figure S17)

<table>
<tr><th>Entry</th><th>EDS graph of spots 1 to 5 on TEM photographs and their quantitative data table</th></tr>
<tr><td>1</td><td>
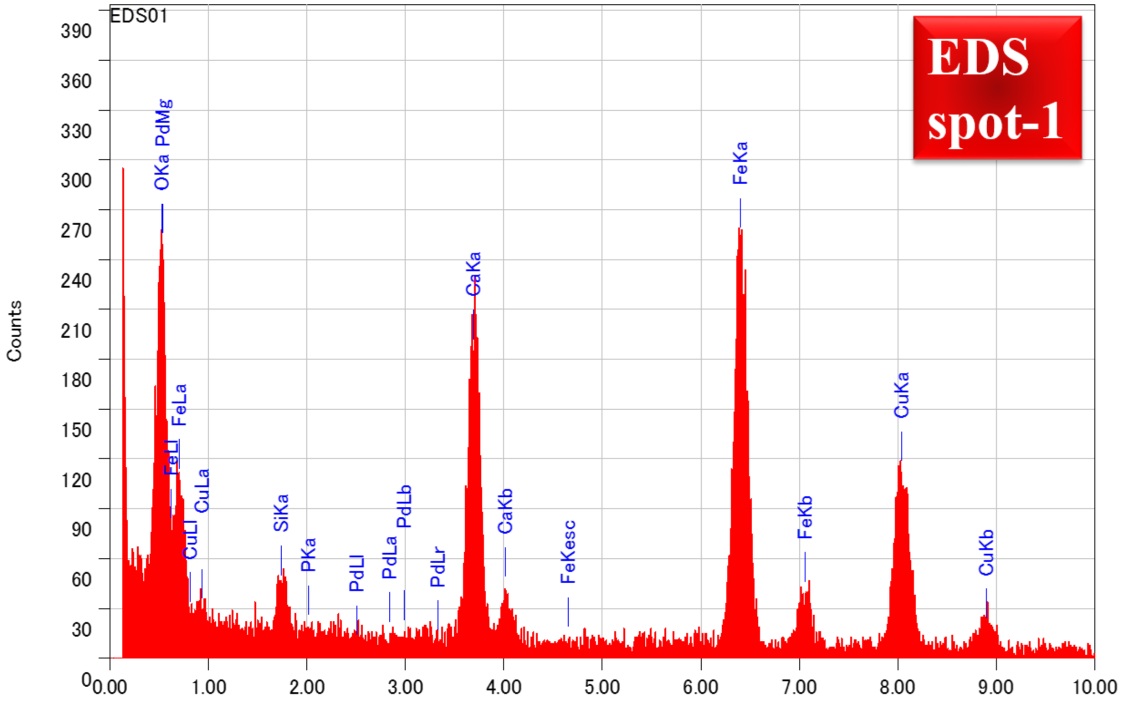


Thin film EDS quantitative analysis
Fitting coefficient : 0.4200

| element | (keV) | mass% | count | error% | atomic% |
|---|---|---|---|---|---|
| Fe K(standard) | 6.398 | 39.42 | 3602.12 | 0.02 | 26.54 |
| Cu K | 8.040 | 23.82 | 1706.81 | 0.04 | 14.09 |
| O K | 0.525 | 17.25 | 1375.99 | 0.02 | 40.53 |
| Ca K | 3.690 | 16.89 | 2100.44 | 0.03 | 15.84 |
| Si K | 1.739 | 1.99 | 288.37 | 0.20 | 2.67 |
| Pd L | 2.838 | 0.51 | 19.26 | 2.75 | 0.18 |
| P K | 2.013 | 0.13 | 17.01 | 2.94 | 0.15 |
| total | | 100.00 | | | 100.00 |

</td></tr>
</table>

2

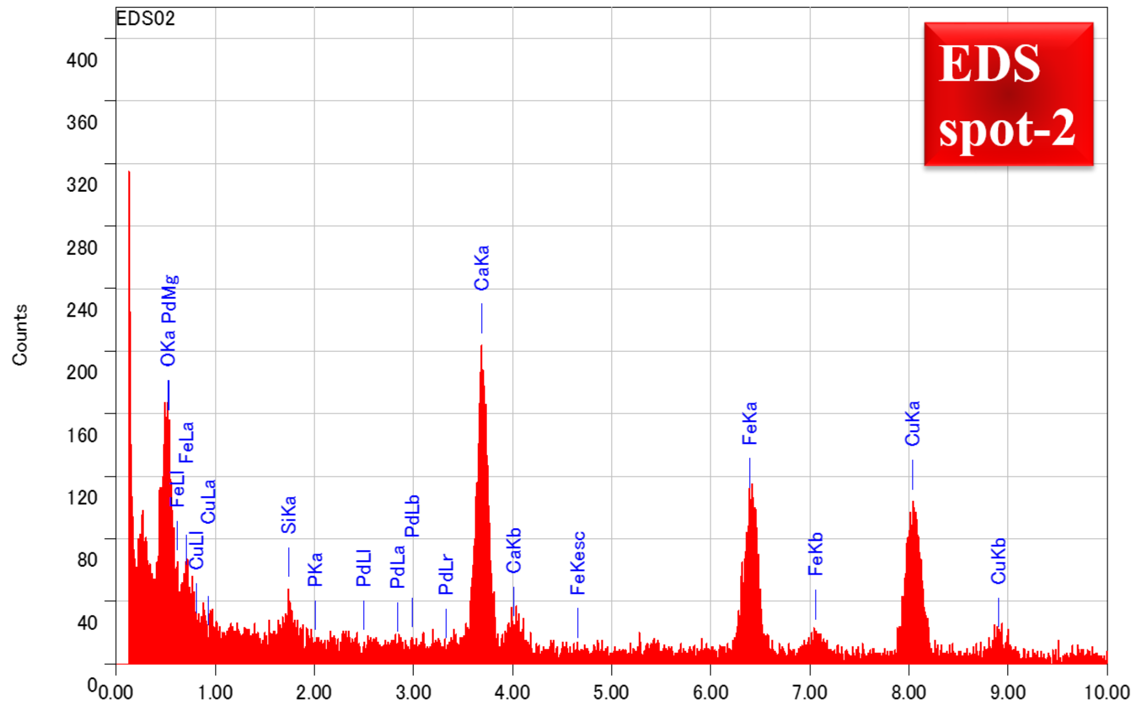


Thin film EDS quantitative analysis
Fitting coefficient : 0.5755

| element | (keV) | mass% | count | error% | atomic% |
|---|---|---|---|---|---|
| Cu K | 8.040 | 32.69 | 1470.08 | 0.05 | 20.29 |
| Fe K | 6.398 | 25.21 | 1445.88 | 0.04 | 17.81 |
| Ca K(standard) | 3.690 | 24.20 | 1889.39 | 0.03 | 23.82 |
| O K | 0.525 | 13.83 | 692.68 | 0.04 | 34.11 |
| Si K | 1.739 | 2.23 | 203.18 | 0.29 | 3.14 |
| Pd L | 2.838 | 1.67 | 39.66 | 1.34 | 0.62 |
| P K | 2.013 | 0.17 | 14.07 | 3.55 | 0.21 |
| total | | 100.00 | | | 100.00 |

3

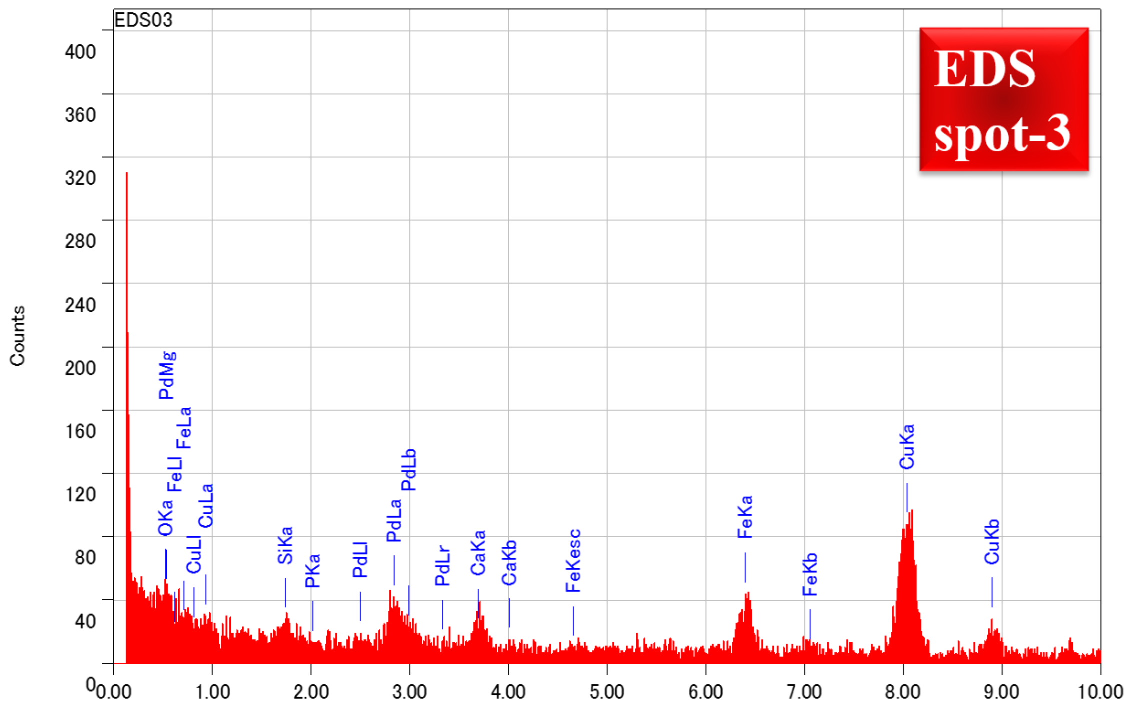


Thin film EDS quantitative analysis
Fitting coefficient : 0.7454

| element | (keV) | mass% | count | error% | atomic% |
|---|---|---|---|---|---|
| Cu K(standard) | 8.040 | 57.24 | 1256.30 | 0.05 | 49.13 |
| Fe K | 6.398 | 15.50 | 433.85 | 0.13 | 15.14 |
| Pd L | 2.838 | 13.78 | 159.77 | 0.32 | 7.06 |
| Ca K | 3.690 | 6.04 | 230.00 | 0.25 | 8.21 |
| O K | 0.525 | 4.22 | 103.13 | 0.23 | 14.38 |
| Si K | 1.739 | 2.16 | 95.93 | 0.58 | 4.20 |
| P K | 2.013 | 1.07 | 43.78 | 1.09 | 1.88 |
| 合計 | | 100.00 | | | 100.00 |

4

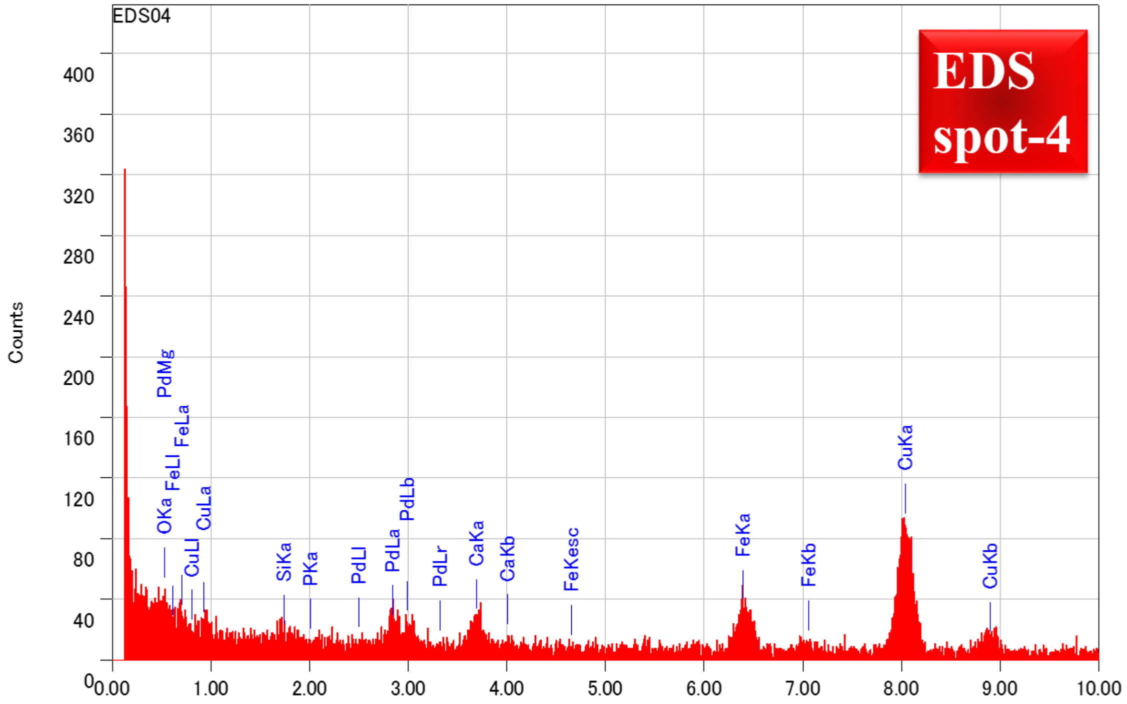


Thin film EDS quantitative analysis
Fitting coefficient : 0.7274

| element | (keV) | mass% | count | error% | atomic% |
|---|---|---|---|---|---|
| Cu K(standard) | 8.040 | 57.06 | 1354.86 | 0.05 | 54.03 |
| Pd L | 2.838 | 20.17 | 253.10 | 0.22 | 11.40 |
| Fe K | 6.398 | 13.84 | 419.24 | 0.15 | 14.92 |
| Ca K | 3.690 | 5.65 | 232.94 | 0.27 | 8.48 |
| O K | 0.525 | 2.58 | 68.11 | 0.38 | 9.69 |
| Si K | 1.739 | 0.63 | 30.12 | 2.01 | 1.34 |
| P K | 2.013 | 0.07 | 3.00 | 17.43 | 0.13 |
| total | | 100.00 | | | 100.00 |

5

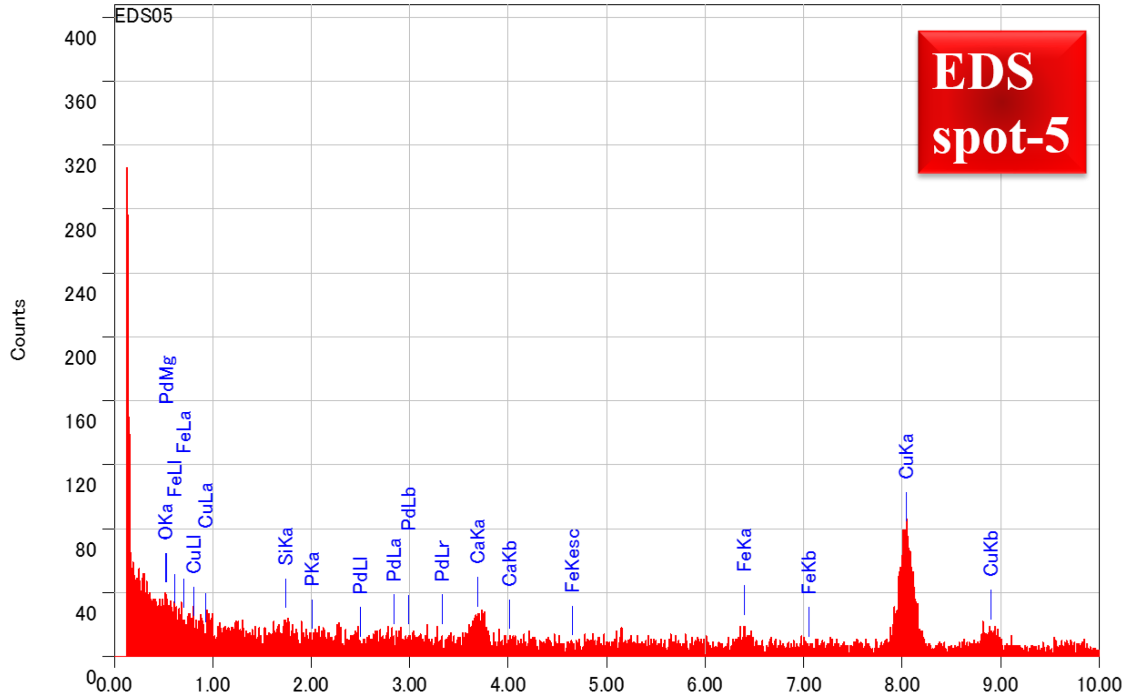


Thin film EDS quantitative analysis
Fitting coefficient : 0.8086

| element | (keV) | mass% | count | error% | atomic% |
|---|---|---|---|---|---|
| Cu K(standard) | 8.040 | 79.51 | 1180.94 | 0.06 | 67.01 |
| Fe K | 6.398 | 6.45 | 122.19 | 0.51 | 6.19 |
| Ca K | 3.690 | 5.50 | 141.94 | 0.46 | 7.35 |
| O K | 0.525 | 4.09 | 67.70 | 0.39 | 13.70 |
| Si K | 1.739 | 2.51 | 75.22 | 0.82 | 4.78 |
| Pd L | 2.838 | 1.94 | 15.24 | 3.71 | 0.98 |
| P K | | | | | |
| total | | 100.00 | | | 100.00 |

On the other hand, in SEM-EDS spectra of MNPs@$SiO_2$-$NH_2$@Pd(dpa)$Cl_2$ nanoparticles (Figure S18), we can see Pd and Cl elements at the same position. The XPS peak was observed at 338.39 eV, being assigned to $PdCl_2$. This assignment is supported by SEM-EDS result.

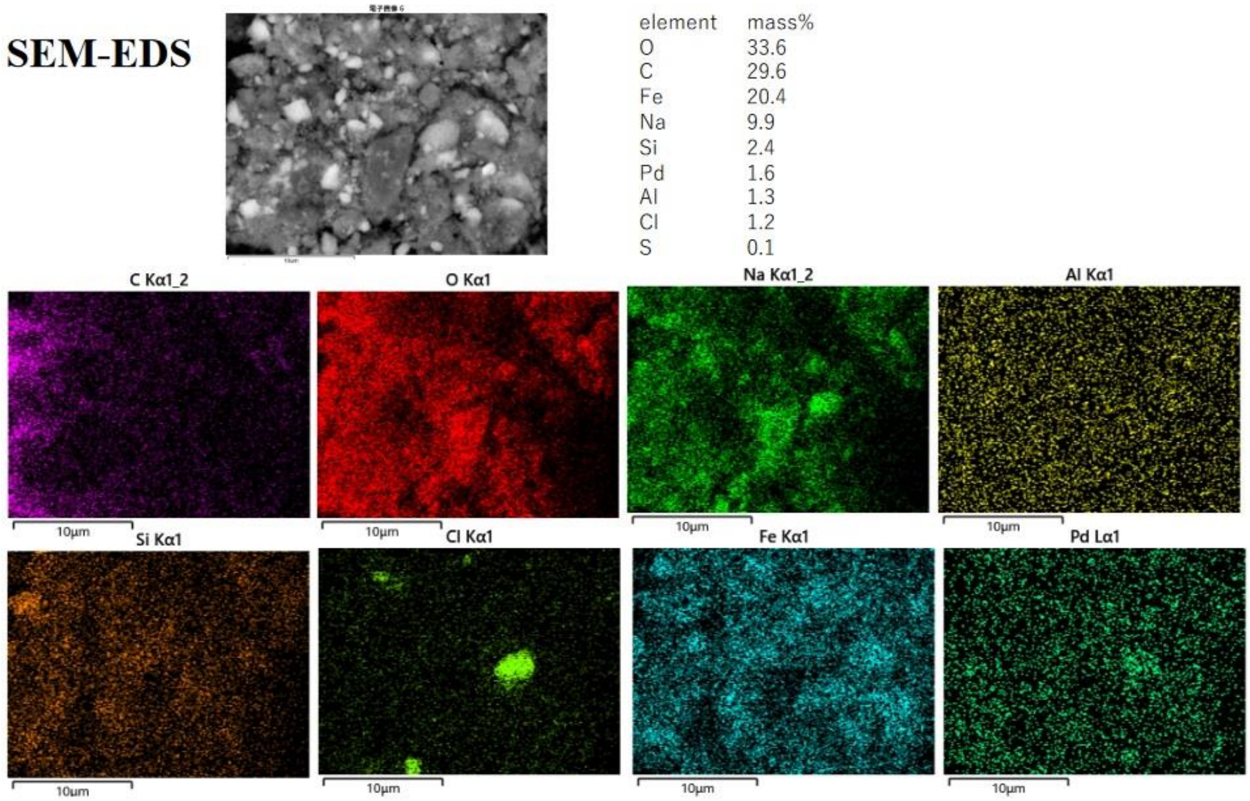


**Figure S18.** SEM-EDS spectra of MNPs@$SiO_2$-$NH_2$@Pd(dpa)$Cl_2$ nanoparticles.

The TEM spectra with particle size histogram of reused MNPs@$SiO_2$-$NH_2$@Pd(dpa)$Cl_2$ nanoparticles were represented in figure S19. It could be observed that the average particle size of MNPs@$SiO_2$-$NH_2$@Pd(dpa)$Cl_2$ nanoparticles was decreased from 5 to 3 nm comparing with fresh sample (Figure S17). It means that Pd is highly dispersed in reused MNPs@$SiO_2$-$NH_2$@Pd(dpa)$Cl_2$ sample.

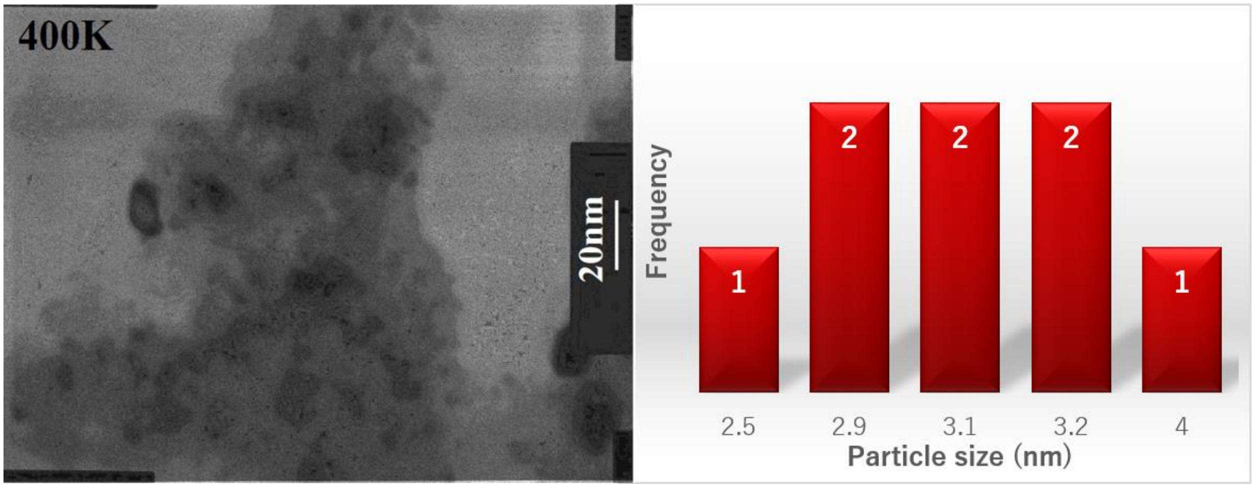


**Figure S19.** TEM spectra with particle size histogram of reused MNPs@$SiO_2$-$NH_2$@Pd(dpa)$Cl_2$ nanoparticles.

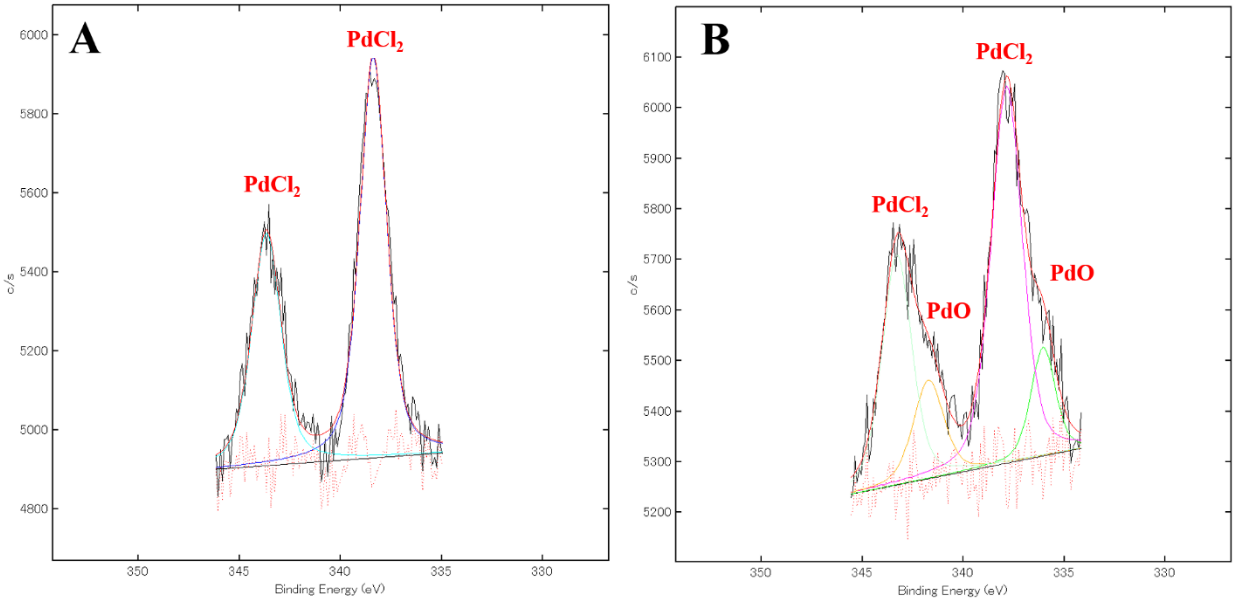


| | $3d_{5/2}$ | $3d_{3/2}$ |
|---|---|---|
| Binding energy /eV | 338.39 | 343.67 |

| | $3d_{5/2}$ | | $3d_{3/2}$ | |
|---|---|---|---|---|
| Binding energy /eV | 336.05 | 337.86 | 341.73 | 343.29 |
| Peak area /% | 10.99 | 48.29 | 12.44 | 28.28 |

**Figure S20.** Pd 3d core level photoelectron spectra of MNPs@$SiO_2$-$NH_2$@Pd(dpa)$Cl_2$ artificial metalloenzyme before (a), and after (b), reaction cycles.

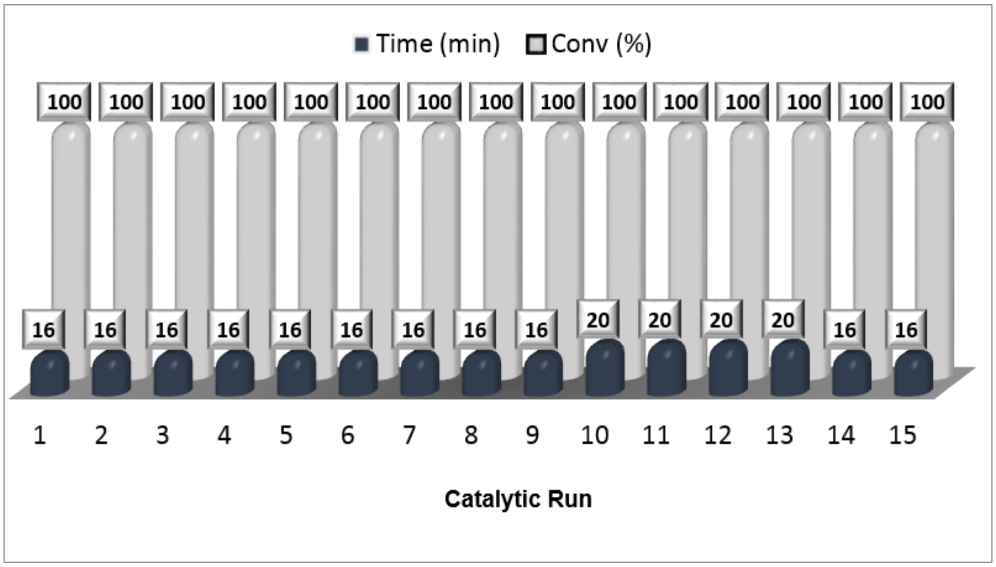


**Figure S21.** The recycling of MNPs@$SiO_2$-$NH_2$@Pd(dpa)$Cl_2$ for the Suzuki-Miyaura cross-coupling of iodobenzene and phenylboronic acid under optimum reaction conditions.

## Data of compounds

*[1,1'-biphenyl]-4-amine*[7]

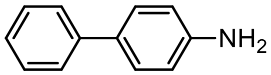


**IR (KBr) ($cm^{-1}$):** 3641, 3379, 1620, 1521, 1487, 828, 761, 696; **$^{1}$H NMR (250.13 MHz, $CDCl_3$, 25°C, TMS):** δ=7.41-7.59 (m, 6H), 7.32 (d, *J*=6.75Hz, 1H), 6.76 (d, *J*=7.75Hz, 2H), 3.68 (br, 2H); **$^{13}$C NMR (62.90 MHz, $CDCl_3$, 25 ºC, TMS):** 145.82, 141.18, 131.62, 128.68, 128.01, 126.41, 126.28, 115.44.

*[1,1'-biphenyl]-4-ylmethanol*

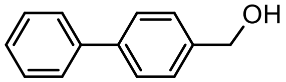


**IR (KBr) ($cm^{-1}$):** 3606, 3345, 2251, 1908, 1602, 1566, 1488, 1212, 899; **$^{1}$H NMR (250.13 MHz, $CDCl_3$, 25°C, TMS):** δ=7.39-7.63 (m, 9H), 4.72 (s, 2H), 2.58 (s, 1H); **$^{13}$C NMR (62.90 MHz, $CDCl_3$, 25 ºC, TMS):** 140.85, 140.53, 139.94, 128.84, 127.49, 127.29, 127.12, 64.91.

*4-nitro-1,1'-biphenyl*[8]

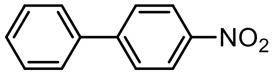


**IR (KBr) ($cm^{-1}$):** 1595, 1514, 1349, 852, 739, 699; **$^{1}$H NMR (250.13 MHz, $CDCl_3$, 25°C, TMS):** δ=8.23-8.36 (m, 2H), 7.71-7.80 (m, 2H), 7.58-7.64 (m, 2H), 7.48-7.51 (m, 3H); **$^{13}$C NMR (62.90 MHz, $CDCl_3$, 25 ºC, TMS):** 147.63, 147.09, 138.76, 135.59, 132.63, 129.13, 128.90, 127.95, 127.77, 127.36, 124.04.

*3-nitro-1,1'-biphenyl*

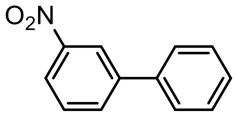


**IR (KBr) ($cm^{-1}$):** 1526, 1348, 763, 729, 697; **$^{1}$H NMR (250.13 MHz, $CDCl_3$, 25°C, TMS): δ=8.49-8.45 (m, 1H),** 8.29 (d, *J*=7 50 Hz, 1H), 8.19 (d, *J*=7 50 Hz, 1H), 7.94 (t, *J*=10.12 Hz, 1H), 7.47-7.73 (m, 5H); **$^{13}$C NMR (62.90 MHz, $CDCl_3$, 25 ºC, TMS):** 148.77, 142.88, 138.64,133.01, 129.68, 129.14, 128.52, 127.14, 123.27, 122.00.

*1-phenylnaphthalene*[9]

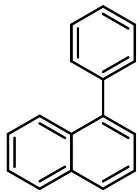

**IR (KBr) (cm$^{-1}$):** 1591, 1493, 1395, 801, 778, 702, 616; **$^{1}$H NMR (250.13 MHz, $CDCl_3$, 25°C, TMS):** δ=7.97-8.03 (m, 3H), 7.53 (br, 6H), 7.57 (br, 3H); **$^{13}$C NMR (62.90 MHz, $CDCl_3$, 25 ºC, TMS):** 140.86, 140.36, 133,90, 131.73, 130.16, 128,34, 127.93, 127.71, 127.31, 127.01, 126.65, 126.10, 125.84, 125.45.

*1-(p-tolyl)naphthalene*[9]

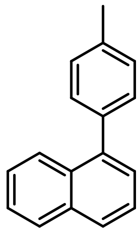

**IR (KBr) (cm$^{-1}$):** 3044, 2919, 1591, 1505, 1394, 821, 799, 776; **$^{1}$H NMR (250.13 MHz, $CDCl_3$, 25°C, TMS):** δ=7.89-8.03 (m, 3H), 7.35-7.67 (m, 8H), 2.52 (s, 3H); **$^{13}$C NMR (62.90 MHz, $CDCl_3$, 25 ºC, TMS): 140.33, 137.90, 136.94, 133.90, 131.81, 130.01, 129.03, 128.31, 127.90, 127.50, 126.94, 126.62, 126.17, 125.97, 125.76, 125,45, 21.28.**

*2-phenylnaphthalene*[9]

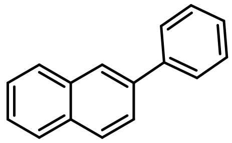

**IR (KBr) (cm$^{-1}$):** 1596, 1452, 1361, 821, 770, 757, 688; **$^{1}$H NMR (250.13 MHz, $CDCl_3$, 25°C, TMS):** δ=8.07 (s, 1H), 7.92 (br, 3H), 7.74-7.77 (m, 3H), 7.41-7.63 (m, 5H); **$^{13}$C NMR (62.90 MHz, $CDCl_3$, 25 ºC, TMS):** 141.16, 138.58, 133.71, 132.64, 128.85, 128.41, 128.20, 127.64, 127.42, 126.28, 125.92, 125.81, 125.59.

*2-(p-tolyl)naphthalene*[9]

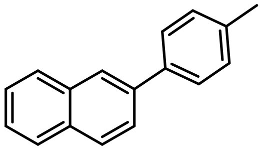

**IR (KBr) ($cm^{-1}$):** 3053, 2920, 1501, 1407, 809, 740, 615; **$^{1}H$ NMR (250.13 MHz, $CDCl_3$, 25°C, TMS):** δ=8.12 (s, 1H), 7.96 (br, 3H), 7.74-7.77 (m, 3H), 7.83 (d, *J*=8.25 Hz, 1H), 7.72 (d, *J*=7.50 Hz, 2H), 7.56 (t, *J*=3.50 Hz, 2H), 7.38 (d, *J*=7.50 Hz, 2H), 2.51 (s, 3H); **$^{13}C$ NMR (62.90 MHz, $CDCl_3$, 25 ºC, TMS):** 138.57, 138.31, 137.20, 133,85, 132.62, 129.68, 128.44, 128.24, 127.72, 127.33, 126.30, 126.09, 125.84, 125.63, 125.51, 21.20.

*9-phenylphenanthrene*

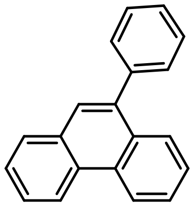

**IR (KBr) ($cm^{-1}$):** 1593, 1491, 1450, 776, 767, 746, 725, 711; **$^{1}H$ NMR (250.13 MHz, $CDCl_3$, 25°C, TMS):** δ=8.81 (d, *J*=8.25, 1H), 8.76 (d, *J*=8.00, 1H), 7.97 (d, *J*=9.00, 1H), 7.94 (d, *J*=10.25, 1H), 7.58-7.73 (m, 10H); **$^{13}C$ NMR (62.90 MHz, $CDCl_3$, 25 ºC, TMS):** 140.86, 138.83, 131.62, 131.20, 130.67, 128.69, 128.33, 127.54, 127.39, 126.95, 126.87, 126.51, 122.93, 122.56.

*9-(p-tolyl)anthracene*

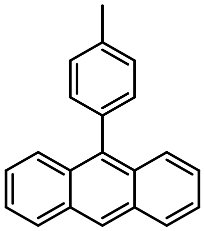

**IR (KBr) ($cm^{-1}$):** 1640, 1273, 879, 722, 694; **$^{1}H$ NMR (250.13 MHz, $CDCl_3$, 25°C, TMS):** δ=7.36-8.49 (m, 13H), 2.54 (s, 3H); **$^{13}C$ NMR (62.90 MHz, $CDCl_3$, 25 ºC, TMS):** 134.01, 131.69, 131.12, 129.06, 128.82, 128.16, 127.13, 126.94, 126.21, 125.80, 125.33, 20.87.

*1,1'-biphenyl*[7]

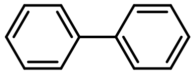

**IR (KBr) ($cm^{-1}$):** 1636, 1602, 1440, 1367, 1349, 1306, 694; **$^{1}H$ NMR (250.13 MHz, $CDCl_3$, 25°C, TMS):** δ=7.91 (d, *J*=7.50Hz, 4H), 7.50 (br, 6H).

# NMR and IR spectra of compounds

## [1,1'-biphenyl]-4-amine

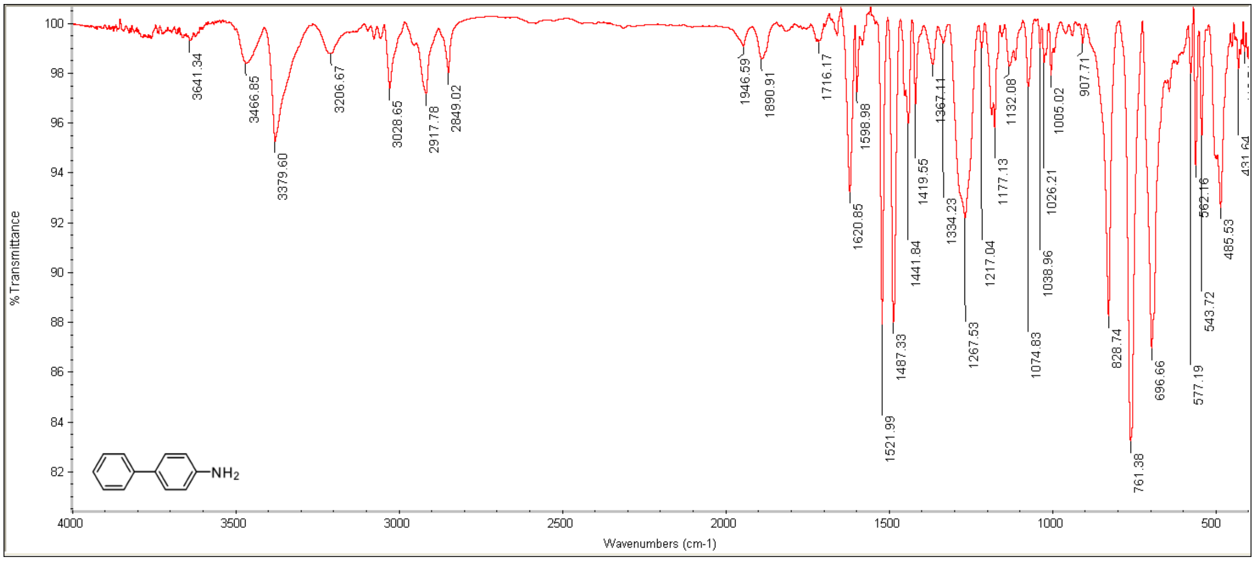

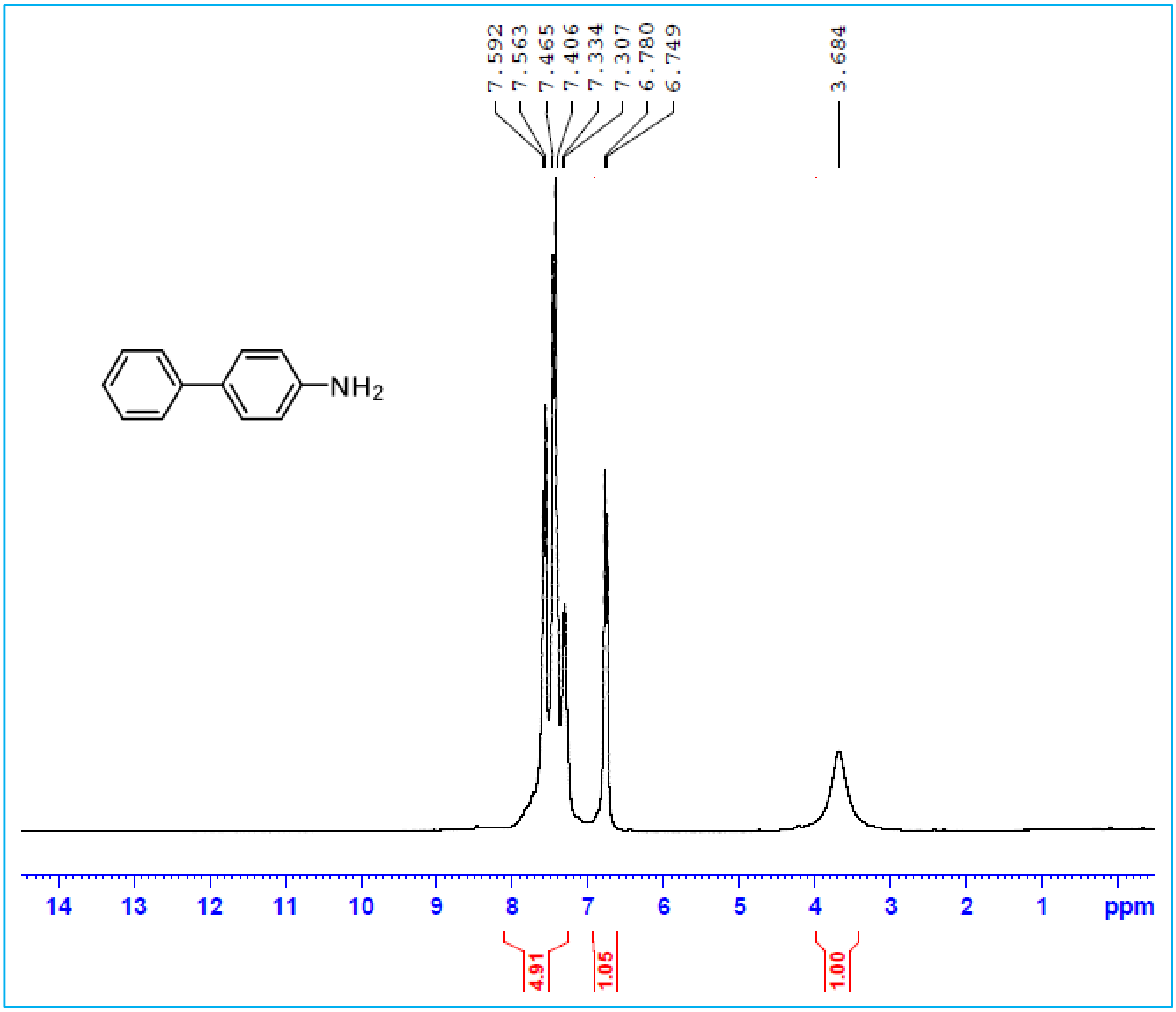

7.592
7.563
7.465
7.406
7.334
7.307
6.780
6.749
3.684
NH2
14 13 12 11 10 9 8 7 6 5 4 3 2 1 ppm
4.91
1.05
1.00

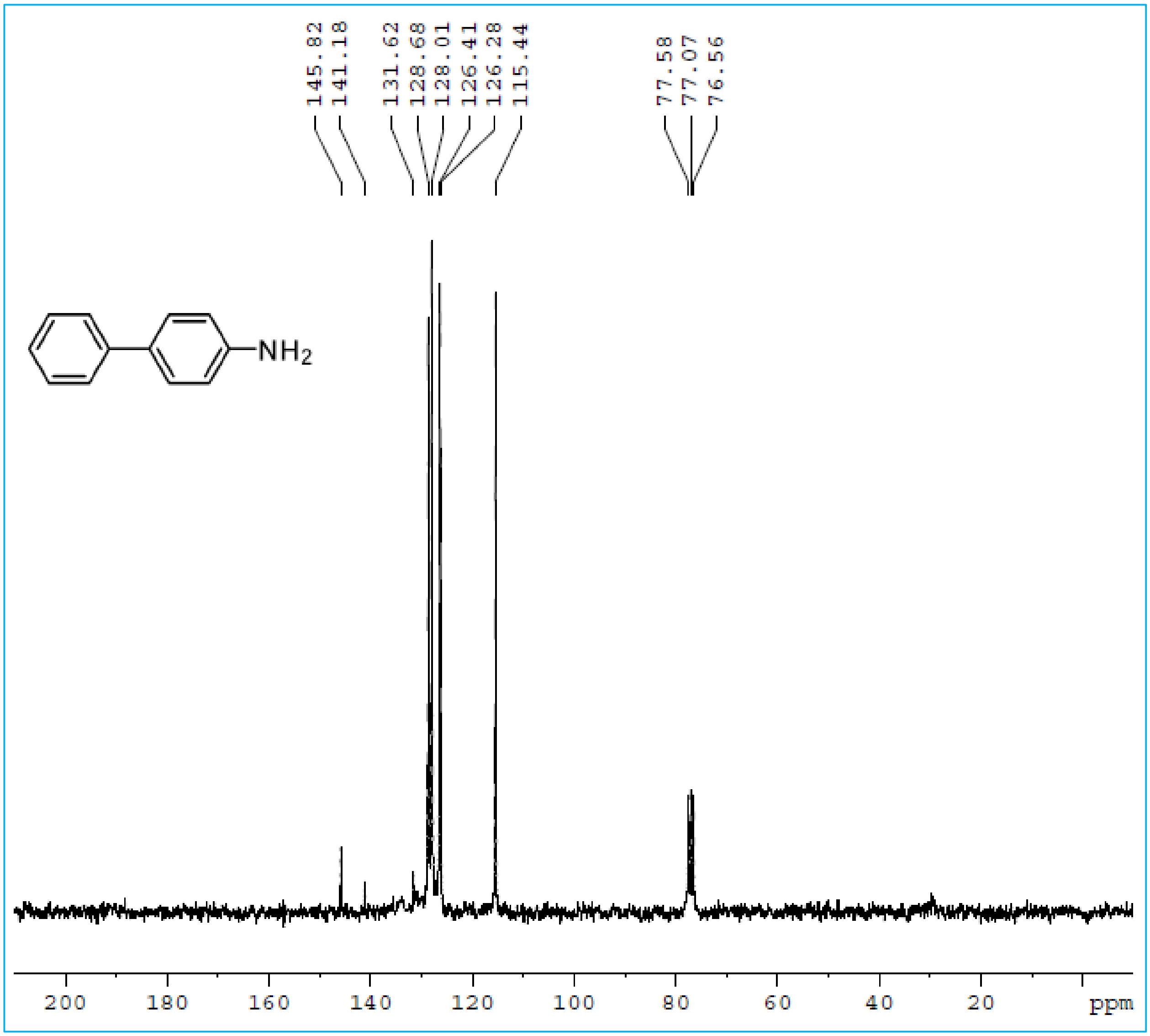
145.82
141.18
131.62
128.68
128.01
126.41
126.28
115.44
77.58
77.07
76.56
NH2
200
180
160
140
120
100
80
60
40
20
ppm

## [1,1'-biphenyl]-4-ylmethanol

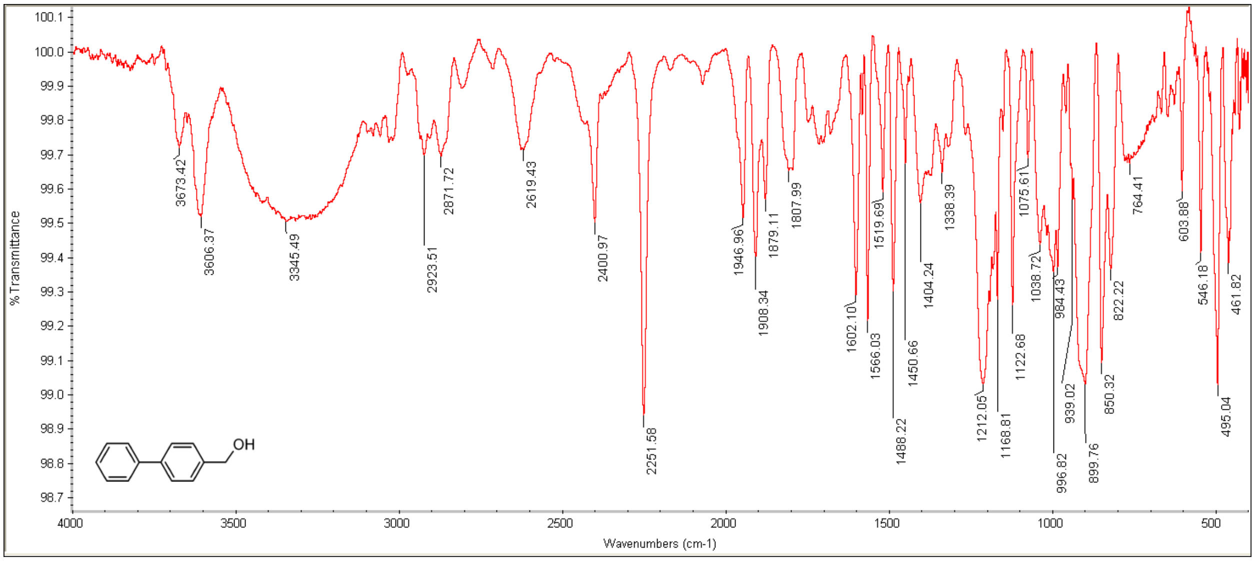


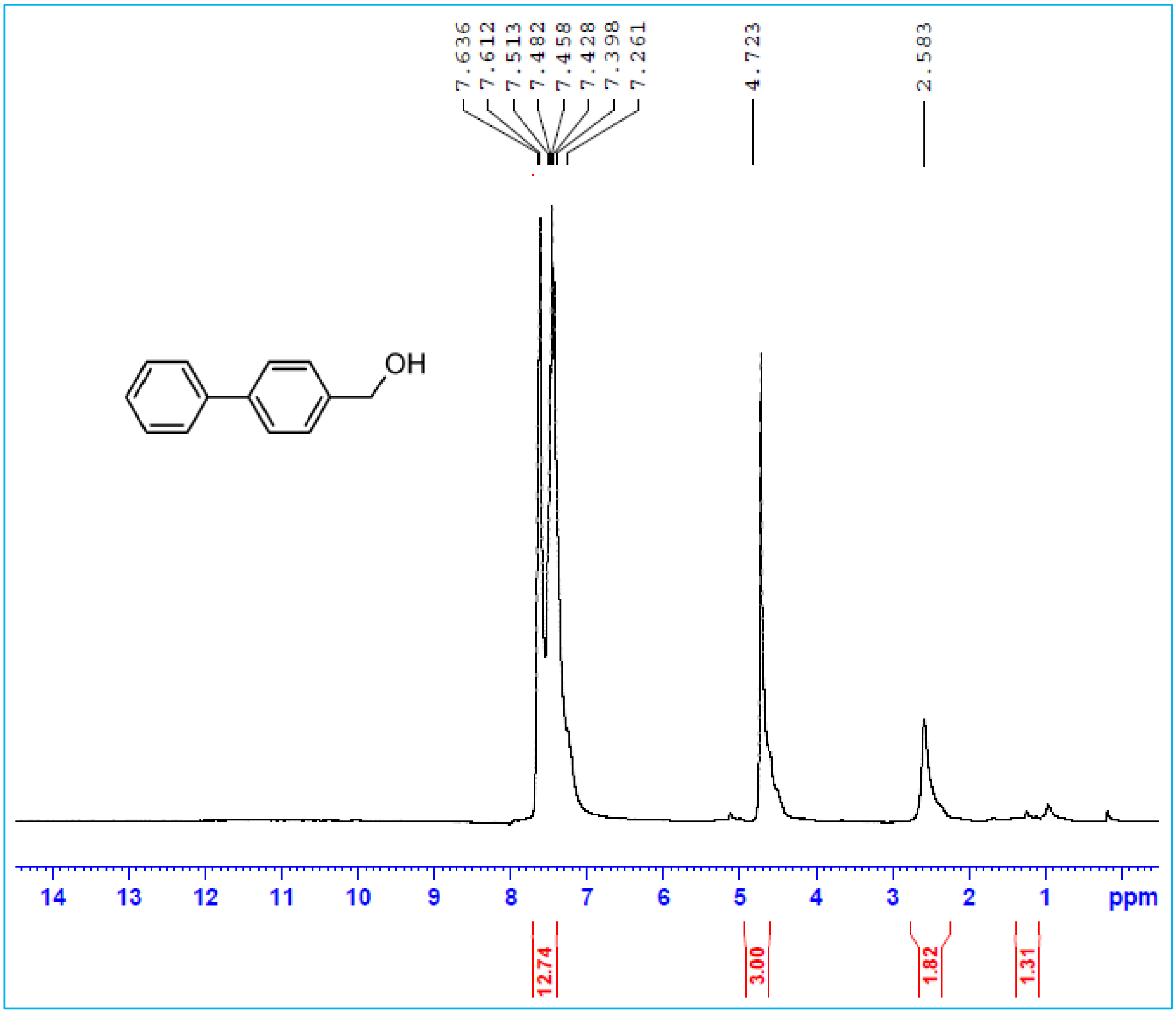

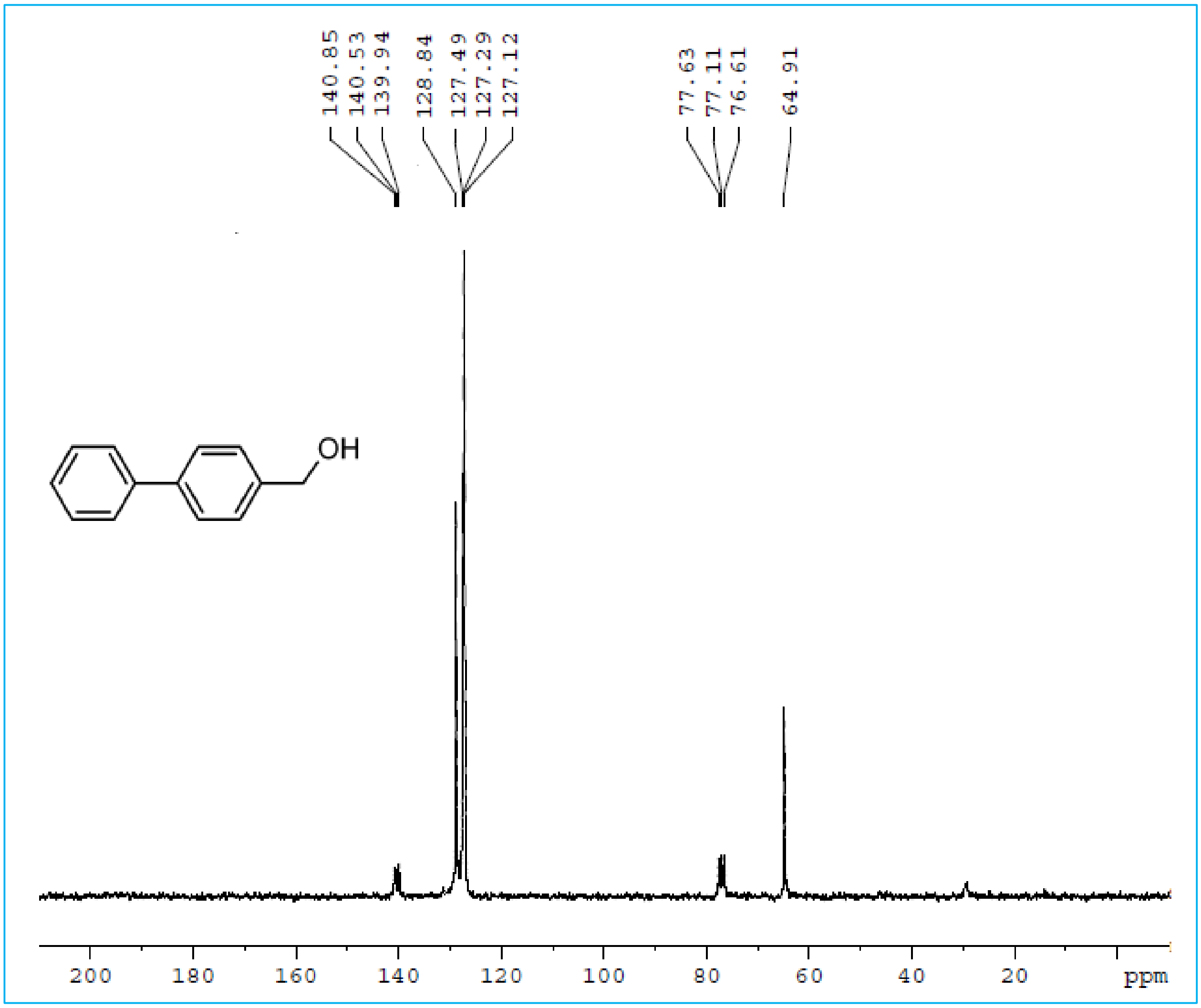

140.85
140.53
139.94
128.84
127.49
127.29
127.12
77.63
77.11
76.61
64.91
OH
200
180
160
140
120
100
80
60
40
20
ppm

**4-nitro-1,1'-biphenyl**

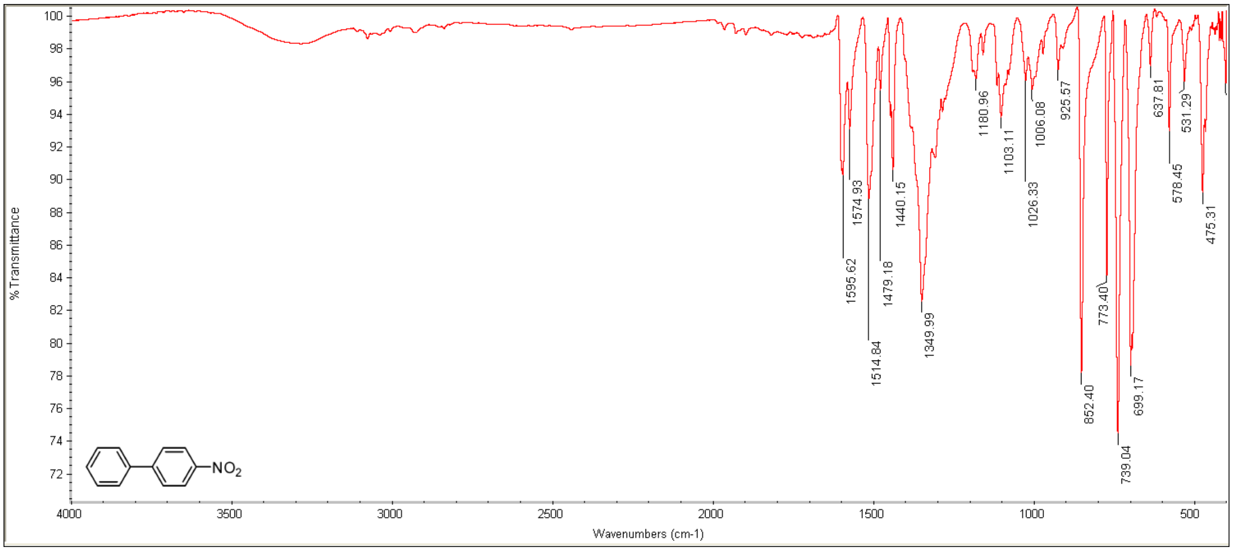


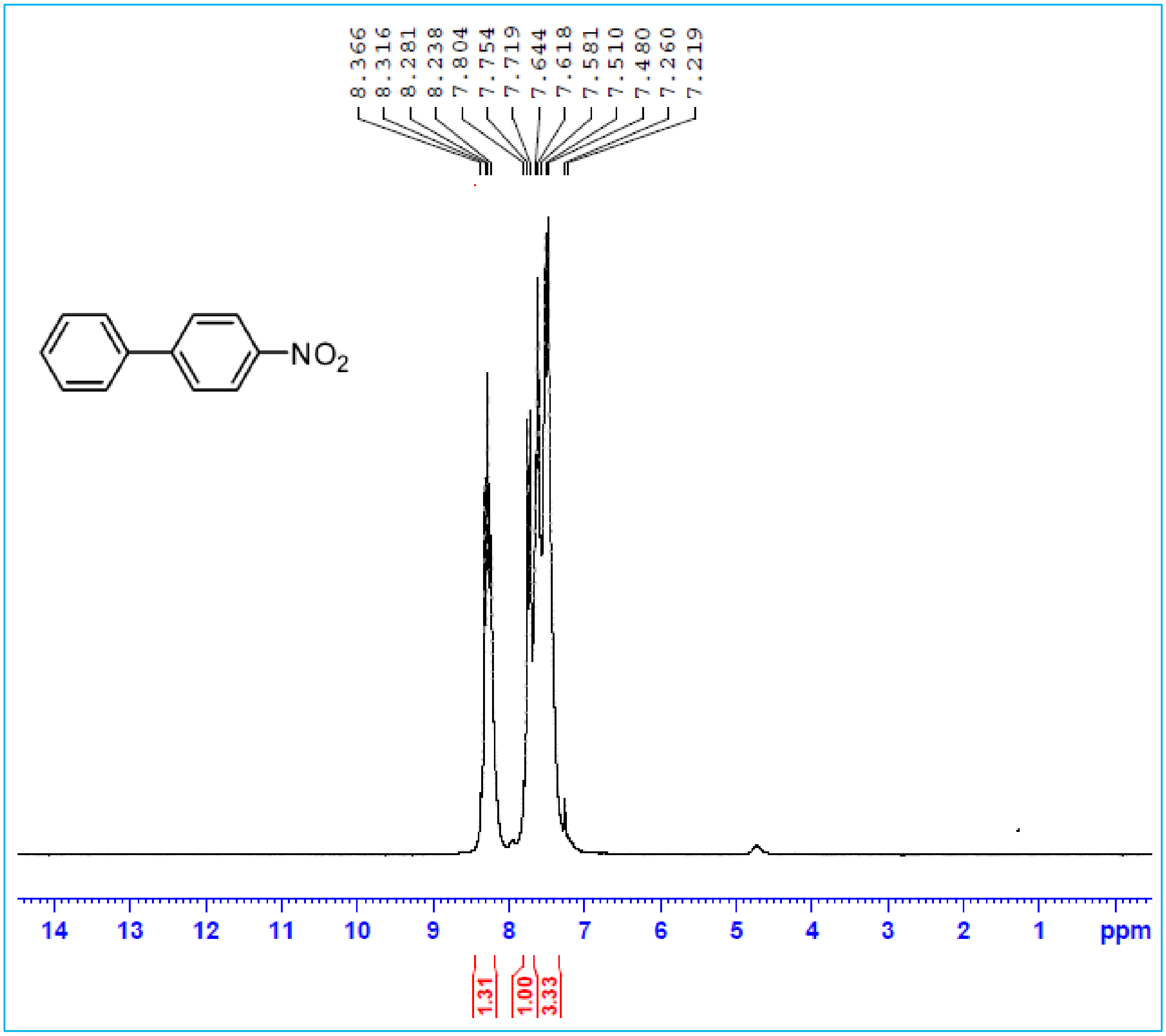

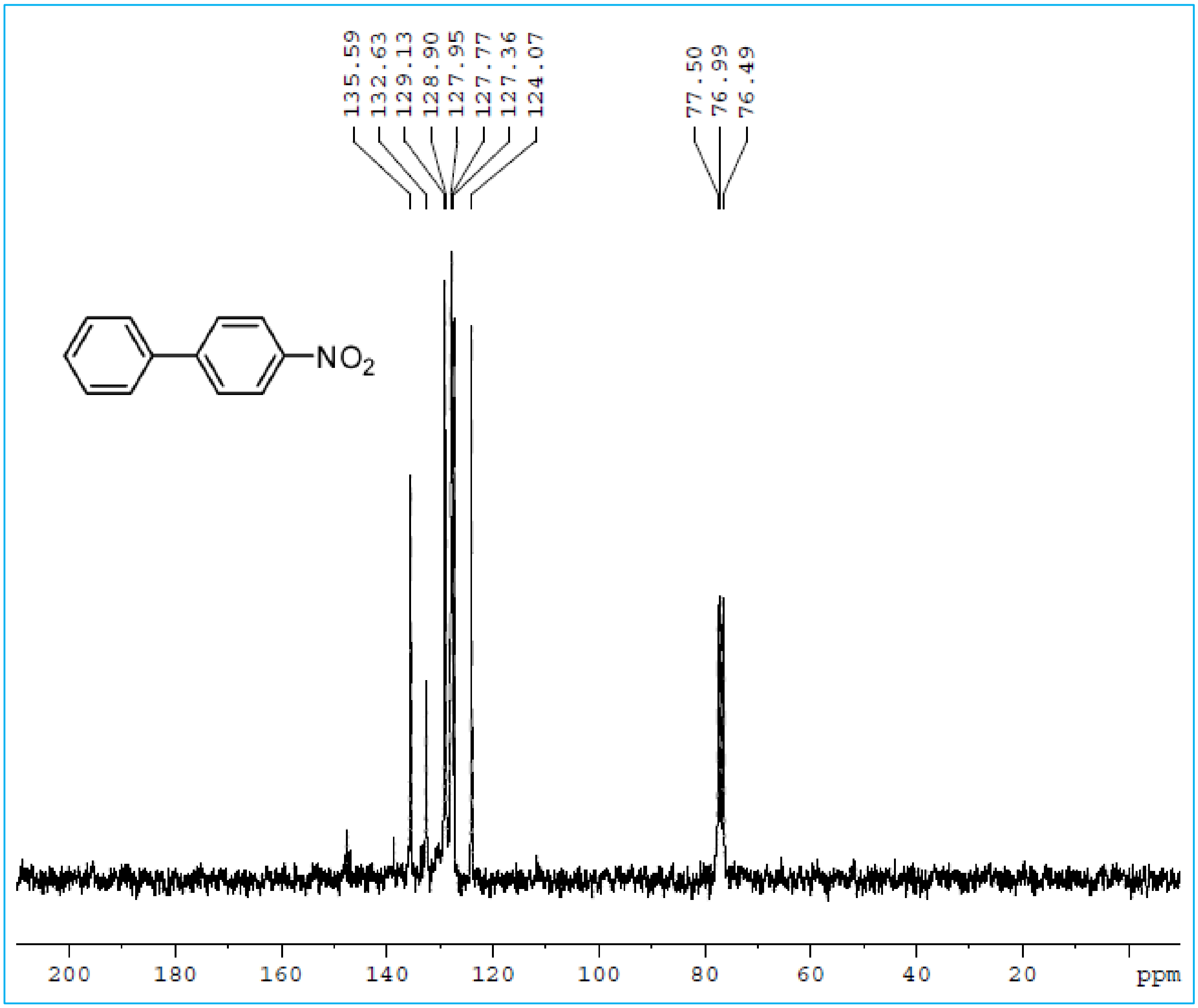
135.59
132.63
129.13
128.90
127.95
127.77
127.36
124.07
77.50
76.99
76.49
NO2
200
180
160
140
120
100
80
60
40
20
ppm

**3-nitro-1,1'-biphenyl**

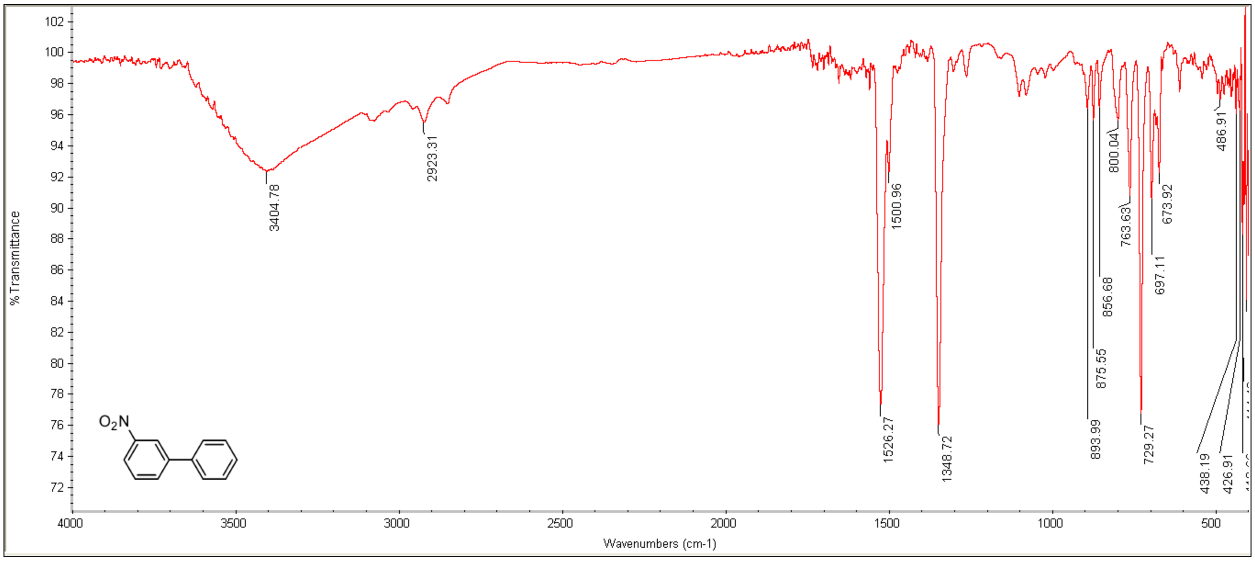


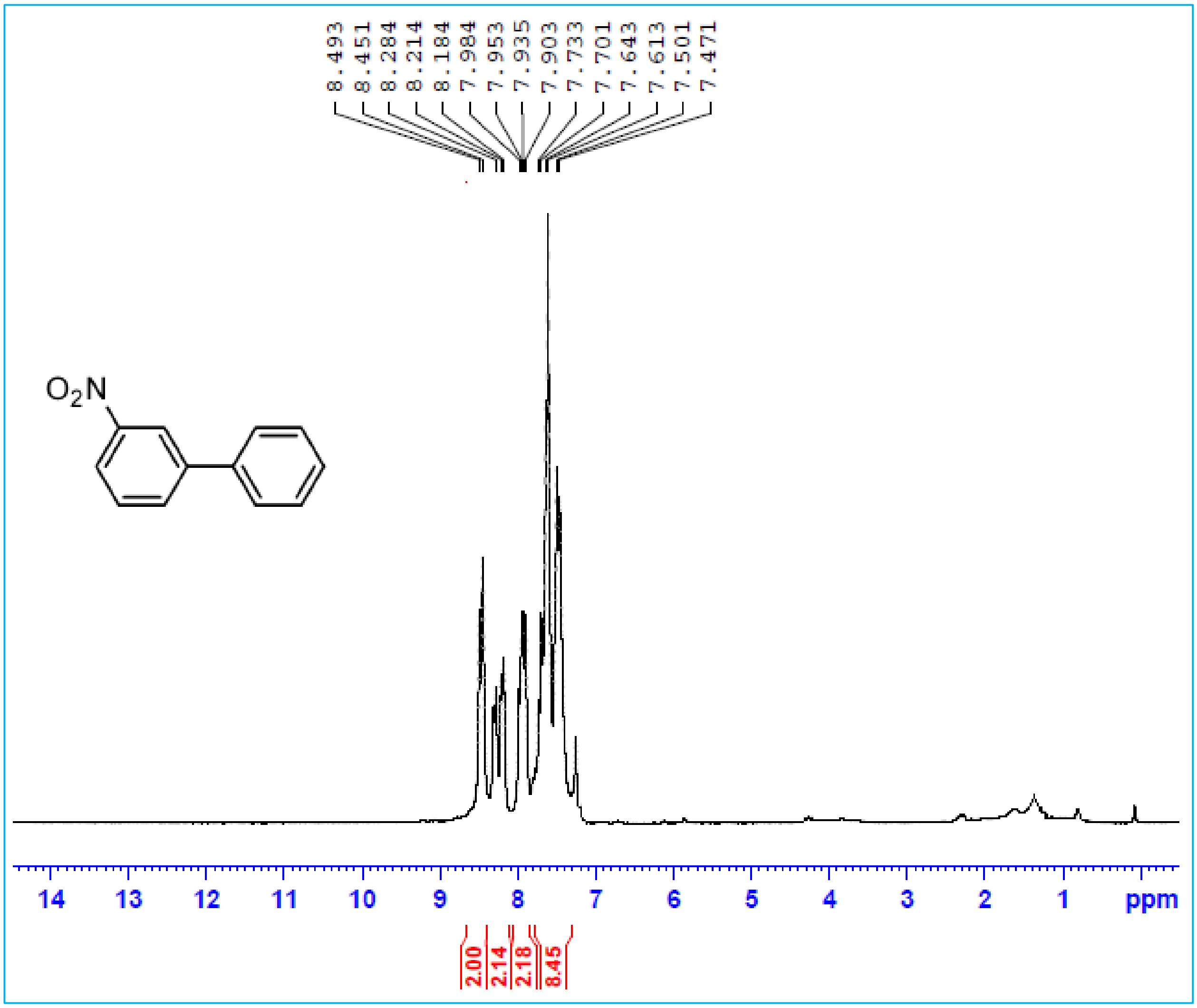

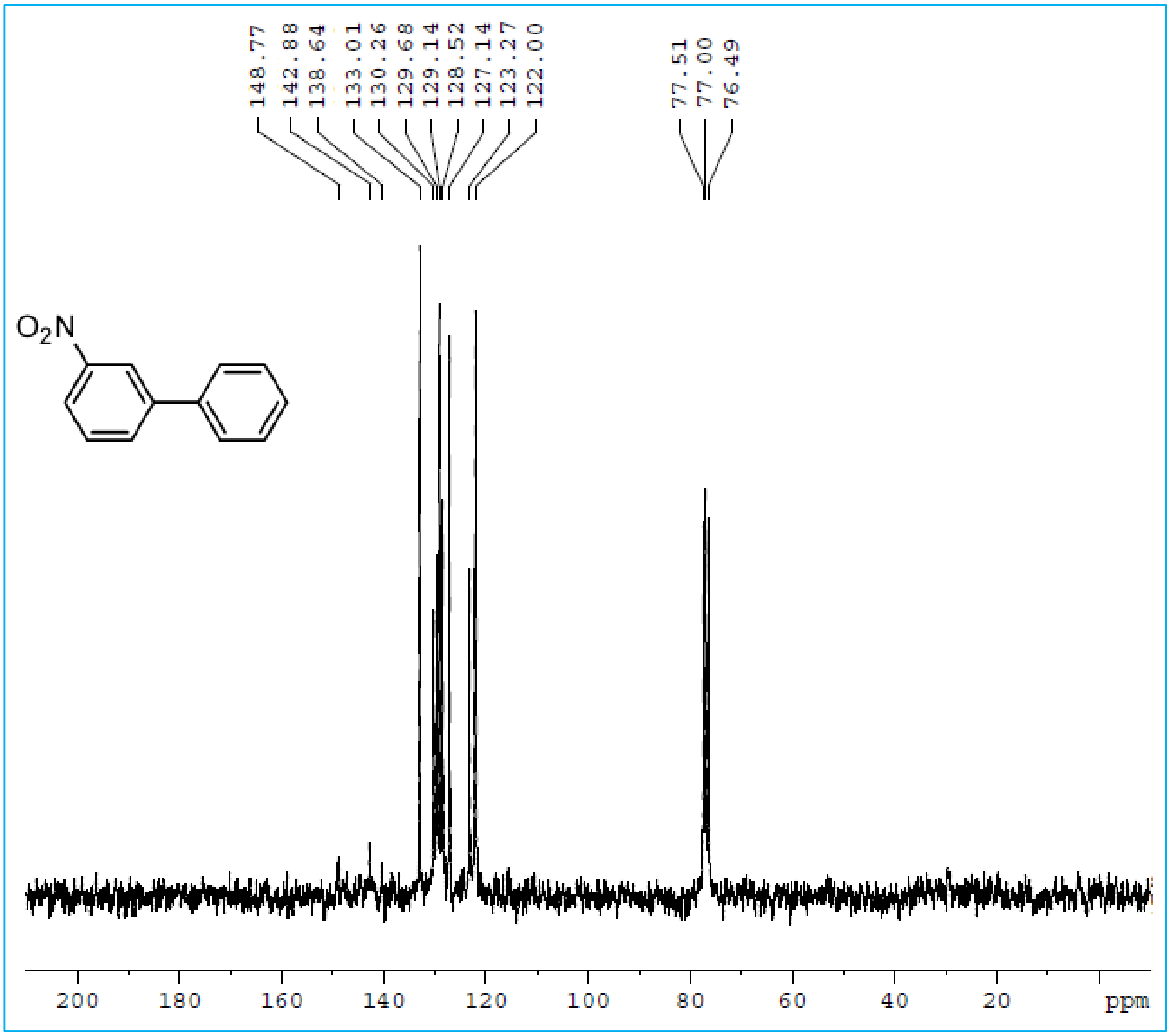
148.77
142.88
138.64
133.01
130.26
129.68
129.14
128.52
127.14
123.27
122.00
77.51
77.00
76.49
$O_2N$
200
180
160
140
120
100
80
60
40
20
ppm

## 1-phenylnaphthalene

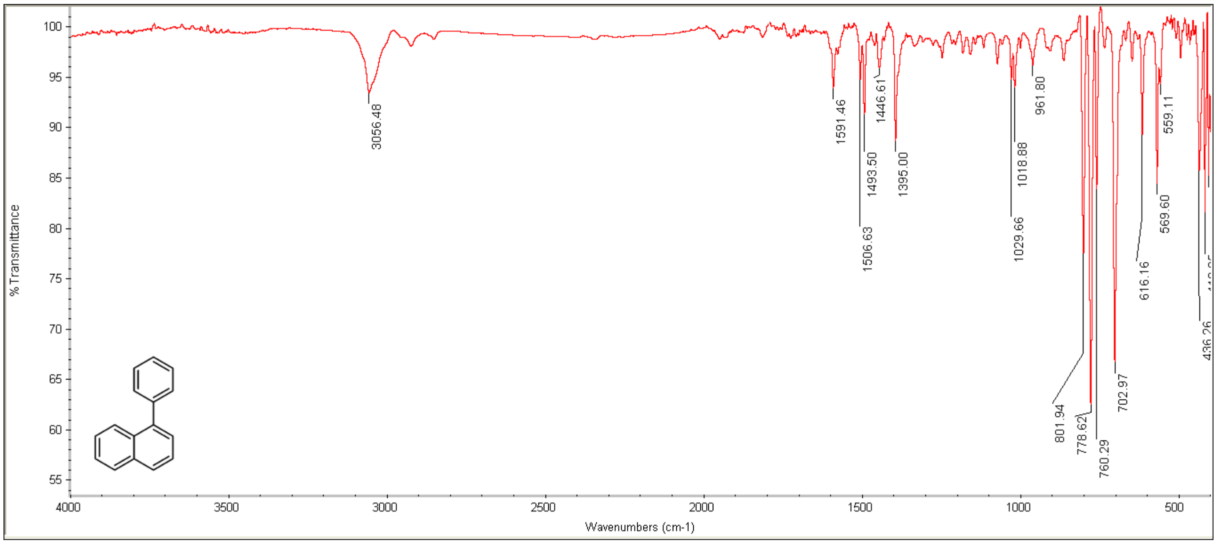


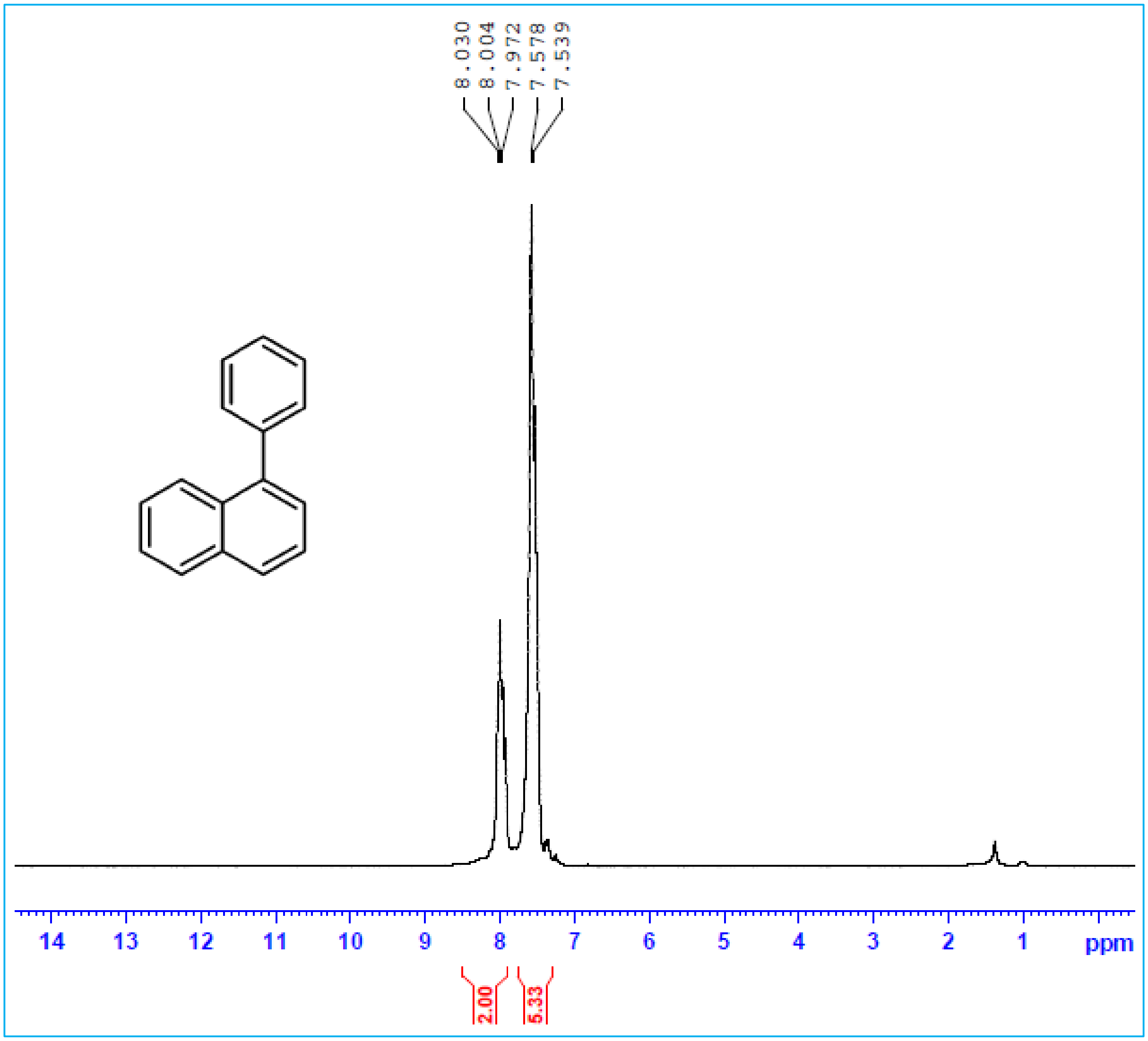

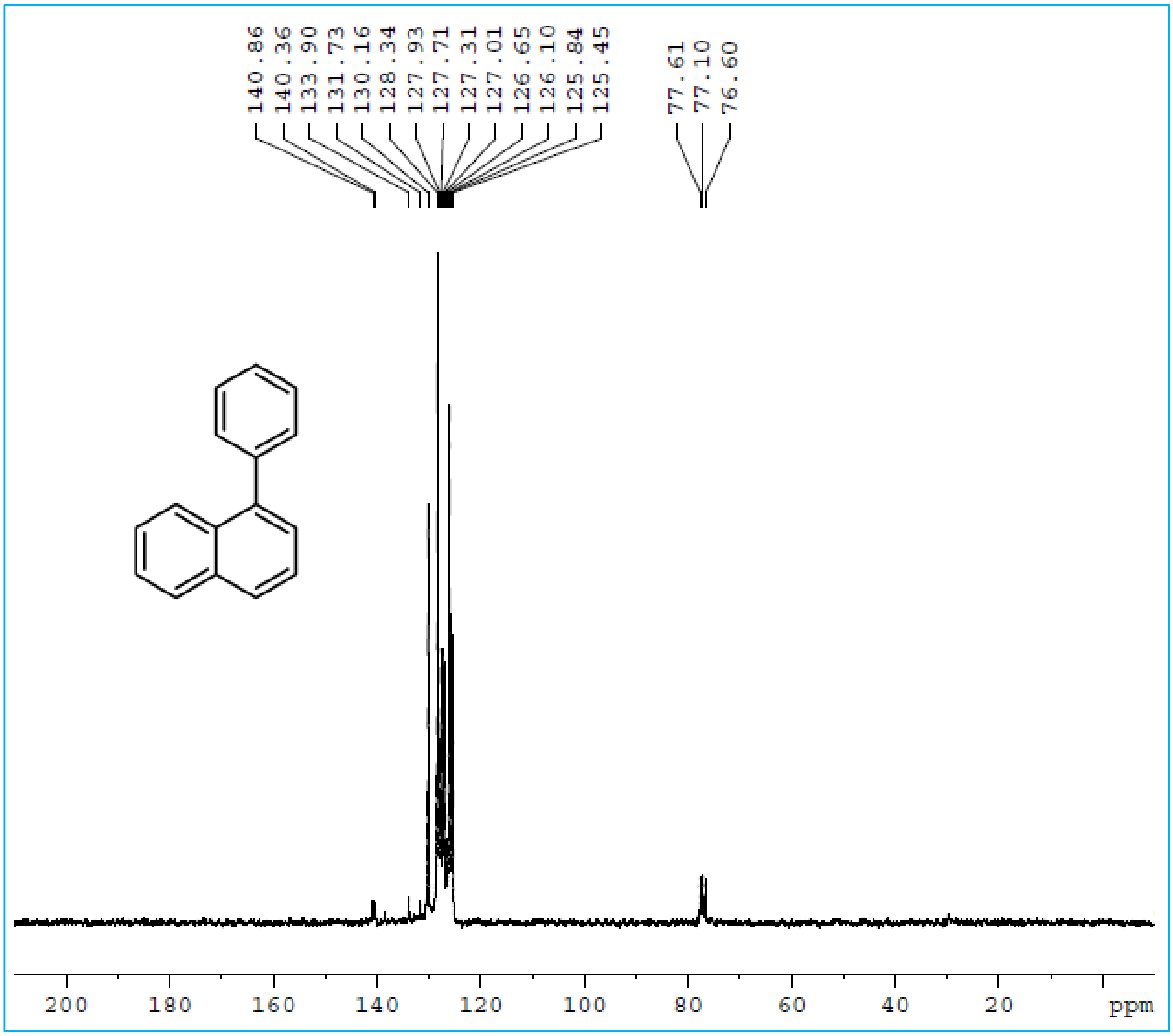
140.86
140.36
133.90
131.73
130.16
128.34
127.93
127.71
127.31
127.01
126.65
126.10
125.84
125.45
77.61
77.10
76.60
200
180
160
140
120
100
80
60
40
20
ppm

# 1-(p-tolyl)naphthalene

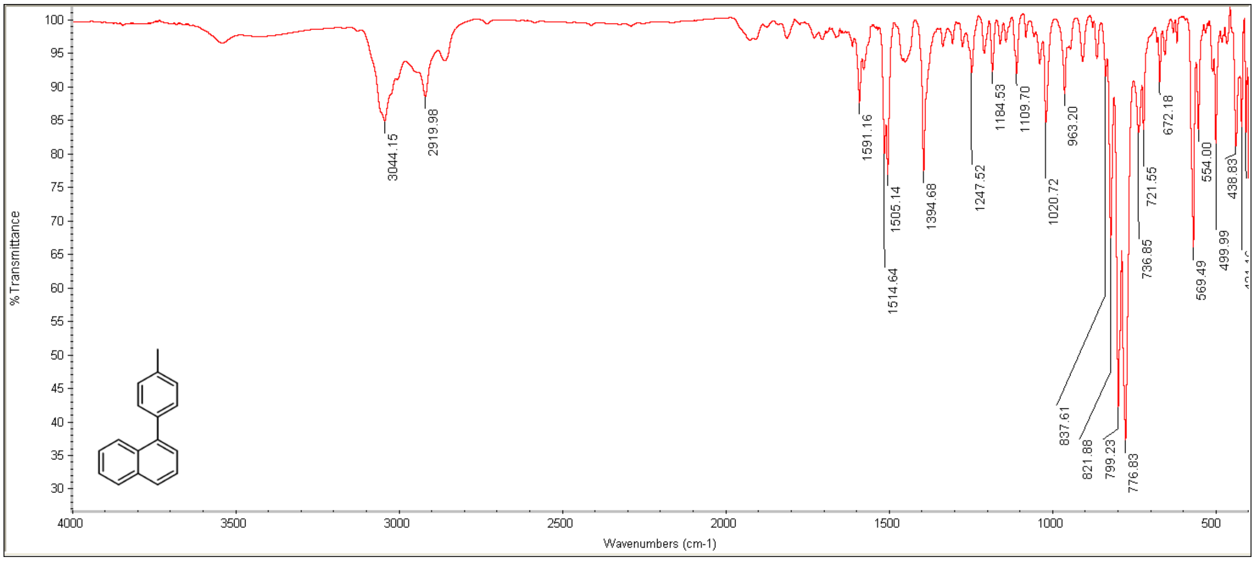


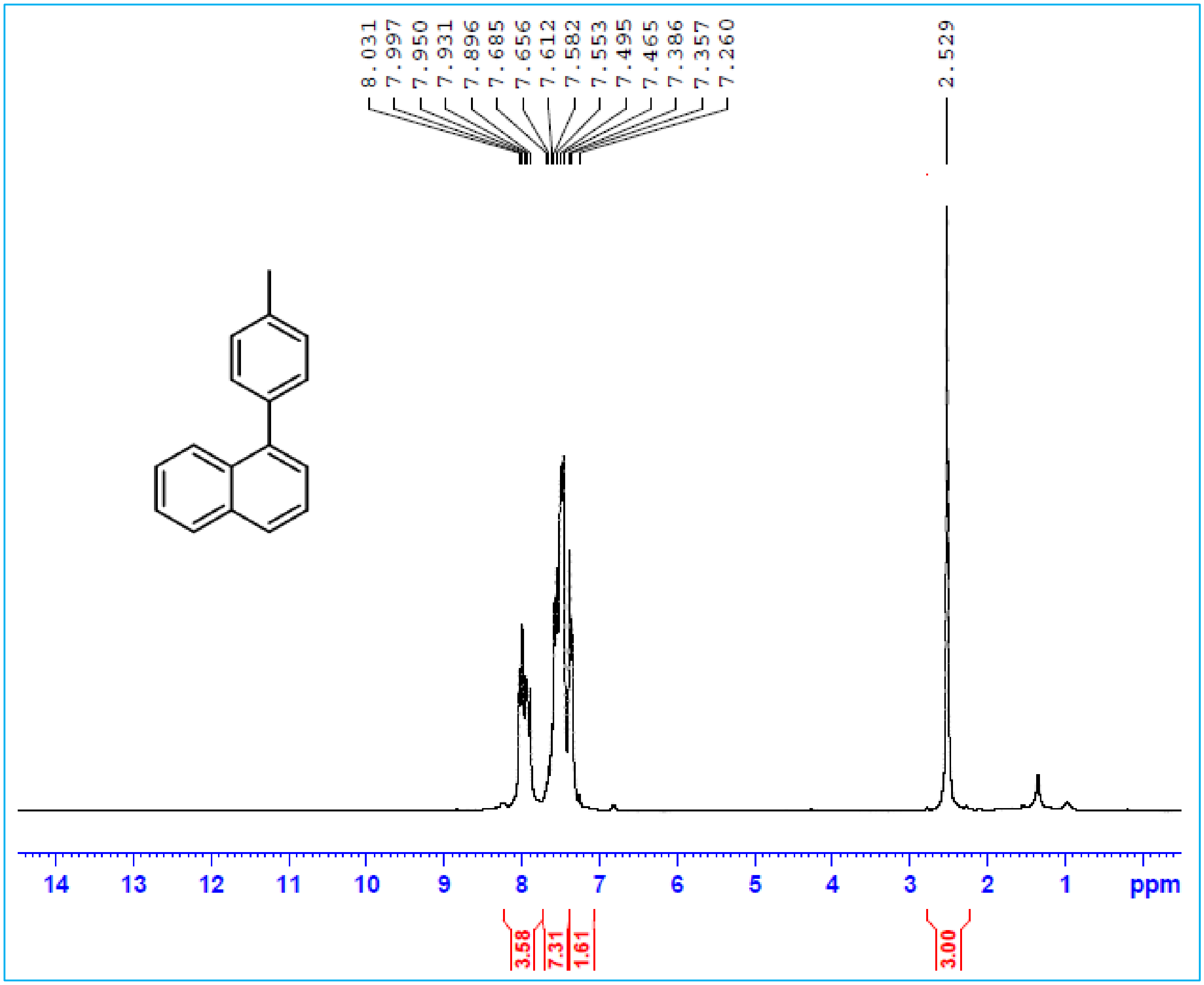

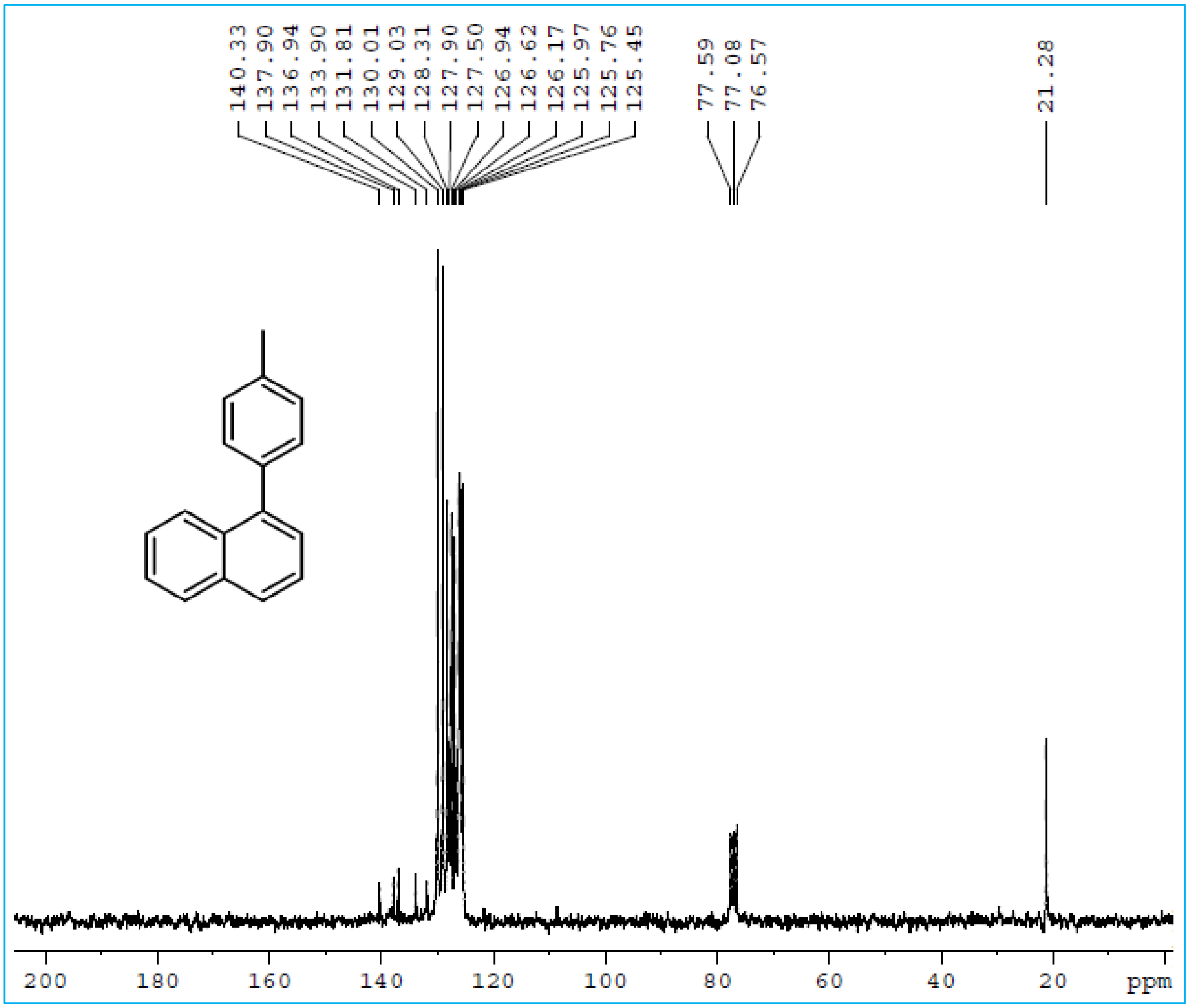
140.33
137.90
136.94
133.90
131.81
130.01
129.03
128.31
127.90
127.50
126.94
126.62
126.17
125.97
125.76
125.45
77.59
77.08
76.57
21.28
200
180
160
140
120
100
80
60
40
20
ppm

**2-phenylnaphthalene**

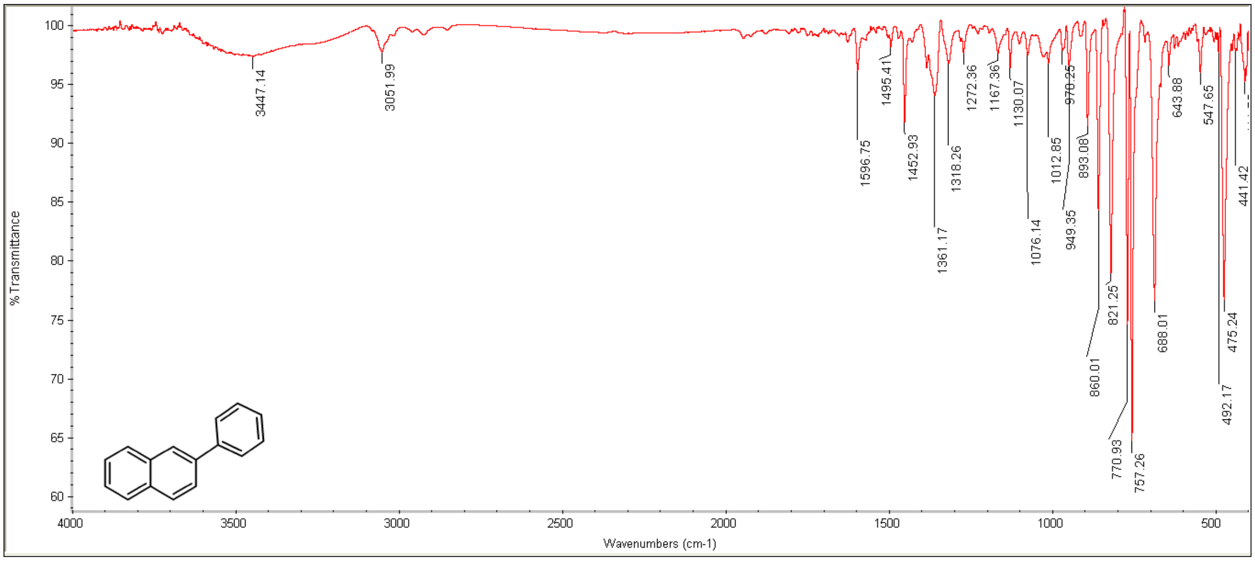


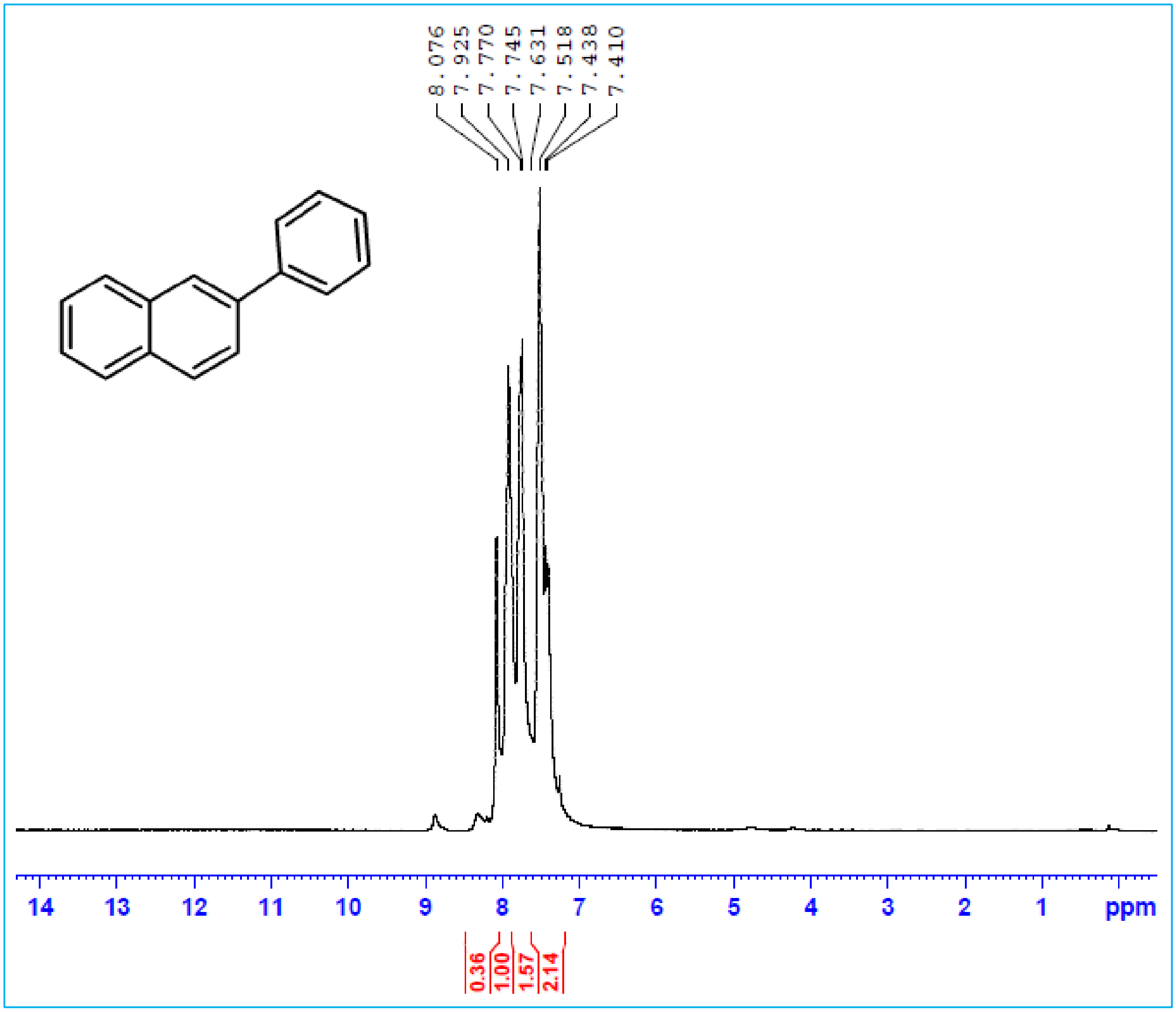

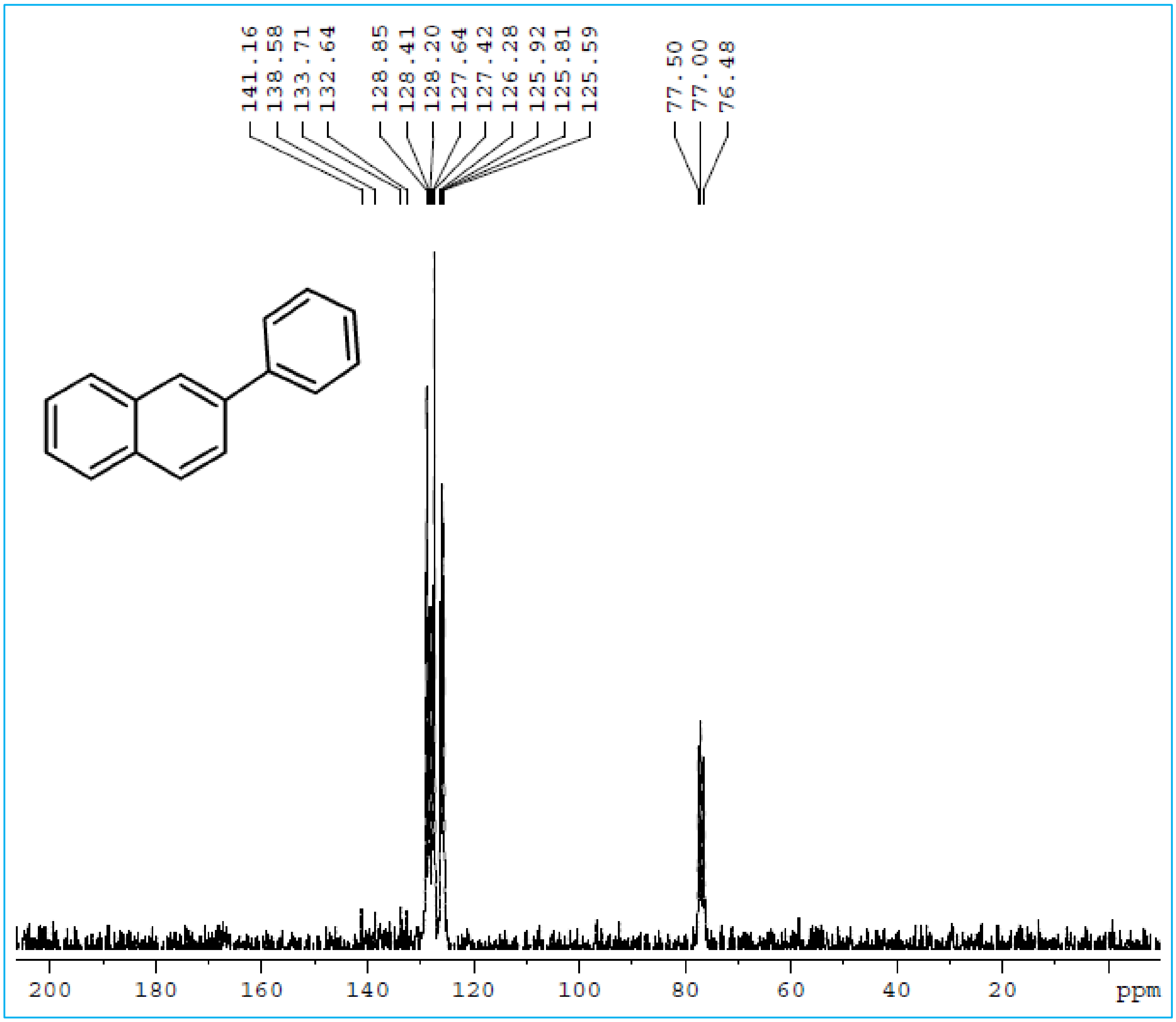
141.16
138.58
133.71
132.64
128.85
128.41
128.20
127.64
127.42
126.28
125.92
125.81
125.59
77.50
77.00
76.48
200
180
160
140
120
100
80
60
40
20
ppm

# 2-(p-tolyl)naphthalene

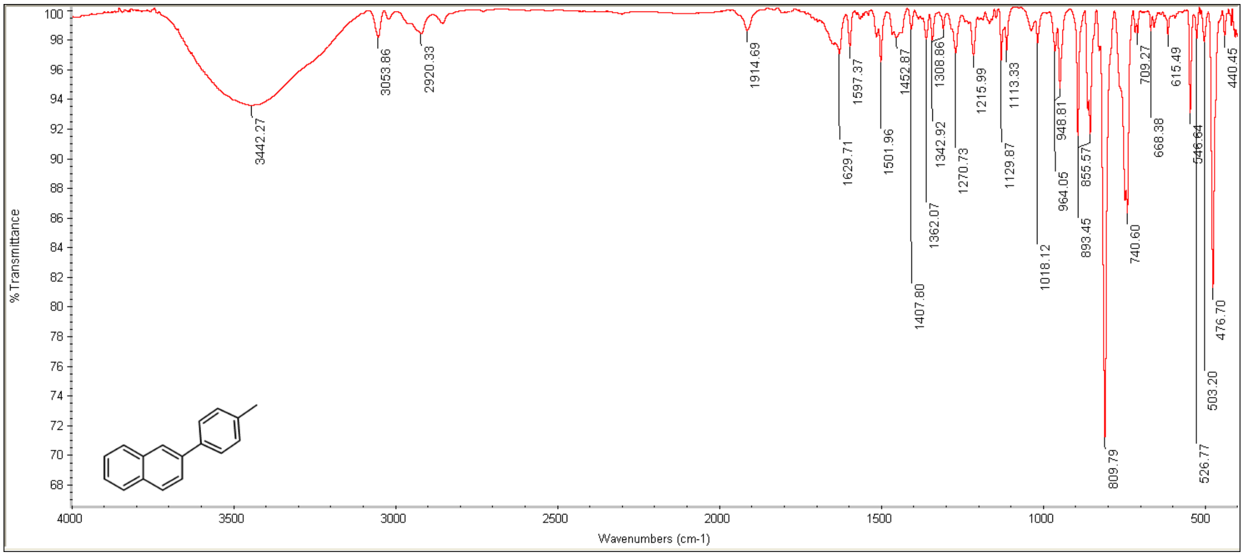


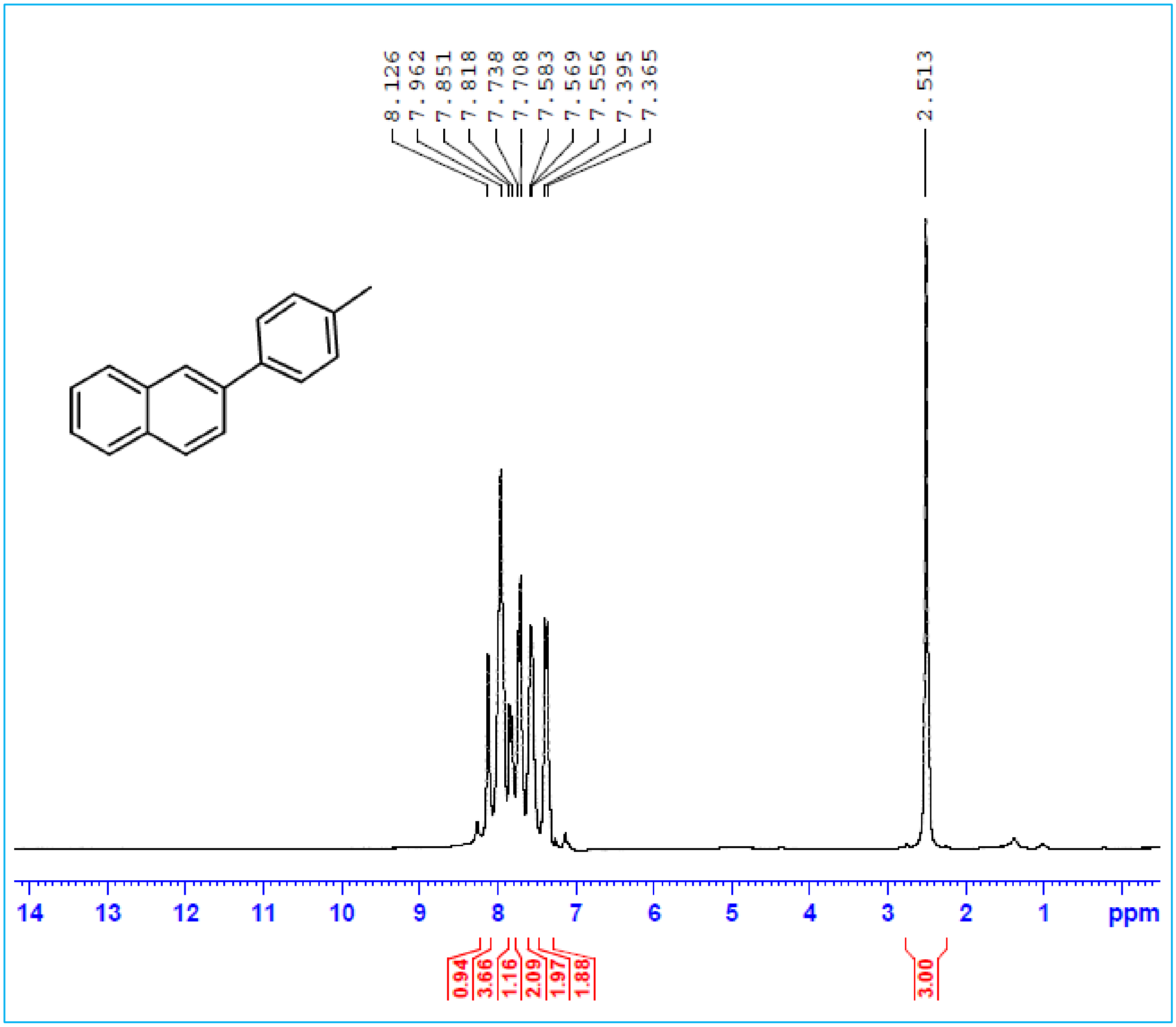

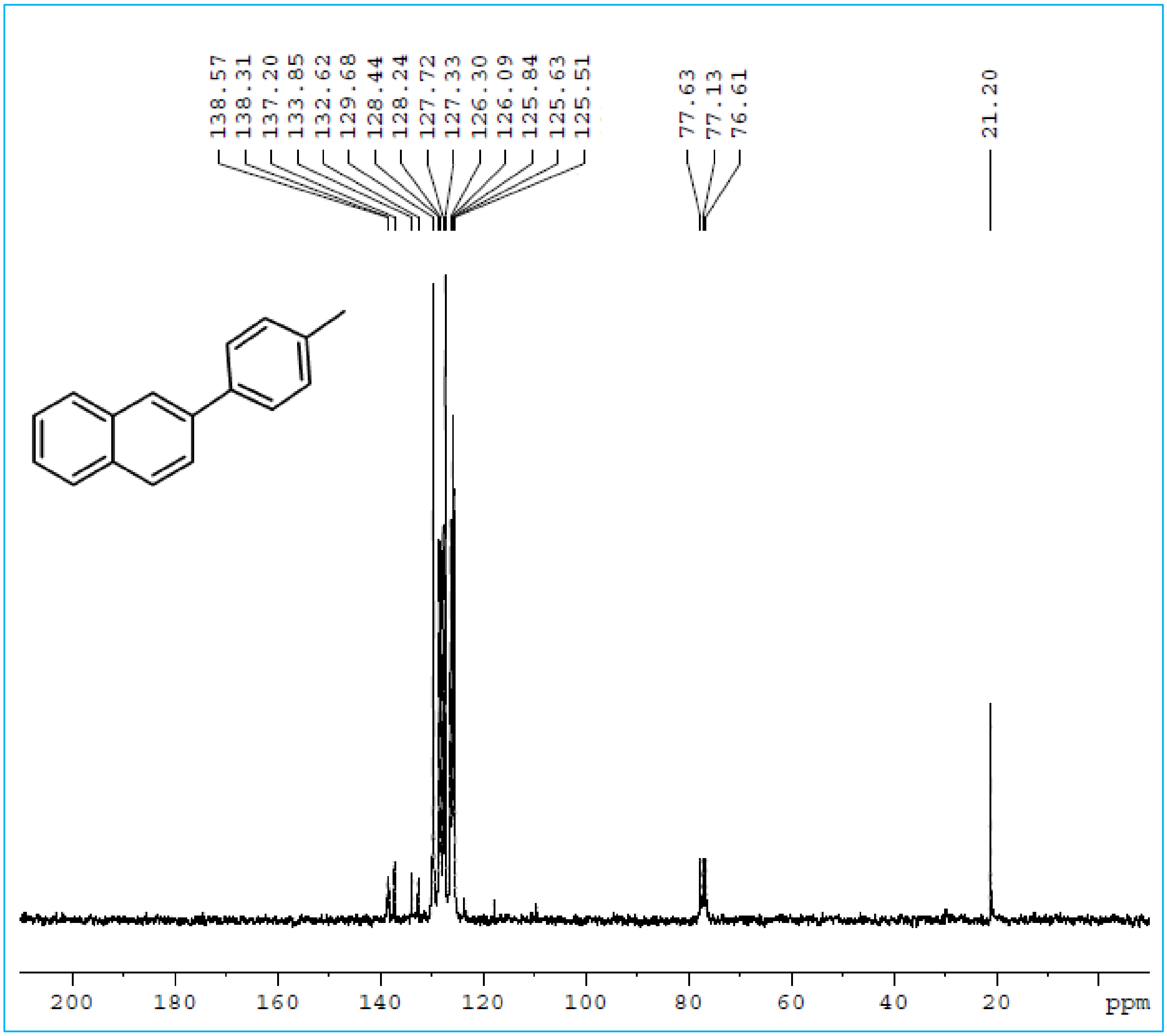
138.57
138.31
137.20
133.85
132.62
129.68
128.44
128.24
127.72
127.33
126.30
126.09
125.84
125.63
125.51
77.63
77.13
76.61
21.20
200
180
160
140
120
100
80
60
40
20
ppm

**9-phenylphenanthrene**

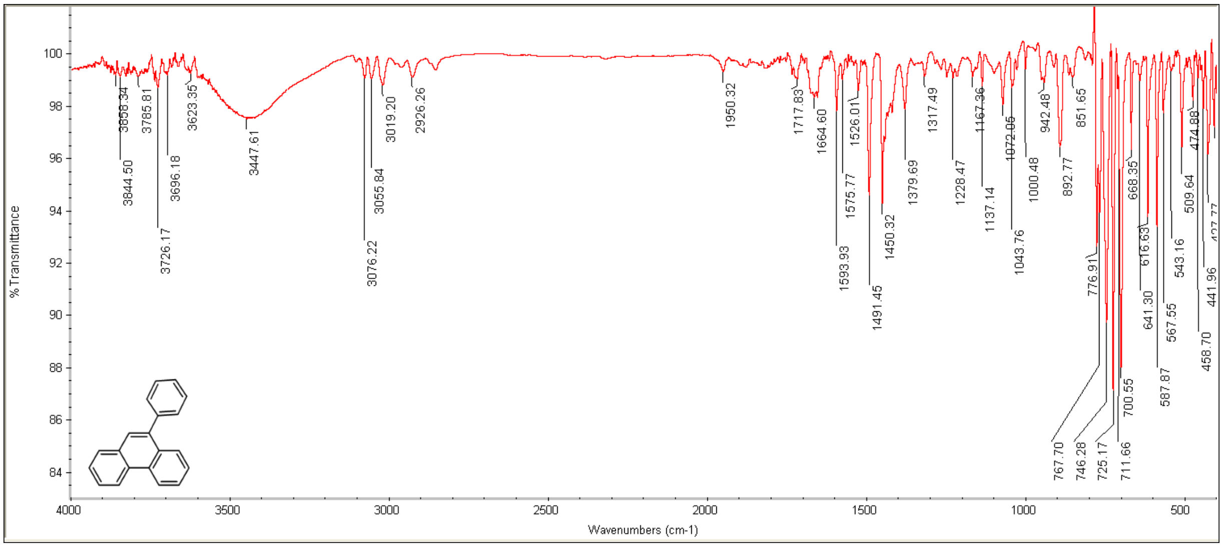


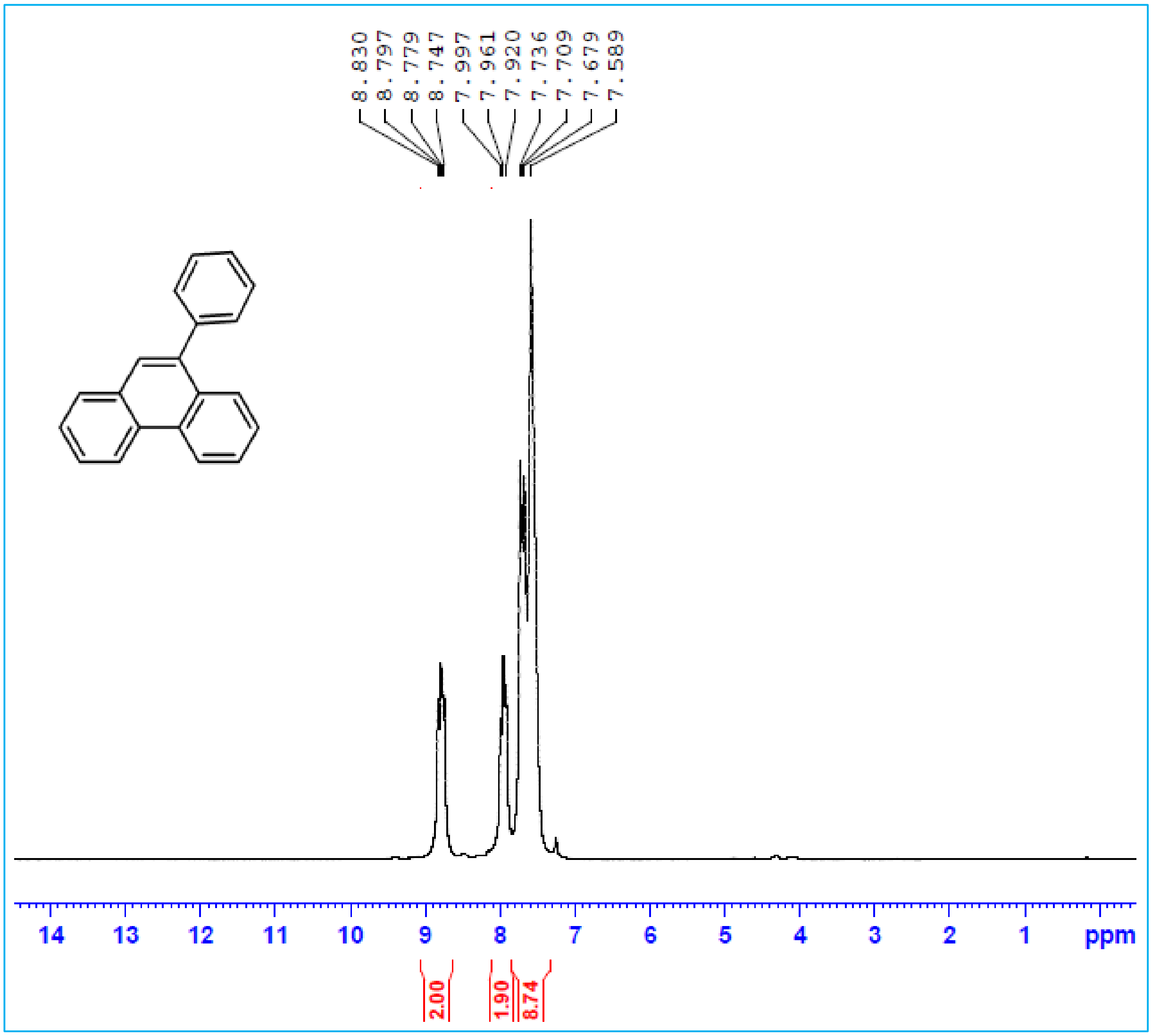

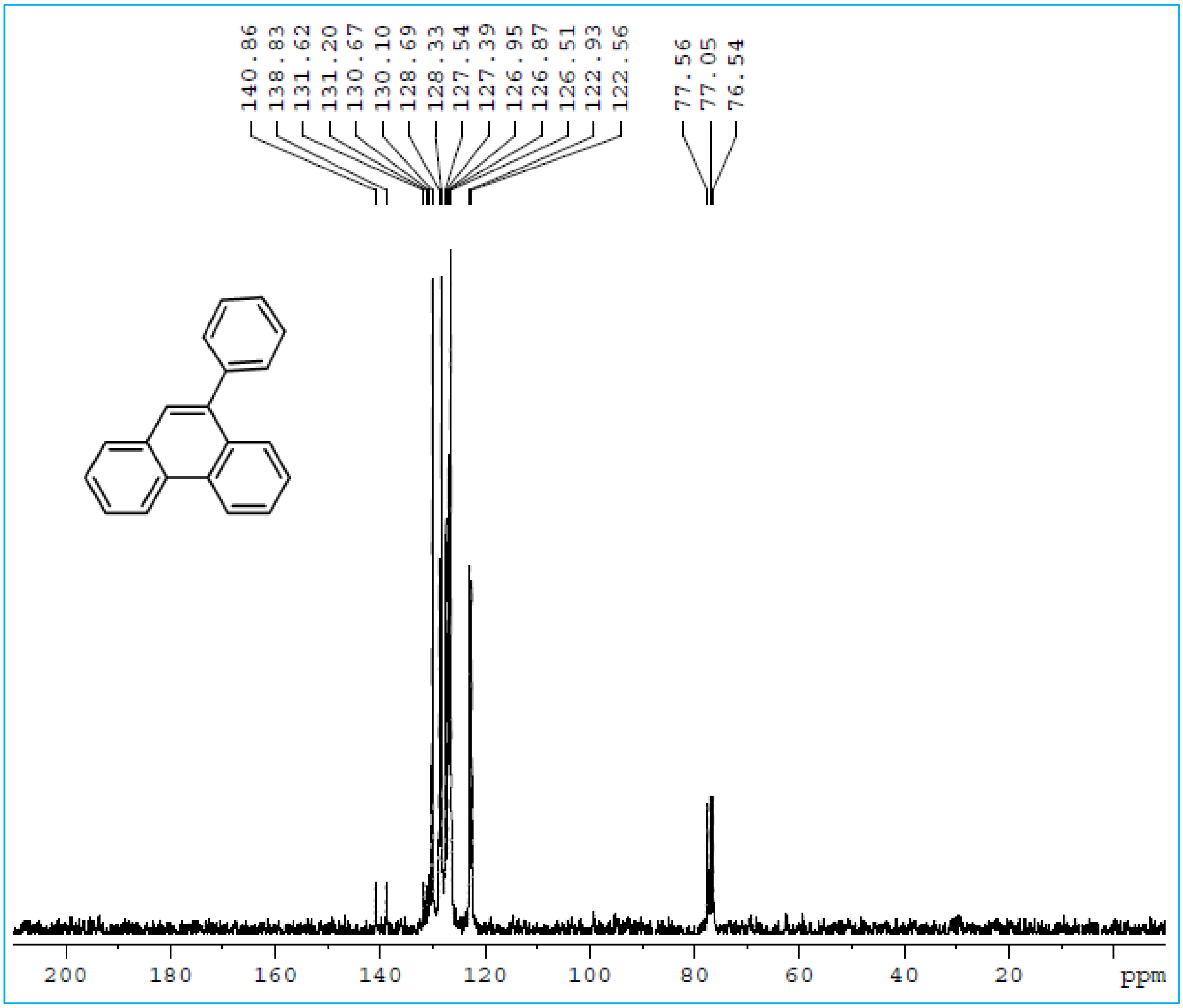
140.86
138.83
131.62
131.20
130.67
130.10
128.69
128.33
127.54
127.39
126.95
126.87
126.51
122.93
122.56
77.56
77.05
76.54
200
180
160
140
120
100
80
60
40
20
ppm

**9-(p-tolyl)anthracene**

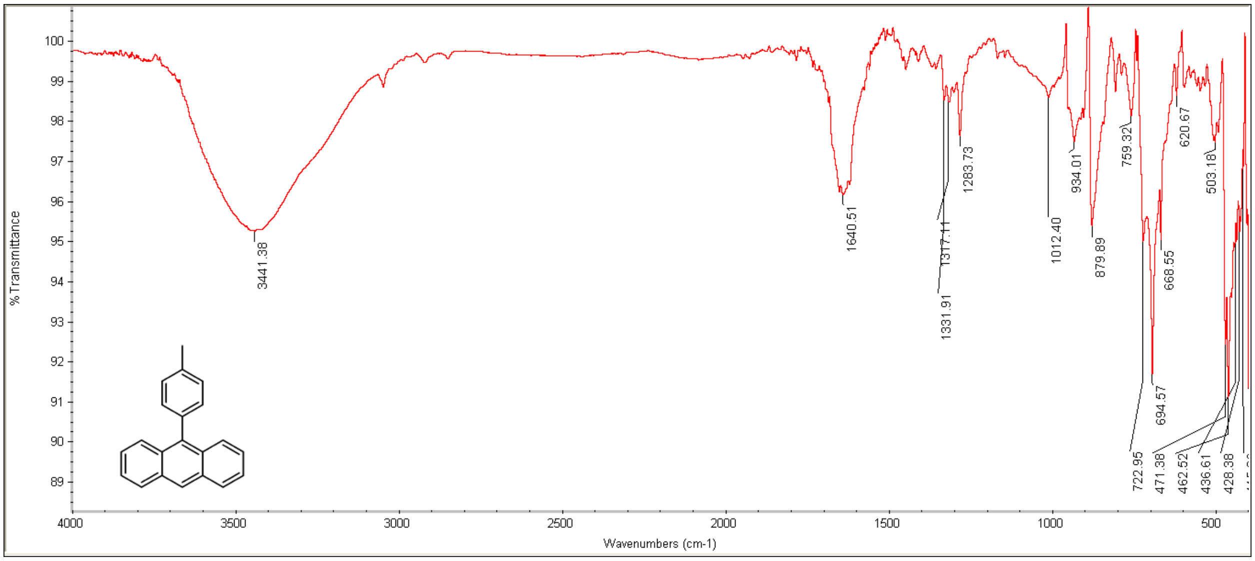


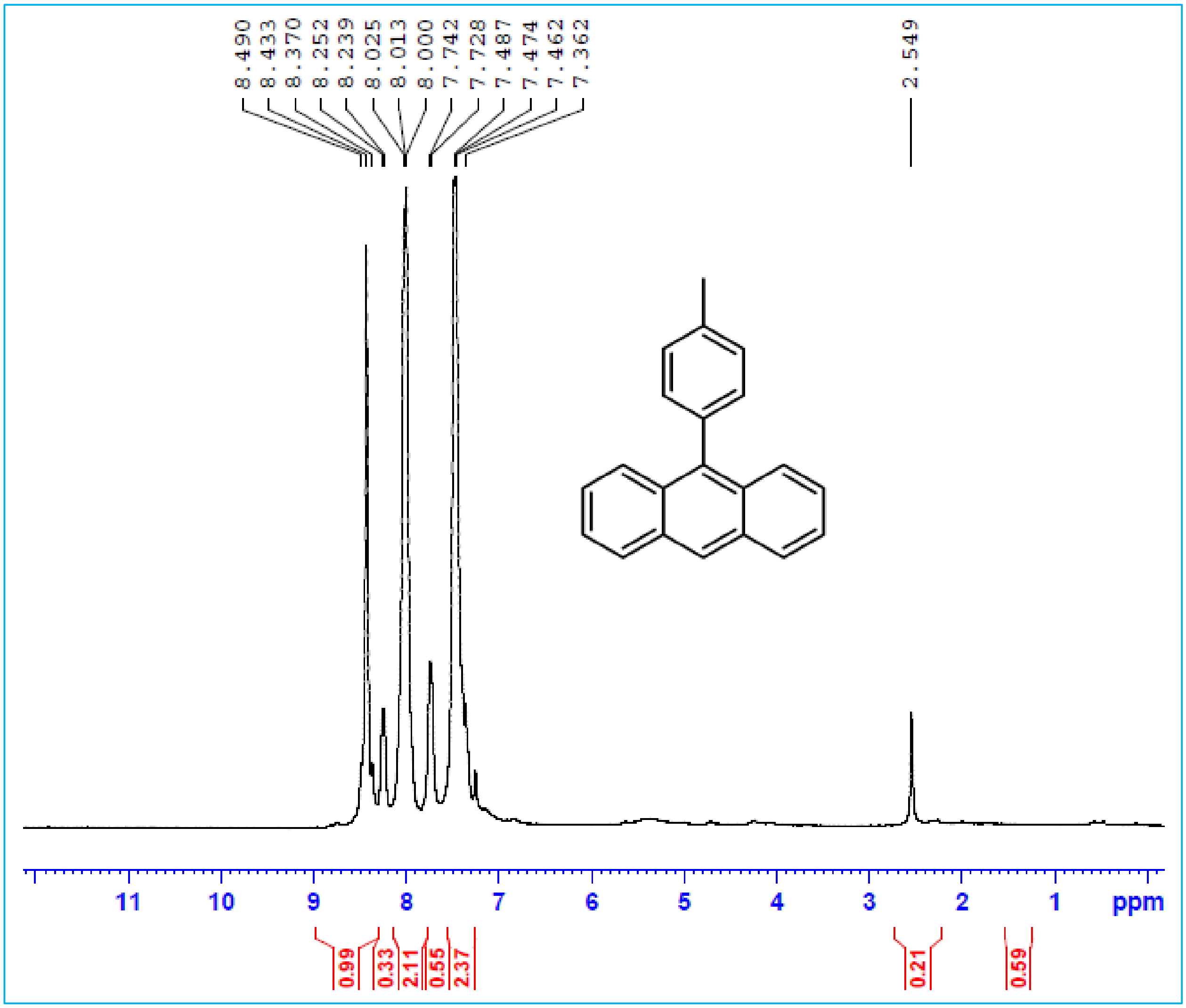

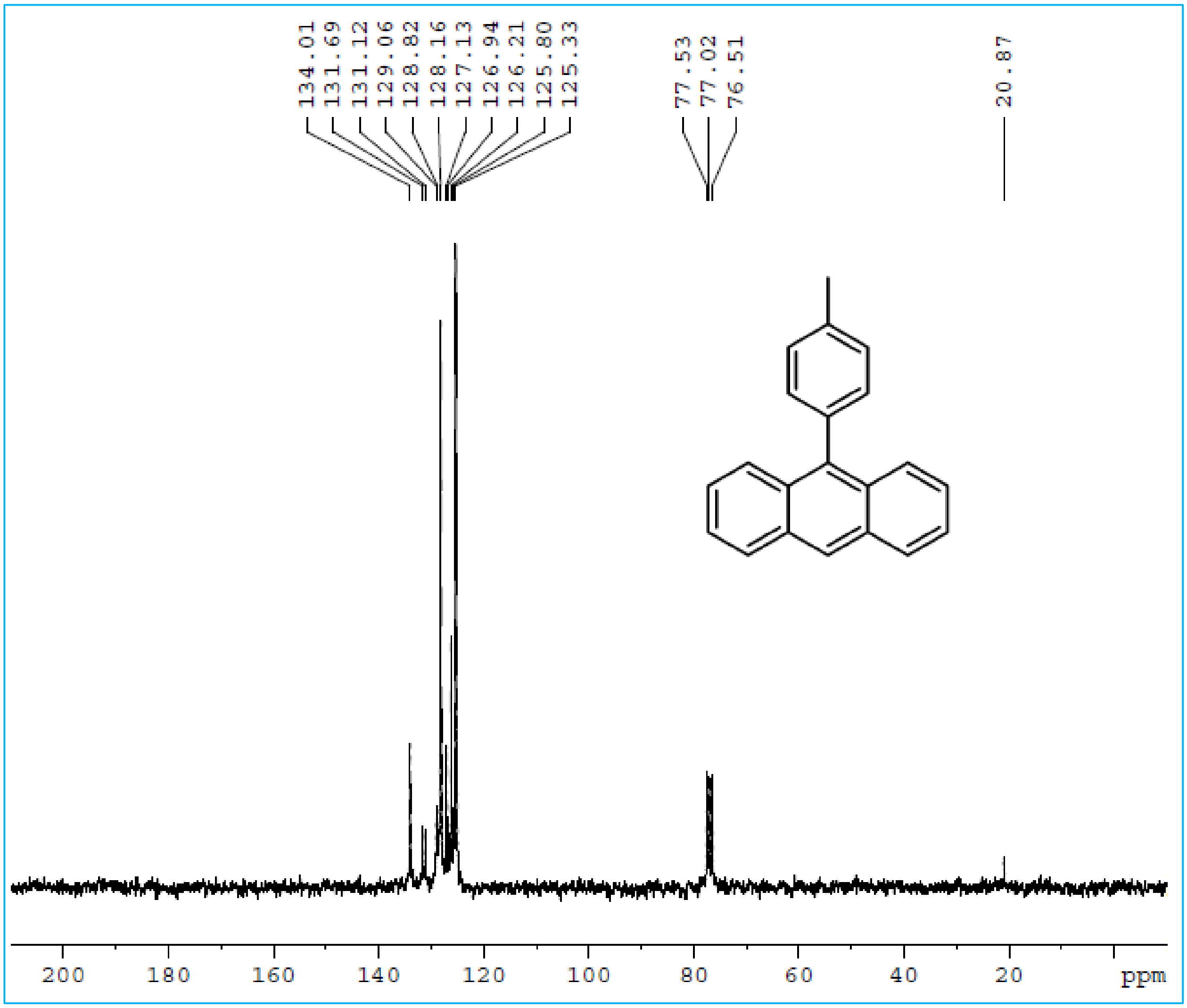

134.01
131.69
131.12
129.06
128.82
128.16
127.13
126.94
126.21
125.80
125.33
77.53
77.02
76.51
20.87
200
180
160
140
120
100
80
60
40
20
ppm

## 1,1'-biphenyl

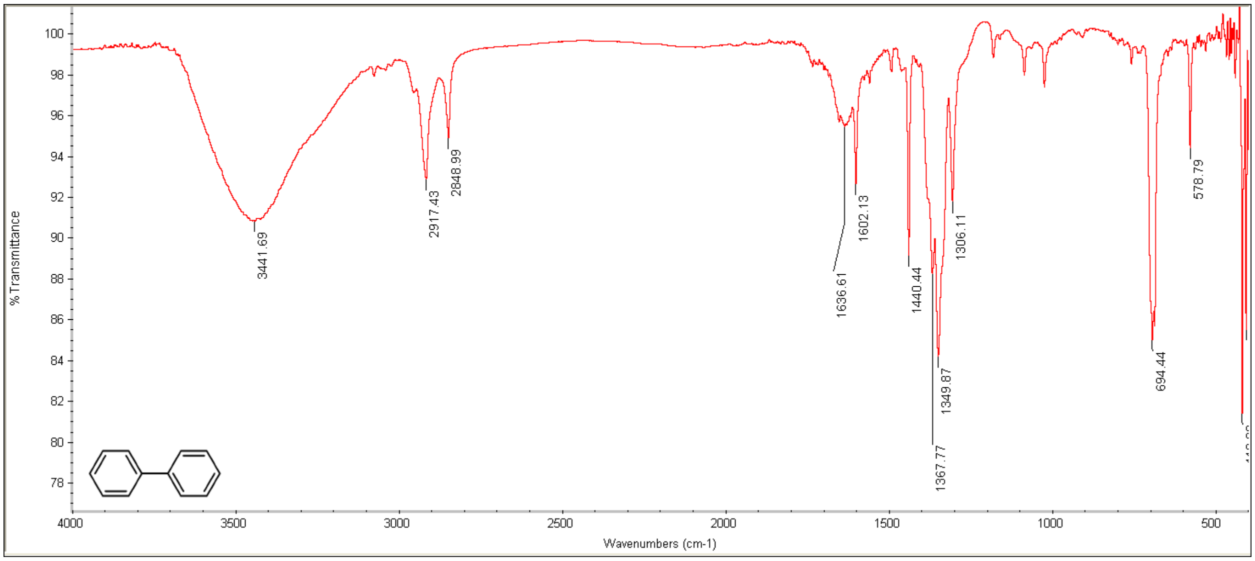

% Transmittance
Wavenumbers (cm-1)
3441.69
2917.43
2848.99
1636.61
1602.13
1440.44
1367.77
1349.87
1306.11
694.44
578.79


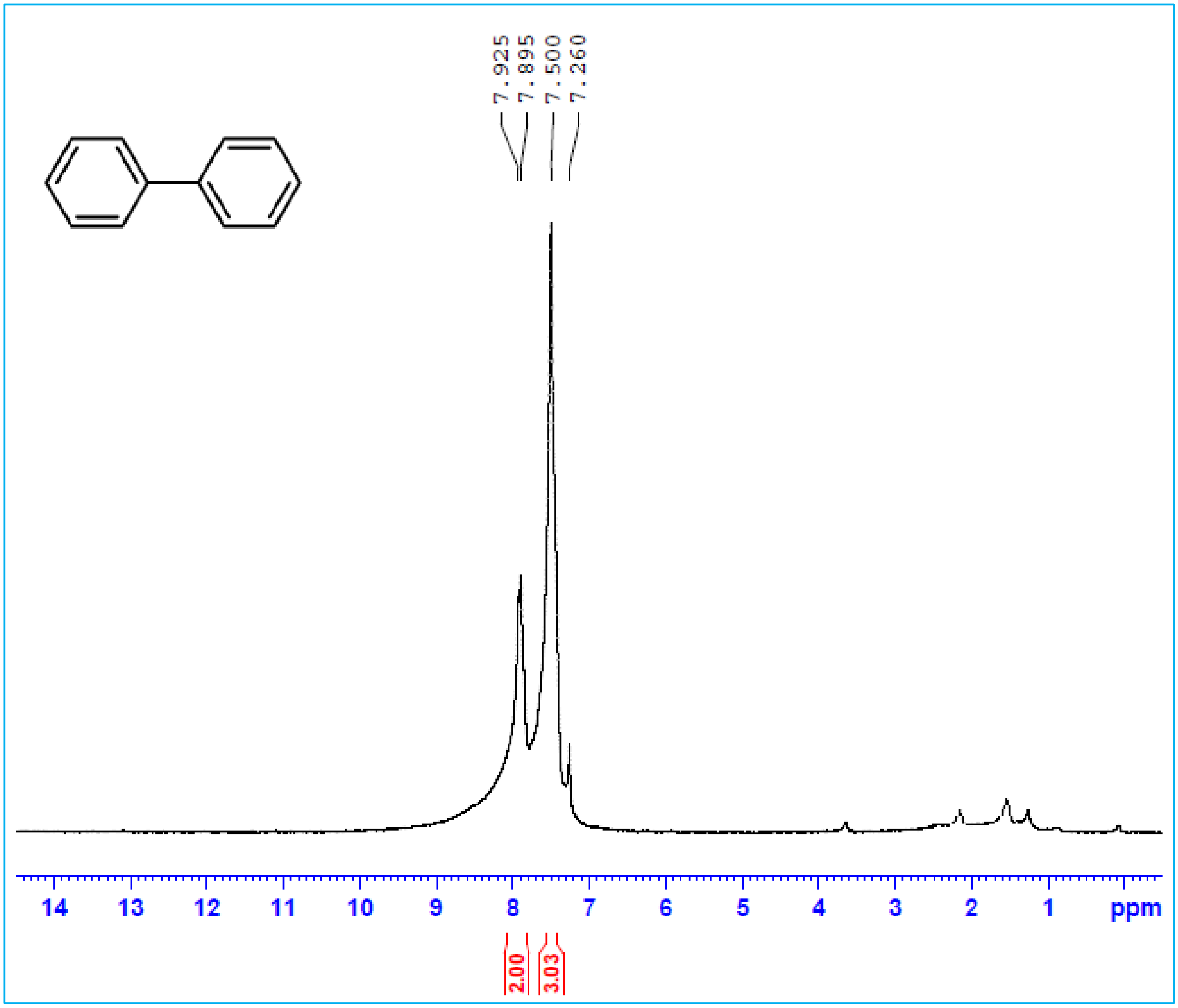

7.925
7.895
7.500
7.260
ppm
2.00
3.03

## References


[1] Ş. Yurdakul, M. Bilkana, *Optics and Spectroscopy* **2015**, *119*, 603-619.
[2] C. A. Figg, T. Kubo, B. S. Sumerlin, *ACS Macro Letters* **2015**, *4*, 1114-1118.
[3] D. Yuan, Q. Zhang, J. Dou, *Catalysis Communications* **2010**, *11*, 606-610.
[4] M. Esmaeilpour, A. Sardarian, *Green Chemistry Letters and Reviews* **2014**, *7*, 301-308.
[5] X. Meng, Z. Li, N. Yun, Z. Zhang, *Journal of Nanomaterials* **2018**, *2018*.
[6] http://www.ammrf.org.au/myscope/analysis/eds/spectralresolution/.
[7] B. Karimi, F. Mansouri, H. Vali, *Green Chemistry* **2014**, *16*, 2587-2596.
[8] aB. Karimi, A. Zamani, *Organic & biomolecular chemistry* **2012**, *10*, 4531-4536; bH. Firouzabadi, N. Iranpoor, M. Gholinejad, *Journal of Organometallic Chemistry* **2010**, *695*, 2093-2097.
[9] O. Kobayashi, D. Uraguchi, T. Yamakawa, *Organic letters* **2009**, *11*, 2679-2682.
